# Clinical, laboratory, and genetic markers for the development or presence of psoriatic arthritis in psoriasis patients: a systematic review

**DOI:** 10.1186/s13075-021-02545-4

**Published:** 2021-06-14

**Authors:** Michelle L. M. Mulder, Tamara W. van Hal, Mark H. Wenink, Hans J. P. M. Koenen, Frank H. J. van den Hoogen, Elke M. G. J. de Jong, Juul M. P. A. van den Reek, Johanna E. Vriezekolk

**Affiliations:** 1 Department of Rheumatology, Sint Maartenskliniek, PO box 9011, 6500 GM Nijmegen, The Netherlands; 2 Radboud Institute for Health Sciences (RIHS), Radboud University Medical Center, Nijmegen, The Netherlands; 3 Laboratory of Medical Immunology, Department of Laboratory Medicine, Radboud University Medical Center, Nijmegen, The Netherlands; 4 Radboud University, Nijmegen, The Netherlands; 5 Department of Dermatology, Radboud University Medical Center, Nijmegen, The Netherlands

**Keywords:** Psoriasis, Psoriatic arthritis, Systematic review, (Bio)marker, Screening, Clinical, Laboratory, Genetic

## Abstract

Twenty to thirty percent of psoriasis (Pso) patients will develop psoriatic arthritis (PsA). Detection of Pso patients that are (at risk for) developing PsA is essential to prevent structural damage. We conducted a systematic search of five bibliographic databases, up to May 2020. We searched for studies assessing markers (clinical, laboratory, genetic) associated with the development or presence of PsA in Pso patients. Study selection and quality assessment of the included studies was performed, followed by a qualitative best evidence synthesis to determine the level of evidence for a marker and its association with concomitant/developing PsA in Pso. Overall, 259 possible markers were identified in 119 studies that met the inclusion criteria. Laboratory markers related to inflammation and bone metabolism reached a strong level of evidence for the association (not prediction) of PsA in Pso. Only CXCL10 showed strong evidence for a positive predictive value for PsA in Pso. The importance of timely detecting PsA in a Pso population, and finding more (bio)markers contributing to early detection, remains high.

**Supplementary Information:**

The online version contains supplementary material available at 10.1186/s13075-021-02545-4.

## Introduction

Psoriatic arthritis (PsA) is an immune-mediated inflammatory disease affecting joints and entheses and is strongly associated with psoriasis (Pso). Twenty to thirty percent of Pso patients will develop PsA, with an average lag time between Pso and PsA of 10 years [1, 2]. This lag time creates a unique opportunity to identify patients with an increased risk for (developing) PsA. The (timely) recognition of concomitant PsA, or ideally early prediction, is important, because untreated PsA can lead to irreversible joint damage [3, 4]. Treatment of arthritis leads to an improvement of both function and quality of life [5]. However, patients with Pso are mostly seen by physicians (e.g., dermatologists) who are not trained in recognizing early signs of arthritis. Identifying markers for PsA in patients with Pso can optimize screening to detect the onset of PsA as early as possible.

Current screening strategies mostly use questionnaires based on clinical characteristics to detect PsA [6, 7]. Both characteristics of Pso as well as environmental factors may be relevant variables for PsA screening [8–10]. Next to clinical characteristics, extensive research has been done on genetic markers, in both HLA (human leukocyte antigen) and non-HLA regions [10–12]. Likewise, there are laboratory markers involved in inflammation pathways who might be able to help detect PsA in Pso patients [13, 14]. However, most research focuses on the differentiation between Pso and/or PsA on one side and healthy controls on the other side. To our knowledge, no comprehensive overview has been made to summarize the evidence for these clinical, genetic and laboratory markers.

Therefore, we conducted a systematic review to identify possible markers for the onset of PsA in a Pso population, with the purpose of providing a comprehensive summary of the available markers for PsA in Pso.

## Material and methods

### Protocol

The protocol was designed according to the Preferred Reporting Items for Systematic review and Meta-Analysis [15] and registered in Prospero (CRD42018093982).

### Search strategy

Five bibliographic databases (PubMed, EMBASE, Web of Science, Medline and Cochrane) were searched for studies from January 1, 1990, up to April 29, 2020. Search terms compromised keywords involving study population, study design, and etiology (supplementary table 1). In addition, reference lists of included articles were used for cross-reference checking.

### Study selection

Studies were screened for eligibility based on title and abstract by two independent reviewers (MM, JV for laboratory and genetic studies; MM and TH for clinical studies). Potentially relevant papers were assessed in full text (MM, TH). Any disagreement was resolved by consensus or by discussion with a third reviewer (JR, MW, JV). Studies were excluded based on the following criteria: (1) < 10 patients per group (Pso and PsA, respectively), (2) age of patients < 18 years, (3) no statistical comparison between Pso and PsA, and (4) languages other than English, German, or Dutch. We primarily focused on studies with a longitudinal design, meaning that the marker was present before the presentation of PsA. A very low number of longitudinal studies was available for laboratory studies (*n* = 2), and none for genetic studies. To not miss potential relevant markers in these two categories, we also included genetic and laboratory studies with a cross-sectional design (i.e., marker was present at the same time as PsA) as a “second best” option. While these might not be useful to identify predictors for development of PsA, they could provide information about possible markers for concomitant PsA.

### Data extraction

Data extracted included study design, patient characteristics, markers, and outcome. Extraction was performed by two reviewers, with 10% overlap to check extraction quality (MM, TH).

### Assessment of risk bias

Risk bias was assessed using the Newcastle Ottawa Scale for case-control and cohort studies [16]. This tool comprises three domains: selection, comparability, and outcome/exposure. A study was considered of “good” quality when it had a minimum of 3 stars in the selection domain, 1 star in the comparability domain, and 2 stars in the outcome/exposure domain. “Fair” quality was given when a study had a minimum of 2 stars in the selection, 1 star in the compatibility, and 2 stars in the outcome/exposure domain [17]. If a study failed to meet these standards, it was considered to be of “poor” quality. Risk of bias assessment was performed by two reviewers (MM, TH) independently. Any disagreement was resolved by consensus or by discussion with a third reviewer (JR, MW, JV).

### Best evidence synthesis

For the best evidence synthesis (BES), we included markers that either showed a significant difference between Pso and PsA in at least one study or markers that showed no significant results in at least two studies (i.e., we excluded markers who were only investigated once and showed no association). Markers were grouped into overarching categories (see Tables 1, 2 and 3). In addition, for markers presented as a categorical variable, we used the data of the most extreme level. For example, in the study from Love et al., body mass index (BMI) was categorized into four levels: < 25 (normal), 25–30, 30–35, > 35 kg/m^2^ [33]. For the best evidence synthesis, we looked at the highest level (i.e., BMI > 35 kg/m^2^) compared to reference level (i.e., BMI < 25 kg/m^2^). We then assessed the consistency of the results within and across studies. If within a study, a marker was represented in multiple non-hierarchical conceptually similar constructs, we considered the result consistent if ≥ 75% of the constructs pointed in the same direction. Otherwise, we considered the result for that marker “mixed.” For example, one study looked at fracture, any trauma, and trauma leading to medical care [21]. Because two of these were not predictive of PsA, and one was, we considered this study to have “mixed results” with respect to the marker “trauma.”

**Table 1:** Best evidence synthesis of clinical markers

Category	Marker	Good/fair quality studies	Poor quality studies	Evidence
**Comorbidities**	Diabetes mellitus	2x no association [18, 19]		Strong evidence of no association
	Diarrhea	2x no association [18, 20]	1x no association [21]	Strong evidence of no association
	Infection requiring antibiotics	1x positive association [20] 1x no association [18]		Conflicting evidence
	Uveitis	1x positive association [18]		Moderate evidence of positive association
**Disease characteristics (general)**	(worsening) Fatigue	1x positive association [22]		Moderate evidence of positive association
	Worsening function	1x positive association [22]		Moderate evidence of positive association
	Younger age at Pso onset	2x positive association [23, 24] 1x no association [25]		Conflicting evidence
**Disease characteristics (joints)**	Arthralgia in women (not men)	1x positive association [22]		Moderate evidence of positive association
	Cortical vBMD entheseal	1x negative association [26]		Moderate evidence of negative association
	Heel pain	1x positive association [22]		Moderate evidence of positive association
	(worsening) Stiffness	1x positive association [22]		Moderate evidence of positive association
	Structural entheseal lesion	1x positive association [26]		Moderate evidence of positive association
	Worsening pain	1x positive association [22]		Moderate evidence of positive association
**Disease characteristics (skin/nails)**	Duration of Pso	1x no association [27]	1x positive association [28]	Conflicting evidence
	Intergluteal lesions	1x positive association [25]		Moderate evidence of positive association
	Nail pitting	1x positive association [18]		Moderate evidence of positive association
	Psoriatic nail lesion	3x no association [18, 19, 27] 1x positive association [25]		Strong evidence of no association
	Scalp lesions	1x no association [27] 1x positive association [25]		Conflicting evidence
	Severity of Pso	2x no association [20, 27] 3x positive association [18, 22, 25]	1x positive association [28]	Conflicting evidence
**Fertility**	Fertility treatment	1x no association [20]	1x no association [21]	Moderate evidence of no association
	Hormone replacement therapy	1x no association [20]	1x no association [21]	Moderate evidence of no association
	Menopause	3x no association [18–20]		Strong evidence of no association
	Oral contraceptives	2x no association [19, 20]	1x no association [21]	Strong evidence of no association
	Pregnancy	1x no association [20] 1x negative association [19]	1x no association [21]	Conflicting evidence
**Intoxication**	Alcohol consumption	3x no association [18–20] 1x mixed results [29]	3x no association [21, 28, 30]	Strong evidence of no association
	Current smoking	2x negative association [20, 31] 2x no association [18, 29]	1x negative association [28] 1x no association [32]	Conflicting evidence
	Past smoking	3x no association [18, 29, 31] 1x negative association [20]	2x no association [28, 32]	Strong evidence of no association
	Smoking intensity		1x positive association [32]	Limited evidence of positive association
**Medication**	Corticosteroids use	1x positive association [19]		Moderate evidence of positive association
	Influenza vaccination	1x no association [20]	1x no association [21]	Moderate evidence of no association
	Methotrexate use	2x no association [18, 19]		Strong evidence of no association
	Retinoid use	1x positive association [18]		Moderate evidence of positive association
	Rubella vaccination	1x no association [20]	1x positive association [21]	Conflicting evidence
	Tetanus vaccination	1x no association [20]	1x no association [21]	Moderate evidence of no association
**Patient characteristics**	Age	4x no association [20, 22, 25, 27]		Strong evidence of no association
	BMI	3x no association [18, 22, 27] 2x positive association [29, 33]	1x positive association [34]	Conflicting evidence
	BMI at 18 years	1x positive association [24]	1x no association [34]	Conflicting evidence
	Patient reported family history of PsA	3x no association [18, 20, 27]		Strong evidence of no association
	Female sex	3x no association [20, 22, 27]	1x no association [28]	Strong evidence of no association
	Hip circumference		1x positive association [34]	Limited evidence of positive association
	University or high school level of education	1x no association [20] 1x negative association [18]		Conflicting evidence
	Waist circumference		1x positive association [34]	Limited evidence of positive association
	Waist-hip ratio		1x positive association [34]	Limited evidence of positive association
	Weight increase from 18 years		1x positive association [34]	Limited evidence of positive association
**Physical stress**	Lifting heavy loads	1x positive association [20]		Moderate evidence of positive association
	Trauma	2x no association [19, 20]	1x mixed results [21] 1x positive association [35]	Strong evidence of no association
**Psychological distress**	Anxiety/depression	2x no association [18, 20] 1x positive association [36]	1x no association [21]	Conflicting evidence
	Change in work status	1x no association [20]	1x no association [21]	Moderate evidence of no association
	Death of a family member	1x no association [20]	1x no association [21]	Moderate evidence of no association
	Move to a new house	1x no association [20]	1x positive association [21]	Conflicting evidence
	Psychological distress	1x no association [22] 1x no association [19]		Strong evidence of no association

A positive association is defined as a higher risk of PsA when the marker is present/increased/higher. A negative association is defined as a lower risk of PsA when the marker is present/increased/higher

*BMI* body mass index, *PsA* psoriatic arthritis, *Pso* psoriasis, *vBMD* volumetric bone mineral density

**Table 2:** Best evidence synthesis of laboratory markers

Category	Marker	Good/fair quality studies	Poor quality studies	Evidence
**ACPA**	Anti-CCP		3x positive association [37–39] 1x not associated [40]	Moderate evidence of positive association
	Anti-MCV		1x positive association [41]	Limited evidence of positive association
**Bone metabolism**	25(OH) vitamin D	2x no association [42, 43]	3x no association [44–46]	Strong evidence of no association
	Alkalic phosphate	1x no association [43]	2x no association [47, 48]	Moderate evidence of no association
	Calcium		2x no association [47, 48]	Moderate evidence of no association
	COMP	1x no association [49]	1x no association [50]	Moderate evidence of no association
	CPII:C2C	1x positive association [49]		Moderate evidence of positive association
	CTX		2x no association [47, 51]	Moderate evidence of no association
	DKK-1	1x no association [52]	1x positive association [53]	Conflicting evidence
	MMP3	3x positive association [49, 52, 54]	1x no association [51]	Strong evidence of positive association
	OPG	2x positive association [49, 52]	4x no association [50, 51, 53, 55]	Strong evidence of positive association
	OPG/RANKL ratio		2x negative association [50, 56]	Moderate evidence of negative association
	Osteoclast precursors		1x positive association [56]	Limited evidence of positive association
	Phosphate	1x no association [43]	1x no association [47]	Moderate evidence of no association
	RANKL	1x no association [49]	2x positive association [56, 57] 3x no association [50, 51, 53]	Conflicting evidence
	Urine Hp		1x negative association [48]	Limited evidence of negative association
**Cell culture**	IL-2 secretion		1x positive association [58]	Limited evidence of positive association
	IL-17 secretion		1x positive association [59] 1x no association [58]	Conflicting evidence
**Cytokines**	(Change in) CXCL10	1x positive association [27] 1x positive association [60]		Strong evidence of positive association
	IL-6	1x positive association [61] 1x positive association [62]	1x positive association [63] 1x no association [64]	Strong evidence of positive association
	IL-12/23 p40	1x no association [49]	1x positive association [56]	Conflicting evidence
	IL-23		1x positive association [65]	Limited evidence of positive association
	IL-33		1x positive association [56]	Limited evidence of positive association
	IL-34	1x positive association [66]	1x positive association [56]	Moderate evidence of positive association
	IL-35		1x positive association [56]	Limited evidence of positive association
	IL-36a		1x negative association [56]	Limited evidence of negative association
	IL-38		1x positive association [56]	Limited evidence of positive association
	M-CSF	1x negative association [52]	1x positive association [53]	Conflicting evidence
	TNFα		2x positive association [56, 64]	Moderate evidence of positive association
**Cytologic phenotype**	CD3+ CD71+ count		1x positive association [58]	Limited evidence of positive association
	CD4 + CD45RA-CXCR3 + CCR4-		1x negative association [67]	Limited evidence of negative association
	CD4 + CD45RA-CXCR3 + CCR6-		1x negative association [67]	Limited evidence of negative association
	CD4 + CD45RA-IFNy+		1x negative association [67]	Limited evidence of negative association
	CD4 + CD45RA-IL17+		1x positive association [67]	Limited evidence of positive association
	CD4 + T_EM_CXCR3 + CCR4-		1x negative association [67]	Limited evidence of negative association
	CD4 + T_EM_IL17A+		1x negative association [67]	Limited evidence of negative association
	CD8 + CD45RA-CCR6 + CXCR3-CD69+		1x positive association [67]	Limited evidence of positive association
	CD8 + CD45RA-IL17+		1x positive association [67]	Limited evidence of positive association
	CD8 + T_CM_CD69+		1x positive association [67]	Limited evidence of positive association
	CD8 + T_EM_IL17A+		1x positive association [67]	Limited evidence of positive association
	CD8 + T_EMRA_CCR6 + CXCR3-CD69-		1x positive association [67]	Limited evidence of positive association
	CD8 + T_EMRA_CXCR3 + CCR4-		1x negative association [67]	Limited evidence of negative association
	CD8 + T_EMRA_CXCR3 + CCR6-CD69+		1x positive association [67]	Limited evidence of positive association
	Mean platelet volume		2x positive association [68, 69]	Moderate evidence of positive association
	Monocyte count		1x positive association [70]	Limited evidence of positive association
	Neutrophil count		1x positive association [70]	Limited evidence of positive association
	Neutrophil to lymphocyte ratio		1x positive association [70]	Limited evidence of positive association
	Platelet count		1x positive association [70]1x no association [68]	Conflicting evidence
	Platelet to lymphocyte ratio		1x positive association [70]	Limited evidence of positive association
	White blood count		1x positive association [70] 1x no association [46]	Conflicting evidence
**Inflammation marker**	CRP	5x positive association [43, 49, 54, 66, 71] 1x no association [27]	8x positive association [44, 47, 53, 56, 70, 72–74] 4x no association [46, 58, 64, 75]	Strong evidence of positive association
	ESR	1x positive association [66] 1x no association [43]	5x positive association [44, 47, 56, 70, 74] 2x no association [62, 75]	Conflicting evidence
**Lipid metabolism**	Adiponectin	1x positive association [71]	1x negative association [64]	Conflicting evidence
	ApoA to ApoB ratio		1x positive association [76]	Limited evidence of positive association
	ApoB		1x positive association [76]	Limited evidence of positive association
	CER		1x positive association [46]	Limited evidence of positive association
	Glucose	2x no association [42, 71]	4x no association [46, 62, 76, 77]	Strong evidence of no association
	HDL	2x no association [42, 71]	3x no association [62, 72, 77]	Strong evidence of no association
	Insulin		1x negative association [77]	Limited evidence of negative association
	LDL	2x no association [42, 71]	3x no associated [46, 72, 76] 1x positive association [62]	Strong evidence of no association
	LDL:HDL ratio		2x positive association [62, 76]	Moderate evidence of positive association
	Leptin	1x positive association [71]	1x no association [64]	Conflicting evidence
	Total cholesterol	1x negative association [42] 1x no association [71]	2x no association [76, 77] 1x positive association [62]	Conflicting evidence
	Total cholesterol/HDL	1x no association [42]	1x positive association [76]	Conflicting evidence
	Triglycerides	2x no association [42, 71]	4x no association [42, 46, 76, 77] 2x positive association [62, 72]	Strong evidence of no association
	VLDL		2x no association [62, 76]	Moderate evidence of no association
**miRNA expression**	let-7b-3p	1x negative association [78]		Moderate evidence of negative association
	let-7b-5p	1x negative association [78]		Moderate evidence of negative association
	let-7e-5p	1x positive association [78]		Moderate evidence of positive association
	miR-26a-5p	1x positive association [78]		Moderate evidence of positive association
	miR-27a-3p	1x positive association [78]		Moderate evidence of positive association
	miR-27b-3p	1x positive association [78]		Moderate evidence of positive association
	miR-29a-3p	1x positive association [78]		Moderate evidence of positive association
	miR-30e-5p	1x positive association [78]		Moderate evidence of positive association
	miR-92a-3p	1x negative association [78]		Moderate evidence of negative association
	miR-92b-3p	1x negative association [78]		Moderate evidence of negative association
	miR-98-5p	1x positive association [78]		Moderate evidence of positive association
	miR-139-3p	1x negative association [78]		Moderate evidence of negative association
	miR-146a-5p	1x positive association [78]	1x positive association [79]	Moderate evidence of positive association
	miR-203a	1x negative association [78]		Moderate evidence of negative association
	miR-486-5p	1x negative association [78]		Moderate evidence of negative association
	miR-1180-3p	1x negative association [78]		Moderate evidence of negative association
	miR-2379-5p	1x positive association [78]		Moderate evidence of positive association
	miR-3158-3p	1x negative association [78]		Moderate evidence of negative association
	miR-4732-3p	1x negative association [78]		Moderate evidence of negative association
**mRNA expression whole blood**	CCL1	1x negative association [80]		Moderate evidence of negative association
	CCL7	1x negative association [80]		Moderate evidence of negative association
	CCL20	1x negative association [80]		Moderate evidence of negative association
	CX3CL1	1x negative association [80]		Moderate evidence of negative association
	CXCL2	1x negative association [80]		Moderate evidence of negative association
	CXCL5	1x negative association [80]		Moderate evidence of negative association
	HAT1		1x positive association [81]	Limited evidence of positive association
	IL-3	1x negative association [80]		Moderate evidence of negative association
	IL-6	1x negative association [80]		Moderate evidence of negative association
	IL-8	1x negative association [80]		Moderate evidence of negative association
	IL-17C	1x negative association [80]		Moderate evidence of negative association
	IL-17F	1x negative association [80]		Moderate evidence of negative association
	ISG20	1x negative association [80]		Moderate evidence of negative association
	MMP-3	1x negative association [80]		Moderate evidence of negative association
	NOTCH2NL		1x negative association [81]	Limited evidence of negative association
	SET2D		1x negative association [81]	Limited evidence of negative association
	STAT3	1x negative association [80]		Moderate evidence of negative association
	STAT6	1x negative association [80]		Moderate evidence of negative association
	SYK	1x negative association [80]		Moderate evidence of negative association
	TBX21	1x negative association [80]		Moderate evidence of negative association
**Serum**	CD5L	1x positive association [54]		Moderate evidence of positive association
	Creatinine	1x no association [43]	1x no association [53]	Moderate evidence of no association
	Complement C9		1x negative association [82]	Limited evidence of negative association
	IFI16	1x negative association [83]		Moderate evidence of negative association
	sIL2R	1x positive association [61]		Moderate evidence of positive association
	ITGB5	1x positive association [54]		Moderate evidence of positive association
	Gelsolin		1x negative association [44]	Limited association of negative association
	K17		1x positive association [84]	Limited evidence of positive association
	M2BP	1x positive association [54]		Moderate evidence of positive association
	MPO	1x positive association [54]		Moderate evidence of positive association
	PRL		1x positive association [85]	Limited evidence of positive association
	STIP1		1x positive association [84]	Limited evidence of positive association
	Uric acid	1x positive association [86] 1x no association [87]	1x no association [88] 1x negative association [77]	Conflicting evidence
	VCP		1x positive association [89]	Limited evidence of positive association
	VEGFR-3		1x positive association [90]	Limited evidence of positive association
	YKL-40		1x positive association [91]	Limited evidence of positive association
**Skin**	C16ORF61		1x positive association [92]	Limited evidence of positive association
	CPN2		1x positive association [92]	Limited evidence of positive association
	CXCL12		1x positive association [93]	Limited evidence of positive association
	FHL1		1x positive association [92]	Limited evidence of positive association
	GPS1		1x positive association [92]	Limited evidence of positive association
	IL23R		1x positive association [94]	Limited evidence of positive association
	ITGB5		1x positive association [92]	Limited evidence of positive association
	POSTN		1x positive association [92]	Limited evidence of positive association
	PP2R4		1x positive association [92]	Limited evidence of positive association
	SNCA		1x positive association [92]	Limited evidence of positive association
	SRP14		1x positive association [92]	Limited evidence of positive association
	SRPX		1x positive association [92]	Limited evidence of positive association
**Miscellaneous**	Anti-ADAMTS-L5 IgG antibodies		1x positive association [95]	Limited evidence of positive association
	Anti-LL37 antibodies		1x positive association [95] 1x mixed results [82]	Conflicting evidence
	Arylesterase activity		1x positive association [72]	Limited evidence of positive association
	Hemoglobin		1x negative association [70]	Limited evidence of negative association
	IgG response to C region of rM12 protein		1x positive association [96]	Limited evidence of positive association

A positive association is defined as a higher risk of PsA when the marker is present/increased/higher. A negative association is defined as a lower risk of PsA when the marker is present/increased/higher

*ACPA* anti citrullinated protein antibodies, *ADAMTS* a disintegrin and metalloproteinase with thrombospondin motifs; anti-CCP, anti-cyclic citrullinated protein; *Apo* apolipoprotein, *C16ORF61* endosomal protein sorting factor like (VSP35L), *C2C* collagen fragment neoepitopes Col2-3/4 (long mono), *CCL* C-C chemokine ligand, *CCR* C-C chemokine receptor, *CD*, cluster of differentiation, *CD5L* CD5 ligand, *CER* ceramide, *CM* central memory, *COMP* cartilage oligomeric matrix protein, *CPII* C-propeptide of type II collagen, *CPN2* carboxypeptidase N subunit 2, *CRP* C-reactive protein, *CTX* collagen type I C-telopeptide, *CX3CL* C-X3-C motif ligand, *CXCL* C-X-C motif ligand, *CXCR* C-X-C motif receptor, *DKK* Dickkopf, *EM* effector memory, *ESR* erythrocyte sedimentation rate, *FHL1* four and a half LIM domains, *GPS* G protein pathway suppressor, *HAT* human airway trypsin-like protein, *HDL* high-density lipoprotein, *Hp* hydroxyproline, *IFI* interferon-inducible protein, *IFN* interferon, *IgG* immunoglobulin G, *IL* interleukin, *IL23R* IL23 receptor, *ISG* interferon stimulated gene, *ITGB* integrin beta, K17, keratin 17, *LDL* low-density lipoprotein, *M2BP* Mac-2-binding protein, *M-CSF* macrophage colony-stimulating factor, *MCV* mutated citrullinated vimentin, *miRNA* micro RNA, *MMP* matrix metalloproteinase, *MPO* myeloperoxidase, *mRNA* messenger RNA, *OPG* osteoprotegerin, *POSTN* periostin, *PPP2R4* protein phosphatase 2 phosphatase activator (PTPA);PRL, prolactin, *RANKL* receptor activator of nuclear factor kappa-B ligand, *RNA* ribonucleic acid, *SETD* SET domain protein, *sIL-2R* soluble IL-2 receptor, *SNCA* synuclein alpha, *SRP* signal recognition particle, *SRPX* sushi repeat containing protein X-linked, *STAT* signal transducer and activator of transcription, *STIP* stress-inducible phosphoprotein, *SYK* spleen-associated tyrosine kinase, *TBX* T-box, *TNF* tumor necrosis factor, *VCP* valosin-containing protein, *VEGFR* vascular endothelial growth factor receptor, *VLDL* very low-density lipoprotein

**Table 3:** Best evidence synthesis of genetic markers

Category	Marker	Good/fair quality studies	Poor quality studies	Evidence
**HLA**	Haplotype B*08:01-C*07		1x positive association [97]	Limited evidence of positive association
	Haplotype B*08-C*07-MICA*00801	1x positive association [98]		Moderate evidence of positive association
	Haplotype B*18-C*07		1x positive association [99]	Limited evidence of positive association
	Haplotype B*27-C*01		2x positive association [97, 99]	Moderate evidence of positive association
	Haplotype B*27-C*02		3x positive association [97, 99, 100]	Moderate evidence of positive association
	Haplotype B*27-C*02-MICA*00701/026	1x positive association [98]		Moderate evidence of positive association
	Haplotype B*35-C*04-MICA*0201/020	1x negative association [98]		Moderate evidence of negative association
	Haplotype B*37-C*06		1x negative association [97]	Limited evidence of negative association
	Haplotype B*38-C*12		3x positive association [97, 99, 100]	Moderate evidence of positive association
	Haplotype B*39:01-C*12		2x positive association [97, 100]	Moderate evidence of positive association
	Haplotype B*57-C*06		2x negative association [97, 99]	Moderate evidence of negative association
	Haplotype B*57-C*06-MICA*017		1x negative association [99]	Limited evidence of negative association
	HLA-A*03		1x mixed results [101]	Conflicting evidence
	HLA-B*08		2x positive association [97, 99] 3x no association [100, 102, 103]	Conflicting evidence
	HLA-B*13		1x mixed results [101] 2x no association [102, 104]	Conflicting evidence
	HLA-B*18		1x positive association [97] 1x no association [100]	Conflicting evidence
	HLA-B*27		6x positive association [97, 99, 100, 103–105] 1x no association [102]	Moderate evidence of positive association
	HLA-B*37		1x negative association [97] 1x no association [102]	Conflicting evidence
	HLA-B*38		3x positive association [97, 99, 100] 1x no association [104] 1x mixed results [101]	Conflicting evidence
	HLA-B*39		1x positive association [100] 1x mixed results [97]	Conflicting evidence
	HLA-B*40		1x negative association [97]	Limited evidence of negative association
	HLA-B*44		1x negative association [97]	Limited evidence of negative association
	HLA-B*57		1x negative association [99] 3x no association [100, 102, 104]	Moderate evidence of no association
	HLA-B*70		1x mixed results [101]	Conflicting evidence
	HLA-B amino acid position 45 Glu		1x positive association [106] 2x no association [102, 103]	Conflicting evidence
	HLA-B amino acid position 95 Leu		1x positive association [102]	Limited evidence of positive association
	HLA-B amino acid position 97 Arg		1x mixed results [103] 1x no association [102]	Conflicting evidence
	HLA-C*01		1x positive association [99] 3x no association [97, 100, 102]	Moderate evidence of no association
	HLA-C*02		2x positive association [97, 99] 2x no association [100, 102]	Conflicting evidence
	HLA-C*06	1x negative association [107]	7x negative association [97, 99, 102–105, 108] 2x no association [100, 109] 1x mixed results [101]	Moderate evidence of negative association
	HLA-C*07		1x positive association [99] 2x no association [100, 102]	Conflicting evidence
	HLA-C*08		1x negative association [105]	Limited evidence of negative association
	HLA-C*12		1x positive association [100] 2x no association [99]	Conflicting evidence
	HLA-C amino acid position 305 Ala		1x positive association [102]	Limited evidence of positive association
	HLA-C rs10484554		1x positive association [110]	Limited evidence of positive association
	HLA-C rs12191877		1x negative association [111]	Limited evidence of negative association
	HLA-DQB1*02		1x mixed results [101] 1x no association [102]	Conflicting evidence
	HLA-DRB1*03		2x no association [101, 102]	Moderate evidence of no association
	HLA-DR*04		1x positive association [101]	Limited evidence of positive association
	HLA-DR*07		1x negative association [105]	Limited evidence of negative association
	HLA-DR*11		1x mixed results [101]	Conflicting evidence
**Non-HLA**	ADAMTS9-MAG1 deletion		1x positive association [112]	Limited evidence of positive association
	CCR2 rs1799864	1x positive association [113]		Limited evidence of positive association
	IL1RN rs397211		2x no association [111, 114]	Moderate evidence of no association
	IL12B rs2082412		2x negative association [111, 114]	Moderate evidence of negative association
	IL12B rs3212227	1x no association [115]	1x no association [109]	Moderate evidence of no association
	IL12B rs6887695	1x no association [115]	1x no association [109]	Moderate evidence of no association
	IL13 rs1800925	1x positive association [116]	1x positive association [117]	Moderate evidence of positive association
	IL13 rs20541		2x positive association [114, 117] 1x not associated [111]	Conflicting evidence
	IL13 rs848	1x positive association [116]		Moderate evidence of positive association
	IL17E rs79877597		1x positive association [118]	Limited evidence of positive association
	IL23A rs2066807		2x not associated [111, 114]	Moderate evidence of no association
	IL23R rs11209026	1x no association [115]	1x no association [109]	Moderate evidence of no association
	IL23R rs2201841		1x negative association [111] 1x not associated [114]	Conflicting evidence
	KIR2DS1 pos/C2 neg		1x positive association [119]	Limited evidence of positive association
	LOC100505817 rs4891505		1x positive association [120]	Limited evidence of positive association
	MICA*00701/026	1x positive association [98]		Moderate evidence of positive association
	MICA*00801	1x positive association [98]		Moderate evidence of positive association
	MICA*016	1x negative association [98]		Moderate evidence of negative association
	NFKBIA rs7152376	1x positive association [107]		Moderate evidence of positive association
	PTPN22 rs2476601		1x positive association [121]	Limited evidence of positive association
	TNFa-238		2x not associated [109, 122]	Moderate evidence of no association
	TNFa-308		2x not associated [109, 122]	Moderate evidence of no association
	TNFa-857		1x positive association [109]	Limited evidence of positive association
	TNFacd haplotype a6c1d3		1x positive association [123]	Limited evidence of positive association
	TNFAIP3 rs610604		2x not associated [111, 114]	Moderate evidence of no association
	TNIP rs17728338		2x not associated [111, 114]	Moderate evidence of no association
	TRAF3IP2 rs240993		1x not associated [114]	Limited evidence of no association
	TRAF3IP2 rs458017		1x not associated [110]	Limited evidence of no association
	TSC1 rs1076160		2x not associated [111, 114]	Moderate evidence of no association
	ZNF816A		1x negative association [114]	Limited evidence of negative association

A positive association is defined as a higher risk of PsA when the marker is present/increased/higher. A negative association is defined as a lower risk of PsA when the marker is present/increased/higher.

*ADAMTS* a disintegrin and metalloproteinase with thrombospondin motifs, *Arg* arginine, *CCR* C-C motif receptor, *Glu* glutamic acid, *HLA* human leukocyte antigen, *IL* interleukin, *IL1RN* IL-1 receptor antagonist, *IL23R* IL-23 receptor, *KIR* killer-cell immunoglobulin-like receptor, *MAGI* membrane-associated guanylate kinase, *MICA* MHC class I polypeptide-related sequence A, *PTPN22* protein tyrosine phosphatase non-receptor type 22, *TNF* tumor necrosis factor, *TNFAIP* TNF alpha-induced protein, *TNIP* TNFAIP3-interacting protein, *TRAF* TNF receptor-associated factor, *TRAF3IP* TRAF3-interacting protein, *TSC1* tuberous sclerosis 1, *ZNF* zinc finger protein

If across multiple studies, < 75% of studies were in agreement with each other, we considered this “conflicting evidence.” If ≥ 75% of studies were in agreement, we applied the evidence grading according to Sackett [17]. Because only a small minority of the included studies were of “good” quality, we adapted the Sackett best evidence synthesis as follows: strong evidence in case of two or more studies with good or fair quality, moderate evidence in case of two or more studies with low quality or one study of good or fair quality, and limited evidence in case of one study with low quality. In case of two or more good/fair quality studies, the results of the poor quality studies were not taken into account for the BES. The heterogeneity of the markers and statistics precluded a quantitative meta-analysis.

## Results

### Study selection

**Fig. 1:**
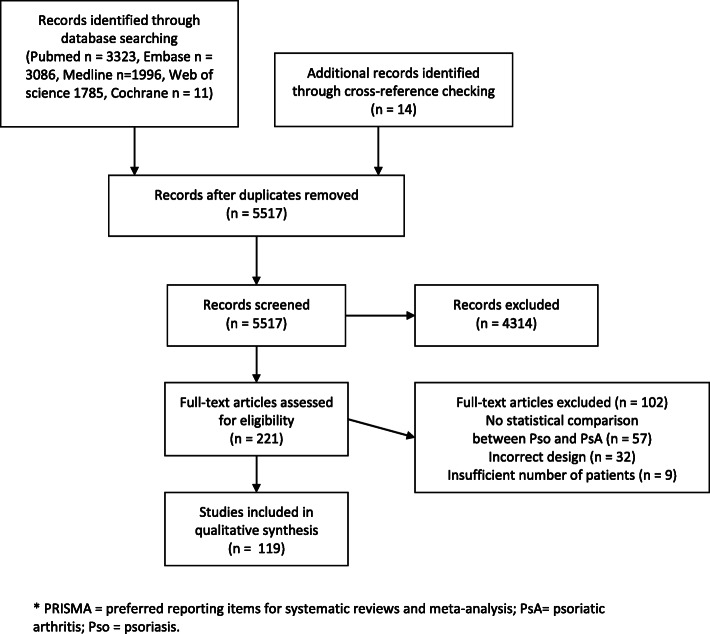
PRISMA flowchart of included studies. PRISMA, preferred reporting items for systematic reviews and meta-analysis; PsA, psoriatic arthritis; Pso, psoriasis

The search yielded 5517 non-duplicate articles and, in addition, 14 studies were included via cross-reference checking. After screening on title and abstract, 221 articles were assessed in full text. A total of 119 studies met the selection criteria and were included. Of these, 19 studied clinical markers [18–36], 69 studied laboratory markers [27, 37, 38, 40–55, 57–73, 75–96, 124–133], and 32 studied genetic markers [97–113, 115–123, 134–139]. One study described both clinical and laboratory markers [27]. A flow chart of the selection process is shown in Fig. 1.

### Study characteristics

The characteristics of the included studies are listed in supplementary table 2. All clinical studies had a longitudinal design. Two laboratory studies had a longitudinal design and 67 had a cross-sectional design. All of the genetic studies had a cross-sectional design. Based on the criteria described in the best evidence synthesis, 259 markers were selected for further description (clinical 51, laboratory 137, genetic 71), of which 104 were described in multiple studies (clinical 32, laboratory 36, genetic 36). All markers are shown in supplementary tables 3, 4, 5.

### Quality assessment

Of the included studies, 19 studies were qualified as good quality, 11 studies were qualified as fair quality, and 89 studies were qualified as poor quality. Quality assessment of the included studies is shown in supplementary tables 6 and 7.

### Best evidence synthesis

**Fig. 2:**
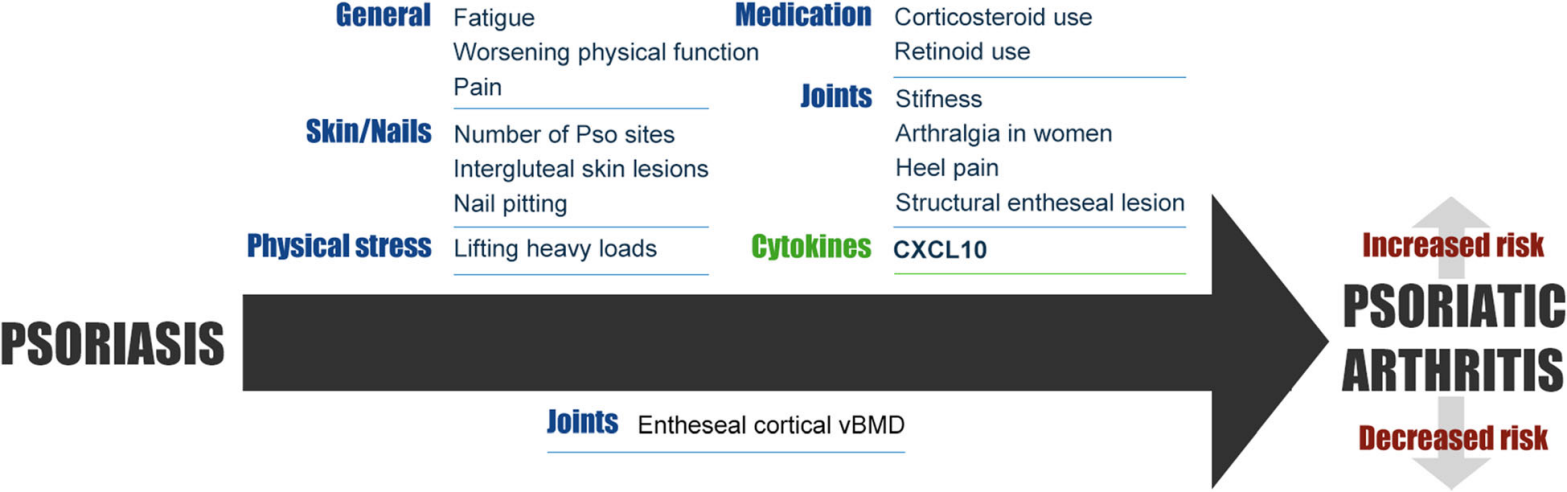
Overview of most promising predictors for the development of psoriatic arthritis in psoriasis patients. Clinical parameters are depicted in blue, laboratory parameters are depicted in green. The strongest evidence is available for the predictive value of CXCL10, this is depicted in bold. CXCL = C-X-C motif ligand; Pso = psoriasis; vBMD = volumetric bone mineral density

Qualitative best evidence synthesis is depicted separately for clinical, laboratory, and genetic studies in Tables 1, 2 and 3. With respect to *predictive* markers for PsA in Pso, we report the markers for which there was at least a moderate level of evidence, or which were investigated in more than one study. With respect to markers associated with the *presence* of PsA in Pso, we report only the markers which were investigated in more than one study. An overview of the most promising findings is also shown in Fig. 2.

### Clinical markers

#### Strong level of evidence

Strong evidence was available for 13 of the 51 investigated clinical markers. All these markers showed no association with the development of PsA in Pso patients. These markers included the following: diabetes [18, 19], diarrhea [18, 20], psoriatic nail lesion [18, 19, 25, 27], menopause [18–20], oral contraceptives [19, 20], alcohol consumption [18–21, 28–30], past smoking [18, 20, 28, 29, 31, 32], methotrexate use [18, 19], age [20, 22, 27, 29], a patient reported family history of PsA [18, 20, 27], female sex [20, 22, 27, 28], trauma [19–21, 35], and psychological distress [22, 23]. There was no strong evidence available for clinical markers that had a positive or negative (i.e., protective) association with the development of PsA.

#### Moderate level of evidence

Moderate evidence was available for 20 of 51 clinical markers. Only six of them were investigated in more than one study. All of these markers showed no association with the development of PsA in Pso. These markers included the following: fertility treatment [20, 21], hormone replacement therapy [20, 21], influenza vaccination [20, 21], tetanus vaccination [20, 21], change in work status [20, 21], and death of a family member [20, 21].

Moderate evidence of a positive association was available for 13 clinical markers. These included the following: uveitis [18], (worsening) fatigue [22], (worsening) function [22], (worsening) pain [22], (worsening) stiffness [22], arthralgia in women [22], heel pain [22], structural entheseal lesions [26], intergluteal skin lesion [25], nail pitting [18], corticosteroid use [19], retinoid use [18], and lifting heavy loads [20].

Moderate evidence of a negative association was available for 1 marker: entheseal cortical volumetric bone mineral density (vBMD) [26].

#### Conflicting evidence

Conflicting evidence was available for 13 of 51 clinical markers. These markers included several disease characteristics: younger age at Pso onset [23–25], longer duration of Pso [27, 28], presence of scalp lesions [25, 27], more severe Pso [18, 20, 22, 25, 27, 28], and higher BMI [18, 22, 27, 29, 33, 34]. Conflicting evidence was also found for infection requiring antibiotics [18, 20], pregnancy [19–21], current smoking [18, 20, 28, 29, 31, 32], rubella vaccination [20, 21], university or high school level of education [18, 20], anxiety/depression [18, 20, 21, 36], and moving to a new home [20, 21].

### Laboratory markers

#### Strong level of evidence

Strong evidence was available for nine of 137 investigated laboratory markers. CXCL10 (C-X-C motif ligand 10) was the only laboratory marker which showed a positive association with the development of PsA in Pso patients. It was also the only laboratory marker studied in a longitudinal design.

Four markers showed a strong level of evidence for a positive association with the presence of PsA in Pso: a higher level of matrix metalloproteinase 3 (MMP3) [49, 51, 52, 54], a higher level of osteoprotegerin (OPG) [49–53, 55], a higher level of interleukin 6 (IL-6) [61–64], and a higher level of C-reactive protein (CRP) [27, 43, 44, 47, 49, 53, 54, 56, 62, 64, 66, 70–75, 124, 130].

Five markers showed a strong level of evidence for no association with PsA in Pso: vitamin D [42–45, 130], serum glucose [42, 62, 71, 76, 77, 130], serum triglycerides [42, 46, 62, 71, 72, 76, 77], serum high-density lipoprotein (HDL) [42, 62, 71, 72, 77], and serum low-density lipoprotein (LDL) [42, 50, 51, 53, 55, 62, 71, 72, 76, 130].

#### Moderate level of evidence

Moderate evidence was available for 56 of 137 investigated laboratory markers. Fourteen of these 56 have been investigated in more than one study.

Of those 14 markers, six showed a positive association with the presence of PsA in Pso: the presence of anti-citrullinated protein antibodies (ACPA) [37–40], a higher level of IL-34 [56, 66], a higher level of tumor necrosis factor alpha (TNFα) [56, 64], a higher mean platelet volume (MPV) [68, 69], a higher LDL:HDL ratio [62, 64, 71, 76], and the presence of microRNA miR-146a-50 [78, 79].

Only one of the 14 markers which were investigated more than once showed moderate evidence of a negative association with the presence of PsA in Pso: a lower ratio of OPG to receptor activator of nuclear factor kappa-B ligand (RANKL) was associated with the presence of PsA in Pso [50, 56].

There was moderate evidence for no association for seven laboratory markers: serum alkalic phosphate [43, 47, 48], serum calcium [47, 48], serum cartilage oligomeric matrix protein (COMP) [49, 50], serum phosphate [43, 47], serum collagen type I C-telopeptide (CTx) [47, 51], serum very low-density lipoprotein (VLDL) [62, 76], and serum creatinine [43, 53].

#### Conflicting evidence

Conflicting evidence was available for 14 of 137 laboratory markers: markers of bone metabolism (Dickkopf (DKK1) [52, 53]; RANK-L [49–51, 53, 56, 57]), markers of lipid metabolism (serum leptin [64, 71]; total serum cholesterol [42, 62, 71, 76, 77]; total cholesterol: HDL ratio [42, 76]; serum triglycerides [42, 71, 72, 76, 77, 130]), inflammation markers (erythrocyte sedimentation rate (ESR) [43, 44, 47, 56, 62, 66, 70, 74, 75], cell numbers (platelet count [68, 70]; white blood cell count [70, 130]), cell phenotype (IL-17 secretion [58, 59]), cytokine levels (IL-12/23 p40 [49, 56]; macrophage colony-stimulating factor (M-CSF) [52, 53]), uric acid [77, 86–88], and antibodies against LL-37 [82, 95].

### Genetic markers

#### Strong level of evidence

There were no genetic markers which reached a strong level of evidence for a positive, negative, or no association with the presence of PsA.

#### Moderate level of evidence

Moderate evidence was available for 30 of 71 investigated genetic markers. Twenty-two of those 31 have been investigated in more than one study.

Of these 22 markers, six showed a positive association with the presence of PsA in Pso: the presence of haplotype B*27-C*01 [97, 99], haplotype B*27-C*02 [97, 99, 100], haplotype B*38-C*12 [97, 99, 100], haplotype B*39:01-C*12 [97, 100], the presence of HLA-B*27 [97, 99, 100, 102–105], and the presence of the single nucleotide polymorphism (SNP) rs1800925 in the *IL13* gene [116, 117].

Moderate evidence of a negative association was available for three markers: the presence of haplotype B*57-C*06 [97, 99], the presence of HLA-C*06 [97, 99–105, 107–109], and the presence of the SNP rs2082412 in the *IL12B* gene [111, 135].

There was moderate evidence for no association for 13 genetic markers: the presence of HLA-B*57 [99, 100, 102, 104], HLA-C*01 [97, 100, 102], HLA-DRB1*03 [101, 102], the presence of the SNP rs397211 of *IL1RN* [111, 135], the presence of the SNP’s rs3212227 [109, 115] and rs6887695 in the *IL12B* gene [109, 115], the presence of the SNP rs2066807 in *IL23A* [111, 135], the presence of the SNP rs11209026 in *IL23R* [109, 115], the presence of the SNP rs610604 in *TNFAIP3* (TNF alpha-induced protein 3) [111, 135], the presence of the SNP rs17728338 in *TNIP* (TNFAIP3-interacting protein) [111, 135], the presence of the SNP rs1076160 in *TSC1* (tuberous sclerosis 1) [111, 135], and the presence of TNFa-238 [109, 122] and TNFa-308 [109, 122].

#### Conflicting evidence

Conflicting evidence was found for 17 of 71 genetic markers, of which 14 were investigated in more than one study. These were the presence of HLA-B*08 [97, 99, 100, 102, 103], HLA-B*13 [101, 102, 104], HLA-B*18 [97, 100], HLA-B*37 [97, 102], HLA-B*38 [97, 99–101, 104], HLA-B*39 [97, 100], HLA-C*02 [97, 99, 100, 102], HLA-C*07 [99, 100, 102], HLA-C*12 [99, 100], HLA-DQB1*02 [101, 102], the presence of glutamic acid (Glu) at HLA-B amino acid position 45 [102, 103, 106], the presence of arginine (Arg) at HLA-B amino position 97 [102, 103], the presence of SNP rs20541 in the *IL13* gene [111, 117, 135], and the presence of SNP rs2201841 in the *IL23R* gene [111, 135].

## Discussion

In this review, we summarized the available evidence for possible markers for the onset or presence of PsA in a Pso patient population in a systematic way. Thereby, we provide an update and addition to a recent narrative review regarding this subject by Scher et al. [10]. When looking at clinical markers, we found only strong evidence for markers which were *not* associated with the development of PsA. Regarding laboratory markers, there was strong evidence for the predictive value of (a change in) CXCL10 serum titers [27, 60]. There was also strong evidence for the association with (but not prediction of) PsA of several markers related to bone metabolism [49–55] and inflammation [27, 43, 44, 47, 49, 53, 54, 56, 58, 61–64, 66, 70–75, 130]. With respect to genetic markers, we found no markers which reached a strong level of evidence for the association with PsA.

In line with previous beliefs on possible clinical risk factors [10, 140], we found moderate evidence for a positive association of gluteal fold lesions [25] and nail pitting for the onset of PsA [18]. However, for nail involvement in general (e.g., distal onycholysis, oil drop phenomenon and crumbling), there was strong evidence of no association [18, 19, 25, 27]. Therefore, this relationship seemed to be restricted to this specific nail feature.

Notably, we found conflicting evidence for the predictive value of obesity [18, 20, 22, 27, 29, 33, 34] and psoriasis severity [18, 20, 22, 25, 27, 28] for the development of PsA in Pso patients. These studies may also be prone to bias because patients with severe Pso differ from patients with mild Pso in several aspects. For instance, when looking at Pso severity in particular, one can argue that more severe skin involvement is treated more intensively, thereby possibly suppressing concomitant arthritis. These kinds of bias may be the reason why these frequently reported markers reach conflicting evidence when all the studies are taking into account in a systematic way.

When looking at BMI at one unspecified timepoint, this marker shows conflicting evidence for a relationship with the development of PsA. In three out of five high/fair quality studies, there was no association [18, 22, 27], while two out of five showed a positive association [29, 33]. Even when taking into account that the before mentioned three studies are performed in a partially overlapping cohort, this marker does not reach the 75% agreement level we consider necessary for a conclusive result. Therefore, BMI at any unspecified timepoint may not be specific enough for prediction of PsA. Interestingly, more specified markers of weight and body composure (e.g., recent weight gain, BMI at younger age or abdominal adiposity) showed a positive association with the development of PsA in Pso but were only investigated in one study of poor quality [34]. Increasing the evidence in a more detailed way may be more valid and relevant.

The association of trauma and psoriatic arthritis was theorized to be due to a deep Koebner phenomenon [140]. This phenomenon is comparable to the well-known Koebner phenomenon in the skin, where trauma can cause the appearance of new skin lesions. The theory on the deep Koebner phenomenon is based on a study of Thorarensen et al., who used diagnostic codes to establish two comparable cohorts (Pso with and without PsA) [35]. However, when forming cohorts in this way, there is a higher risk of misclassification in either cohort. This study is in disagreement with two other papers with higher diagnostic certainty [19, 20]. Therefore, we concluded that there is currently strong evidence that physical trauma is *not* associated with a higher rate of PsA in Pso patients.

The relationship between smoking and PsA development has been described previously as the “smoking paradox” [31]. This entails the fact that smoking appears to be a risk factor for PsA when looking at the general population, but this association disappears when only looking at psoriasis patients. This paradox may be explained by collider bias: bias resulting from correcting for a variable which is a common effect of the exposure and outcome [10]. In our review, we found conflicting evidence for an effect of (current) smoking [18, 20, 28, 29, 31, 32]. However, due to this collider bias, it is hard to determine if smoking leads to additional risk for the development of PsA in a Pso population, unrelated to its effect on the development of Pso. Studies focusing on a change in smoking status after the development of Pso may shed a light on this enigma, as suggested by Nguyen [31].

With regard to laboratory markers, only CXCL10 was studied longitudinally. This cytokine was described in two good/fair quality studies; both found an association between CXCL10 and PsA. Pso patients who developed PsA had a higher CXCL10 serum level at baseline [27]. It was also shown that during the evolution to arthritis the serum level of CXCL10 diminished: a larger negative change was associated with a higher risk of PsA [60]. The reason why CXCL10 levels decreased towards the development of PsA is still unknown. One hypothesis could be that the psoriasis patient group with a high level of CXCL10 is more prone to develop arthritis due to its chemoattractant properties on CXCR3^+^ CD4^+^ and CD8^+^ T cells [141]. In the evolution towards clinical manifest PsA, locally produced CXCL10 might get depleted by these infiltrating and locally expanding inflammatory cells, subsequently lowering circulating CXCL10 levels over time. However, since these two studies were published by the same research group, results may be based on (partially) overlapping patient groups. Therefore, the predicting value of CXCL10 should be interpreted cautiously.

With regard to cross-sectional studies, and markers that may indicate the presence of PsA in Pso patients, we found strong evidence for a positive association with PsA in Pso for markers of inflammation and bone. CRP is a well-known, widely used inflammatory marker. We found strong evidence that the CRP level in PsA patients was higher than in patients with Pso only [27, 43, 44, 47, 49, 53, 54, 56, 64, 66, 70–75, 124, 130]. We argue that the co-appearance of joint inflammation is responsible for this observation. However, we found no articles which studied the level of CRP *before* the start of PsA in Pso. Therefore, it is unknown whether it can be used as a predictive marker. Also, a clear CRP cutoff value for the *presence* of PsA (and therefore, specificity and sensitivity) is lacking.

Other markers for which strong evidence of a positive association with the development of PsA in Pso exist were IL-6, MMP3, and OPG. IL-6 is widely regarded as a marker for systematic inflammation and an important contributor to the production of CRP by the liver. MMP3 and OPG are associated with bone metabolism; one of the hallmark signs of PsA is new bone formation [142]. Also, untreated arthritis can lead to irreversible erosions [4]. Therefore, it is not surprising that MMP and OPG showed an association with the presence of PsA in our review. In line with CRP, the predictive value of these markers is unknown, because longitudinal studies are not performed yet.

Laboratory markers for cardiovascular disease are studied extensively in psoriatic disease [42, 46, 62, 64, 71, 72, 76, 77, 130]. From these findings, we can conclude with strong evidence that these levels do not differ between psoriasis patients with and without arthritis. This is in contrast to a recent review which showed that the prevalence of cardiovascular comorbidities is higher in patients with PsA when compared to Pso [143]. This suggests that there are additional factors (e.g., systemic inflammation) that play a role in cardiovascular morbidity in PsA.

With respect to genetic markers, we focus here on the most important HLA-markers for Pso and PsA, and the IL-12 – IL-23 – IL-17 axis. The most important genetic marker for psoriasis is HLA-C*06, also known as PSOR1 [144]. This marker is responsible for up to 50% of Pso heritability in the healthy population. It is associated with type-I (early onset) psoriasis, as well as a guttate phenotype [145]. Interestingly, our review shows that, when looking within the population of Pso patients, patients with the HLA-C*06 marker were less likely to also have PsA. Despite multiple studies investigating this marker, high-quality studies are needed to confirm the robustness of the negative relationship between HLA-C*06 and the onset of PsA.

We found a moderate level of evidence for the presence of concomitant PsA in Pso for HLA-B*27, known for its high prevalence (90%) in ankylosing spondylitis (AS) [146]. In other diseases of the spondyloarthritis spectrum, the presence of HLA-B*27 is still higher than in the general population, but less than in AS. Our review showed that the presence of HLA-B*27 was higher in the Pso patients who developed arthritis than in the Pso patients who did not. This could indicate that HLA-B*27 may be able to differentiate between Pso patients who do or do not have PsA, which is also considered a part of the spondyloarthritis spectrum.

When looking at the IL-17/IL-23 axis from a genetic viewpoint, there was moderate evidence that there are no SNPs in the *IL23* gene for which the presence differs significantly between PsA and Pso patients [109, 111, 114, 115, 147]. We found limited evidence that the presence of rs79877597 in the *IL17* gene was more common in PsA versus Pso patients [118]. With regard to the common IL-12/IL-23 pathway, there was moderate evidence regarding several SNPs in the *IL12* gene [148]. We found that the presence of one SNP in *IL12* (rs2082412) was lower in PsA versus Pso patients, while other SNPs in this gene showed no difference [109, 111, 114, 115]. While the IL-17/IL-23 axis may be important for the development of psoriatic disease in the general population, these results may indicate that it is of limited importance in the development of PsA in Pso.

The strengths of this study include the extensiveness and systematic way of the search with respect to markers for PsA in patient cohorts with Pso, subsequentially providing a comprehensive overview of the available evidence. Also, the intertwining of clinical, laboratory, and genetic markers in a systematic way is unique. By conducting a best evidence synthesis, taking the study quality into account, we made a qualitative overview of the extensive data.

The limitations of this systematic review are mostly due to the limitations of the included studies. Since there were (almost) no prospective/longitudinal studies looking at genetic and laboratory markers, we could only summarize the level of evidence with regard to the relationship between laboratory and genetic markers with the *presence* of PsA in patients with Pso (i.e., only one predictive factor could be identified). The level of evidence was limited by a paucity of high or fair quality studies. Mostly, this was because of a lack of appropriate definition of patient and control groups, in addition to not adjusting for possible confounders.

## Conclusion

This comprehensive systematic review on clinical, laboratory, and genetic markers for PsA in patients with Pso revealed that a useful set of markers is not established yet. There were no clinical or genetic markers with strong evidence which could predict the development of PsA in Pso cohorts. There was strong evidence that laboratory markers related to bone metabolism and inflammation were associated with the *presence* of PsA. Promising is CXCL10, which reached a strong level of evidence for predicting development of PsA in a Pso population [27, 60]. The importance of timely detecting PsA in a Pso population, and finding more (bio)markers contributing to early detection, remains high.

## Supplementary Information

**Additional file 1: Supplementary table 1.** Search strategy.

**Additional file 2: Supplementary table 2.** Characteristics of included studies (*n* = 119).

**Additional file 3: Supplementary table 3.** Statistical significance and effect sizes of clinical markers.

**Additional file 4: Supplementary table 4.** Statistical significance and effect sizes of laboratory markers.

**Additional file 5: Supplementary table 5.** Statistical significance and effect sizes of genetic markers.

**Additional file 6: Supplementary table 6.** Quality assessment of cohort studies.

**Additional file 7: Supplementary table 7.** Quality assessment of case control studies.

## Data Availability

The data underlying this article will be shared on reasonable request to the corresponding author.

## Abbreviations

ACPA: Anti-citrullinated protein antibodies
Arg: Arginine
AS: Ankylosing spondylitis
BES: Best evidence synthesis
BMI: Body mass index
COMP: Cartilage oligomeric matrix protein
CRP: C-reactive protein
CTx: Collagen type I C-telopeptide
CXCL: C-X-C motif ligand
DKK1: Dickkopf 1
ESR: Erythrocyte sedimentation rate
Glu: Glutamic acid
HDL: High-density lipoprotein
HLA: Human leukocyte antigen
IL: Interleukin
LDL: Low-density lipoprotein
M-CSF: Macrophage colony-stimulating factor
MMP3: Metalloproteinase 3
MPV: Mean platelet volume
OPG: Osteoprotegrin
PsA: Psoriatic arthritis
Pso: Psoriasis
RANKL: Receptor activator of nuclear factor kappa-B ligand
SNP: Single nucleotide polymorphism
TNF: Tumor necrosis factor
TNFAIP: TNF alpha-induced protein
TNIP: TNFAIP3-interacting protein
TSC1: Tuberous sclerosis 1
vBMD: Volumetric bone mineral density
VLDL: Very low-density lipoprotein

## References

[1] Mease PJ, Gladman DD, Papp KA, Khraishi MM, Thaci D, Behrens F (2013). Prevalence of rheumatologist-diagnosed psoriatic arthritis in patients with psoriasis in European/North American dermatology clinics. J Am Acad Dermatol..

[2] Tillett W, Charlton R, Nightingale A, Snowball J, Green A, Smith C (2017). Interval between onset of psoriasis and psoriatic arthritis comparing the UK Clinical Practice Research Datalink with a hospital-based cohort. Rheumatology (Oxford)..

[3] Kane D, Stafford L, Bresnihan B, FitzGerald O (2003). A prospective, clinical and radiological study of early psoriatic arthritis: an early synovitis clinic experience. Rheumatology (Oxford)..

[4] Haroon M, Gallagher P, FitzGerald O (2015). Diagnostic delay of more than 6 months contributes to poor radiographic and functional outcome in psoriatic arthritis. Ann Rheum Dis..

[5] Coates LC, Moverley AR, McParland L, Brown S, Navarro-Coy N, O'Dwyer JL (2015). Effect of tight control of inflammation in early psoriatic arthritis (TICOPA): a UK multicentre, open-label, randomised controlled trial. Lancet..

[6] Ibrahim GH, Buch MH, Lawson C, Waxman R, Helliwell PS (2009). Evaluation of an existing screening tool for psoriatic arthritis in people with psoriasis and the development of a new instrument: the Psoriasis Epidemiology Screening Tool (PEST) questionnaire. Clin Exp Rheumatol..

[7] Coates LC, Aslam T, Al BF, Burden AD, Burden-Teh E, Caperon AR (2013). Comparison of three screening tools to detect psoriatic arthritis in patients with psoriasis (CONTEST study). Br J Dermatol..

[8] Chimenti MS, Triggianese P, De Martino E, Conigliaro P, Fonti GL, Sunzini F (2019). An update on pathogenesis of psoriatic arthritis and potential therapeutic targets. Expert Rev Clin Immunol..

[9] Solmaz D, Eder L, Aydin SZ (2018). Update on the epidemiology, risk factors, and disease outcomes of psoriatic arthritis. Best Pract Res Clin Rheumatol..

[10] Scher JU, Ogdie A, Merola JF, Ritchlin C (2019). Preventing psoriatic arthritis: focusing on patients with psoriasis at increased risk of transition. Nat Rev Rheumatol..

[11] Rahmati S, Tsoi L, O'Rielly D, Chandran V, Rahman P (2020). Complexities in genetics of psoriatic arthritis. Curr Rheumatol Rep..

[12] Villanova F, Di Meglio P, Nestle FO (2013). Biomarkers in psoriasis and psoriatic arthritis. Ann Rheum Dis.

[13] Villanova F, Di MP, Nestle FO (2013). Biomarkers in psoriasis and psoriatic arthritis. Ann Rheum Dis.

[14] Generali E, Scire CA, Favalli EG, Selmi C (2016). Biomarkers in psoriatic arthritis: a systematic literature review. Expert Rev Clin Immunol..

[15] Shamseer L, Moher D, Clarke M, Ghersi D, Liberati A, Petticrew M (2015). Preferred reporting items for systematic review and meta-analysis protocols (PRISMA-P) 2015: elaboration and explanation. BMJ..

[16] Wells GA, Shea B, O'Connell D, Peterson J, Welch V, Losos M, et al. The Newcastle-Ottawa Scale (NOS) for assessing the quality of nonrandomised studies in meta-analysis. http://www.ohri.ca/programs/clinical_epidemiology/oxford.asp.

[17] Sackett DL (2000). Evidence-based medicine: how to practice and reach EBM. 2nd edition ed.

[18] Eder L, Haddad A, Rosen CF, Lee KA, Chandran V, Cook R (2016). The incidence and risk factors for psoriatic arthritis in patients with psoriasis: a prospective cohort Study. Arthritis Rheumatol..

[19] Thumboo J, Uramoto K, Shbeeb MI, O'Fallon WM, Crowson CS, Gibson LE (2002). Risk factors for the development of psoriatic arthritis: a population based nested case control study. J Rheumatol..

[20] Eder L, Law T, Chandran V, Shanmugarajah S, Shen H, Rosen CF (2011). Association between environmental factors and onset of psoriatic arthritis in patients with psoriasis. Arthritis Care Res (Hoboken )..

[21] Pattison E, Harrison BJ, Griffiths CE, Silman AJ, Bruce IN (2008). Environmental risk factors for the development of psoriatic arthritis: results from a case-control study. Ann Rheum Dis..

[22] Eder L, Polachek A, Rosen CF, Chandran V, Cook R, Gladman DD (2017). The development of psoriatic arthritis in patients with psoriasis is preceded by a period of nonspecific musculoskeletal symptoms: a prospective cohort study. Arthritis Rheumatol..

[23] Egeberg A, Skov L, Zachariae C, Gislason GH, Thyssen JP, Mallbris L (2018). Duration of psoriatic skin disease as risk factor for subsequent onset of psoriatic arthritis. Acta Derm Venereol..

[24] Soltani-Arabshahi R, Wong B, Feng BJ, Goldgar DE, Duffin KC, Krueger GG (2010). Obesity in early adulthood as a risk factor for psoriatic arthritis. Arch Dermatol..

[25] Wilson FC, Icen M, Crowson CS, McEvoy MT, Gabriel SE, Kremers HM (2009). Incidence and clinical predictors of psoriatic arthritis in patients with psoriasis: a population-based study. Arthritis Rheum..

[26] Simon D, Tascilar K, Kleyer A, Bayat S, Kampylafka E, Sokolova M, et al. Structural entheseal lesions in patients with psoriasis are associated with an increased risk of progression to psoriatic arthritis. Arthritis Rheumatol. 2020. 10.1002/art.41239. 10.1002/art.41239 32103639

[27] Abji F, Pollock RA, Liang K, Chandran V, Gladman DD (2016). Brief Report: CXCL10 is a possible biomarker for the development of psoriatic arthritis among patients with psoriasis. Arthritis Rheumatol..

[28] Eder L, Shanmugarajah S, Thavaneswaran A, Chandran V, Rosen CF, Cook RJ (2012). The association between smoking and the development of psoriatic arthritis among psoriasis patients. Ann Rheum Dis..

[29] Green A, Shaddick G, Charlton R, Snowball J, Nightingale A, Smith C, Tillett W, McHugh N, on behalf of the PROMPT study group (2020). Modifiable risk factors and the development of psoriatic arthritis in people with psoriasis. Br J Dermatol..

[30] Wu S, Cho E, Li WQ, Han J, Qureshi AA (2015). Alcohol intake and risk of incident psoriatic arthritis in women. J Rheumatol..

[31] Nguyen UDT, Zhang Y, Lu N, Louie-Gao Q, Niu J, Ogdie A (2018). Smoking paradox in the development of psoriatic arthritis among patients with psoriasis: a population-based study. Ann Rheum Dis..

[32] Li W, Han J, Qureshi AA (2012). Smoking and risk of incident psoriatic arthritis in US women. Ann Rheum Dis..

[33] Love TJ, Zhu Y, Zhang Y, Wall-Burns L, Ogdie A, Gelfand JM (2012). Obesity and the risk of psoriatic arthritis: a population-based study. Ann Rheum Dis..

[34] Li W, Han J, Qureshi AA (2012). Obesity and risk of incident psoriatic arthritis in US women. Ann Rheum Dis..

[35] Thorarensen SM, Lu N, Ogdie A, Gelfand JM, Choi HK, Love TJ (2017). Physical trauma recorded in primary care is associated with the onset of psoriatic arthritis among patients with psoriasis. Ann Rheum Dis..

[36] Lewinson RT, Vallerand IA, Lowerison MW, Parsons LM, Frolkis AD, Kaplan GG, Bulloch AGM, Swain MG, Patten SB, Barnabe C (2017). Depression is associated with an increased risk of psoriatic arthritis among patients with psoriasis: a population-based study. J Invest Dermatol..

[37] Abdel Fattah NS, Hassan HE, Galal ZA, El Okdael SE (2009). Assessment of anti-cyclic citrullinated peptide in psoriatic arthritis. BMC Res Notes.

[38] Candia L, Marquez J, Gonzalez C, Santos AM, Londono J, Valle R (2006). Low frequency of anticyclic citrullinated peptide antibodies in psoriatic arthritis but not in cutaneous psoriasis. J Clin Rheumatol..

[39] Alenius GM, Berglin E, Rantapaa DS (2006). Antibodies against cyclic citrullinated peptide (CCP) in psoriatic patients with or without joint inflammation. Ann Rheum Dis..

[40] Shibata S, Tada Y, Komine M, Hattori N, Osame S, Kanda N (2009). Anti-cyclic citrullinated peptide antibodies and IL-23p19 in psoriatic arthritis. J Dermatol Sci..

[41] Dalmady S, Kiss M, Kepiro L, Kovacs L, Sonkodi G, Kemeny L (2013). Higher levels of autoantibodies targeting mutated citrullinated vimentin in patients with psoriatic arthritis than in patients with psoriasis vulgaris. Clin Dev Immunol..

[42] Orgaz-Molina J, Magro-Checa C, Rosales-Alexander JL, Arrabal-Polo MA, Buendia-Eisman A, Raya-Alvarez E (2013). Association of 25-hydroxyvitamin D serum levels and metabolic parameters in psoriatic patients with and without arthritis. J Am Acad Dermatol..

[43] Sag MS, Sag S, Tekeoglu I, Solak B, Kamanli A, Nas K (2018). Comparison of 25-hidroksi vitamin D serum concentrations in patients with psoriasis and psoriatic arthritis. J Back Musculoskelet Rehabil..

[44] Esawy MM, Makram WK, Albalat W, Shabana MA (2020). Plasma gelsolin levels in patients with psoriatic arthritis: a possible novel marker. Clin Rheumatol..

[45] Gisondi P, Rossini M, Di Cesare A, Idolazzi L, Farina S, Beltrami G (2012). Vitamin D status in patients with chronic plaque psoriasis. Br J Dermatol..

[46] Mysliwiec H, Baran A, Harasim-Symbor E, Choromanska B, Mysliwiec P, Milewska AJ (2017). Increase in circulating sphingosine-1-phosphate and decrease in ceramide levels in psoriatic patients. Arch Dermatol Res..

[47] Borman P, Babaoglu S, Gur G, Bingol S, Bodur H (2008). Bone mineral density and bone turnover in patients with psoriatic arthritis. Clin Rheumatol..

[48] Hein G, Abendroth K, Muller A, Wessel G (1991). Studies on psoriatic osteopathy. Clin Rheumatol..

[49] Chandran V, Cook RJ, Edwin J, Shen H, Pellett FJ, Shanmugarajah S (2010). Soluble biomarkers differentiate patients with psoriatic arthritis from those with psoriasis without arthritis. Rheumatology (Oxford)..

[50] Bartosinska J, Michalak-Stoma A, Juszkiewicz-Borowiec M, Kowal M, Chodorowska G (2015). The assessment of selected bone and cartilage biomarkers in psoriatic patients from Poland. Mediators Inflamm..

[51] Diani M, Perego S, Sansoni V, Bertino L, Gomarasca M, Faraldi M, et al. Differences in osteoimmunological biomarkers predictive of psoriatic arthritis among a large Italian cohort of psoriatic patients. Int J Mol Sci. 2019;20(22):5617. 10.3390/ijms20225617. 10.3390/ijms20225617 PMC6888436 31717649

[52] Jadon DR, Sengupta R, Nightingale A, Lu H, Dunphy J, Green A (2017). Serum bone-turnover biomarkers are associated with the occurrence of peripheral and axial arthritis in psoriatic disease: a prospective cross-sectional comparative study. Arthritis Res Ther..

[53] Dalbeth N, Pool B, Smith T, Callon KE, Lobo M, Taylor WJ (2010). Circulating mediators of bone remodeling in psoriatic arthritis: implications for disordered osteoclastogenesis and bone erosion. Arthritis Res Ther..

[54] Cretu D, Gao L, Liang K, Soosaipillai A, Diamandis EP, Chandran V (2018). Differentiating psoriatic arthritis from psoriasis without psoriatic arthritis using novel serum biomarkers. Arthritis Care Res (Hoboken )..

[55] Attia EA, Khafagy A, Abdel-Raheem S, Fathi S, Saad AA (2011). Assessment of osteoporosis in psoriasis with and without arthritis: correlation with disease severity. Int J Dermatol..

[56] Li J, Liu L, Rui W, Li X, Xuan D, Zheng S (2017). New interleukins in psoriasis and psoriatic arthritis patients: the possible roles of interleukin-33 to interleukin-38 in disease activities and bone erosions. Dermatology..

[57] Amin TE, ElFar NN, Ghaly NR, Hekal MM, Hassan AM, Elsaadany HM (2016). Serum level of receptor activator of nuclear factor kappa-B ligand in patients with psoriasis. Int J Dermatol..

[58] Bos F, Capsoni F, Molteni S, Raeli L, Diani M, Altomare A (2014). Differential expression of interleukin-2 by anti-CD3-stimulated peripheral blood mononuclear cells in patients with psoriatic arthritis and patients with cutaneous psoriasis. Clin Exp Dermatol..

[59] Benham H, Norris P, Goodall J, Wechalekar MD, FitzGerald O, Szentpetery A (2013). Th17 and Th22 cells in psoriatic arthritis and psoriasis. Arthritis Res Ther..

[60] Abji F, Lee KA, Pollock RA, Machhar R, Cook RJ, Chandran V, et al. Declining levels of serum chemokine (C-X-C motif) ligand 10 over time are associated with new onset of psoriatic arthritis in patients with psoriasis: a new biomarker? Br J Dermatol. 2020;183(5):920–7. 10.1111/bjd.18940. 10.1111/bjd.18940 32037514

[61] Spadaro A, Taccari E, Riccieri V, Sensi F, Sili Scavalli A, Zoppini A (1996). Interleukin-6 and soluble interleukin-2-receptor in psoriatic arthritis: correlations with clinical and laboratory parameters. Clin Exp Rheumatol..

[62] Pietrzak A, Chabros P, Grywalska E, Pietrzak D, Kandzierski G, Wawrzycki BO (2020). Serum concentration of interleukin 6 is related to inflammation and dyslipidemia in patients with psoriasis. Postepy Dermatol Alergol..

[63] Alenius GM, Eriksson C, Rantapaa DS (2009). Interleukin-6 and soluble interleukin-2 receptor alpha-markers of inflammation in patients with psoriatic arthritis?. Clin Exp Rheumatol..

[64] Johnson CM, Fitch K, Merola JF, Han J, Qureshi AA, Li WQ (2019). Plasma levels of tumour necrosis factor-alpha and adiponectin can differentiate patients with psoriatic arthritis from those with psoriasis. Br J Dermatol..

[65] Pirowska M, Obtulowicz A, Lipko-Godlewska S, Gozdzialska A, Podolec K, Wojas-Pelc A (2018). The level of proinflammatory cytokines: interleukins 12, 23, 17 and tumor necrosis factor alpha in patients with metabolic syndrome accompanying severe psoriasis and psoriatic arthritis. Postepy Dermatol Alergol..

[66] Ausavarungnirun R, Intarasupht J, Nakakes A, Rojanametin K (2017). Nail abnormalities, quality of life and serum inflammatory marker in psoriatic arthritis compare to psoriasis without arthritis. J Med Assoc Thailand..

[67] Diani M, Casciano F, Marongiu L, Longhi M, Altomare A, Pigatto PD, Secchiero P, Gambari R, Banfi G, Manfredi AA, Altomare G, Granucci F, Reali E (2019). Increased frequency of activated CD8(+) T cell effectors in patients with psoriatic arthritis. Sci Rep..

[68] Canpolat F, Akpinar H, Eskioglu F (2010). Mean platelet volume in psoriasis and psoriatic arthritis. Clin Rheumatol..

[69] Kilic S, Resorlu H, Isik S, Oymak S, Akbal A, Hiz MM (2017). Association between mean platelet volume and disease severity in patients with psoriasis and psoriatic arthritis. Postepy Dermatol Alergol..

[70] Kim DS, Shin D, Lee MS, Kim HJ, Kim DY, Kim SM, Lee MG (2016). Assessments of neutrophil to lymphocyte ratio and platelet to lymphocyte ratio in Korean patients with psoriasis vulgaris and psoriatic arthritis. J Dermatol..

[71] Eder L, Jayakar J, Pollock R, Pellett F, Thavaneswaran A, Chandran V (2013). Serum adipokines in patients with psoriatic arthritis and psoriasis alone and their correlation with disease activity. Ann Rheum Dis..

[72] Husni ME, Wilson Tang WH, Lucke M, Chandrasekharan UM, Brennan DM, Hazen SL (2018). Correlation of high-density lipoprotein-associated paraoxonase 1 activity with systemic inflammation, disease activity, and cardiovascular risk factors in psoriatic disease. Arthritis Rheumatol..

[73] Lin YC, Dalal D, Churton S, Brennan DM, Korman NJ, Kim ES (2014). Relationship between metabolic syndrome and carotid intima-media thickness: cross-sectional comparison between psoriasis and psoriatic arthritis. Arthritis Care Res (Hoboken )..

[74] Krajewska-Wlodarczyk M, Owczarczyk-Saczonek A, Placek W, Wojtkiewicz M, Wiktorowicz A, Wojtkiewicz J (2019). Distal interphalangeal joint extensor tendon enthesopathy in patients with nail psoriasis. Sci Rep..

[75] Hur MS, Hong JY, Hong JR, Lee YW, Choe YB, Ahn KJ. Clinical characteristics of psoriatic patients with latent tuberculosis infection. Eur J Dermatol. 2020. 10.1684/ejd.2020.3757. 10.1684/ejd.2020.3757 32301723

[76] Pietrzak A, Chabros P, Grywalska E, Kicinski P, Pietrzak-Franciszkiewicz K, Krasowska D (2019). Serum lipid metabolism in psoriasis and psoriatic arthritis - an update. Arch Med Sci..

[77] Ortolan A, Lorenzin M, Tadiotto G, Russo FP, Oliviero F, Felicetti M (2019). Metabolic syndrome, non-alcoholic fatty liver disease and liver stiffness in psoriatic arthritis and psoriasis patients. Clin Rheumatol..

[78] Pasquali L, Svedbom A, Srivastava A, Rosen E, Lindqvist U, Stahle M (2020). Circulating microRNAs in extracellular vesicles as potential biomarkers for psoriatic arthritis in patients with psoriasis. J Eur Acad Dermatol Venereol..

[79] Lin SH, Ho JC, Li SC, Chen JF, Hsiao CC, Lee CH. MiR-146a-5p Expression in Peripheral CD14(+) Monocytes from Patients with Psoriatic Arthritis Induces Osteoclast Activation, Bone Resorption, and Correlates with Clinical Response. J Clin Med. 2019;8(1):110. 10.3390/jcm8010110. 10.3390/jcm8010110 PMC6352034 30658492

[80] Abji F, Pollock RA, Liang K, Chandran V, Gladman DD (2018). Th17 gene expression in psoriatic arthritis synovial fluid and peripheral blood compared to osteoarthritis and cutaneous psoriasis. Clin Exp Rheumatol..

[81] Pollock RA, Abji F, Liang K, Chandran V, Pellett FJ, Virtanen C, Gladman DD (2015). Gene expression differences between psoriasis patients with and without inflammatory arthritis. J Invest Dermatol..

[82] Frasca L, Palazzo R, Chimenti MS, Alivernini S, Tolusso B, Bui L (2018). Anti-LL37 antibodies are present in psoriatic arthritis (PsA) patients: new biomarkers in PsA. Front Immunol..

[83] De Andrea M, De Santis M, Caneparo V, Generali E, Sirotti S, Isailovic N, et al. Serum IFI16 and anti-IFI16 antibodies in psoriatic arthritis. Clin Exp Immunol. 2020;199(1):88–96. 10.1111/cei.13376. 10.1111/cei.13376 PMC6904656 31571199

[84] Maejima H, Nagashio R, Yanagita K, Hamada Y, Amoh Y, Sato Y, Katsuoka K (2014). Moesin and stress-induced phosphoprotein-1 are possible sero-diagnostic markers of psoriasis. PLoS One..

[85] Husakova M, Lippert J, Stolfa J, Sedova L, Arenberger P, Lacinova Z (2015). Elevated serum prolactin levels as a marker of inflammatory arthritis in psoriasis vulgaris. Biomed Pap Med Fac Univ Palacky Olomouc Czech Repub..

[86] Tsuruta N, Imafuku S, Narisawa Y (2017). Hyperuricemia is an independent risk factor for psoriatic arthritis in psoriatic patients. J Dermatol..

[87] Barbarroja N, Arias-de la Rosa I, Lopez-Medina C, Camacho-Sanchez MDR, Gomez-Garcia I, Velez-Garcia AJ (2019). Cardiovascular risk factors in psoriatic disease: psoriasis versus psoriatic arthritis. Ther Adv Musculoskelet Dis.

[88] Yilmaz E, Tamer E, Artuz F, Kulcu Cakmak S, Kokturk F (2017). Evaluation of serum uric acid levels in psoriasis vulgaris. Turk J Med Sci..

[89] Maejima H, Kobayashi M, Yanagita K, Hamada Y, Nagashio R, Sato Y (2017). Valosin-containing protein is a possible sero-diagnostic marker of psoriatic arthritis. Biomed Res (India)..

[90] Hong X, Jiang S, Marmolejo N, Vangipuram R, Ramos-Rojas E, Yuan Y (2018). Serum vascular endothelial growth factor receptor 3 as a potential biomarker in psoriasis. Exp Dermatol..

[91] Jensen P, Wiell C, Milting K, Poggenborg RP, Ostergaard M, Johansen JS (2013). Plasma YKL-40: a potential biomarker for psoriatic arthritis?. J Eur Acad Dermatol Venereol..

[92] Cretu D, Liang K, Saraon P, Batruch I, Diamandis EP, Chandran V (2015). Quantitative tandem mass-spectrometry of skin tissue reveals putative psoriatic arthritis biomarkers. Clin Proteomics..

[93] Abdelaal NH, Elhefnawy NG, Abdulmonem SR, Sayed S, Saleh NA, Saleh MA (2020). Evaluation of the expression of the stromal cell-derived factor-1 alpha (CXCL 12) in psoriatic patients after treatment with methotrexate. J Cosmet Dermatol..

[94] El-Leithy S, Sherif N, El-Arousy NH, El-Hilaly R, Shakweer MM. Cutaneous immunohistochemical expression of interleukin-23 receptor (IL-23R) in psoriasis and psoriatic arthritis patients: relation to musculoskeletal ultrasound findings. Egypt Rheumatol. 2020;42(4):313–18.

[95] Yuan Y, Qiu J, Lin ZT, Li W, Haley C, Mui UN, Ning J, Tyring SK, Wu T (2019). Identification of novel autoantibodies associated with psoriatic arthritis. Arthritis Rheumatol..

[96] Muto M, Date Y, Ichimiya M, Moriwaki Y, Mori K, Kamikawaji N (1996). Significance of antibodies to streptococcal M protein in psoriatic arthritis and their association with HLA-A*0207. Tissue Antigens..

[97] Winchester R, Minevich G, Steshenko V, Kirby B, Kane D, Greenberg DA (2012). HLA associations reveal genetic heterogeneity in psoriatic arthritis and in the psoriasis phenotype. Arthritis Rheum..

[98] Pollock R, Chandran V, Barrett J, Eder L, Pellett F, Yao C (2011). Differential major histocompatibility complex class I chain-related A allele associations with skin and joint manifestations of psoriatic disease. Tissue Antigens..

[99] Eder L, Chandran V, Pellet F, Shanmugarajah S, Rosen CF, Bull SB, Gladman DD (2012). Human leucocyte antigen risk alleles for psoriatic arthritis among patients with psoriasis. Ann Rheum Dis..

[100] Eder L, Chandran V, Pellett F, Shanmugarajah S, Rosen CF, Bull SB, Gladman DD (2012). Differential human leucocyte allele association between psoriasis and psoriatic arthritis: a family-based association study. Ann Rheum Dis..

[101] Elkayam O, Segal R, Caspi D (2004). Human leukocyte antigen distribution in Israeli patients with psoriatic arthritis. Rheumatol Int..

[102] Aterido A, Canete JD, Tornero J, Ferrandiz C, Pinto JA, Gratacos J, et al. Genetic variation at the glycosaminoglycan metabolism pathway contributes to the risk of psoriatic arthritis but not psoriasis. Ann Rheum Dis. 2019;78(3):e214158. 10.1136/annrheumdis-2018-214158. 10.1136/annrheumdis-2018-214158 30552173

[103] Bowes J, Ashcroft J, Dand N, Jalali-Najafabadi F, Bellou E, Ho P (2017). Cross-phenotype association mapping of the MHC identifies genetic variants that differentiate psoriatic arthritis from psoriasis. Ann Rheum Dis..

[104] Pollock RA, Chandran V, Pellett FJ, Thavaneswaran A, Eder L, Barrett J (2013). The functional MICA-129 polymorphism is associated with skin but not joint manifestations of psoriatic disease independently of HLA-B and HLA-C. Tissue Antigens..

[105] Liao HT, Lin KC, Chang YT, Chen CH, Liang TH, Chen WS (2008). Human leukocyte antigen and clinical and demographic characteristics in psoriatic arthritis and psoriasis in Chinese patients. J Rheumatol..

[106] Okada Y, Han B, Tsoi LC, Stuart PE, Ellinghaus E, Tejasvi T (2014). Fine mapping major histocompatibility complex associations in psoriasis and its clinical subtypes. Am J Hum Genet..

[107] Coto-Segura P, Coto E, Gonzalez-Lara L, Alonso B, Gomez J, Cuesta-Llavona E (2019). Gene variant in the NF-kappaB pathway inhibitor NFKBIA distinguishes patients with psoriatic arthritis within the spectrum of psoriatic disease. Biomed Res Int..

[108] Ho PY, Barton A, Worthington J, Plant D, Griffiths CE, Young HS (2008). Investigating the role of the HLA-Cw*06 and HLA-DRB1 genes in susceptibility to psoriatic arthritis: comparison with psoriasis and undifferentiated inflammatory arthritis. Ann Rheum Dis..

[109] Cabaleiro T, Roman M, Gallo E, Ochoa D, Tudelilla F, Talegon M (2013). Association between psoriasis and polymorphisms in the TNF, IL12B, and IL23R genes in Spanish patients. Eur J Dermatol..

[110] Julia A, Tortosa R, Hernanz JM, Canete JD, Fonseca E, Ferrandiz C (2012). Risk variants for psoriasis vulgaris in a large case-control collection and association with clinical subphenotypes. Hum Mol Genet..

[111] Nair RP, Duffin KC, Helms C, Ding J, Stuart PE, Goldgar D (2009). Genome-wide scan reveals association of psoriasis with IL-23 and NF-kappaB pathways. Nat Genet..

[112] Julia A, Pinto JA, Gratacos J, Queiro R, Ferrandiz C, Fonseca E (2015). A deletion at ADAMTS9-MAGI1 locus is associated with psoriatic arthritis risk. Ann Rheum Dis..

[113] Soto-Sanchez J, Santos-Juanes J, Coto-Segura P, Coto E, Diaz M, Rodriguez I (2010). Genetic variation at the CCR5/CCR2 gene cluster and risk of psoriasis and psoriatic arthritis. Cytokine..

[114] Yang Q, Liu H, Qu L, Fu X, Yu Y, Yu G (2013). Investigation of 20 non-HLA (human leucocyte antigen) psoriasis susceptibility loci in Chinese patients with psoriatic arthritis and psoriasis vulgaris. Br J Dermatol..

[115] Loft ND, Skov L, Rasmussen MK, Gniadecki R, Dam TN, Brandslund I (2018). Genetic polymorphisms associated with psoriasis and development of psoriatic arthritis in patients with psoriasis. PLoS One..

[116] Eder L, Chandran V, Pellett F, Pollock R, Shanmugarajah S, Rosen CF (2011). IL13 gene polymorphism is a marker for psoriatic arthritis among psoriasis patients. Ann Rheum Dis..

[117] Bowes J, Eyre S, Flynn E, Ho P, Salah S, Warren RB, Marzo-Ortega H, Coates L, McManus R, Ryan AW, Kane D, Korendowych E, McHugh N, FitzGerald O, Packham J, Morgan AW, Griffiths CEM, Bruce IN, Worthington J, Barton A (2011). Evidence to support IL-13 as a risk locus for psoriatic arthritis but not psoriasis vulgaris. Ann Rheum Dis..

[118] Batalla A, Coto E, Gonzalez-Lara L, Gonzalez-Fernandez D, Gomez J, Aranguren TF (2015). Association between single nucleotide polymorphisms IL17RA rs4819554 and IL17E rs79877597 and psoriasis in a Spanish cohort. J Dermatol Sci..

[119] Williams F, Meenagh A, Sleator C, Cook D, Fernandez-Vina M, Bowcock AM (2005). Activating killer cell immunoglobulin-like receptor gene KIR2DS1 is associated with psoriatic arthritis. Hum Immunol..

[120] Stuart PE, Nair RP, Tsoi LC, Tejasvi T, Das S, Kang HM (2015). Genome-wide association analysis of psoriatic arthritis and cutaneous psoriasis reveals differences in their genetic architecture. Am J Hum Genet..

[121] Bowes J, Loehr S, Budu-Aggrey A, Uebe S, Bruce IN, Feletar M (2015). PTPN22 is associated with susceptibility to psoriatic arthritis but not psoriasis: evidence for a further PsA-specific risk locus. Ann Rheum Dis..

[122] Isik S, Silan F, Kilic S, Hiz MM, Ogretmen Z, Ozdemir O (2016). 308G/A and 238G/A polymorphisms in the TNF-alpha gene may not contribute to the risk of arthritis among Turkish psoriatic patients. Egyptian Rheumatologist..

[123] Hohler T, Grossmann S, Stradmann-Bellinghausen B, Kaluza W, Reuss E (2002). de VK, et al. Differential association of polymorphisms in the TNFalpha region with psoriatic arthritis but not psoriasis. Ann Rheum Dis..

[124] Bostoen J, Van PL, Brochez L, Mielants H, Lambert J (2014). A cross-sectional study on the prevalence of metabolic syndrome in psoriasis compared to psoriatic arthritis. J Eur Acad Dermatol Venereol..

[125] Calzavara-Pinton PG, Franceschini F, Manera C, Zane C, Prati E, Cretti L (1999). Antiperinuclear factor in psoriatic arthropathy. J Am Acad Dermatol..

[126] Eder L, Jayakar J, Shanmugarajah S, Thavaneswaran A, Pereira D, Chandran V (2013). The burden of carotid artery plaques is higher in patients with psoriatic arthritis compared with those with psoriasis alone. Ann Rheum Dis..

[127] Engin B, Tanakol A, Bulut H, Songur A, Vehid HE, Gokalp E (2020). Changes in serum TNF-like weak inducer of apoptosis (TWEAK) levels and Psoriasis Area Severity Index (PASI) scores in plaque psoriasis patients treated with conventional versus anti-TNF treatments. Int J Dermatol..

[128] Li X, Miao X, Wang H, Wang Y, Li F, Yang Q (2016). Association of serum uric acid levels in psoriasis: a systematic review and meta-analysis. Medicine (Baltimore)..

[129] Mavropoulos A, Varna A, Zafiriou E, Liaskos C, Alexiou I, Roussaki-Schulze A, Vlychou M, Katsiari C, Bogdanos DP, Sakkas LI (2017). IL-10 producing Bregs are impaired in psoriatic arthritis and psoriasis and inversely correlate with IL-17- and IFNgamma-producing T cells. Clin Immunol..

[130] Mysliwiec H, Harasim-Symbor E, Baran A, Szterling-Jaworowska M, Milewska AJ, Chabowski A (2019). Abnormal serum fatty acid profile in psoriatic arthritis. Arch Med Sci..

[131] Farrag DA, Asaad MK, Ghobrial CK (2017). Evaluation of IL-34 in psoriasis and psoriatic arthritis patients: correlation with disease activity and severity. Egyptian Rheumatologist..

[132] Krajewska-Wlodarczyk M, Owczarczyk-Saczonek A, Placek W (2017). Changes in body composition and bone mineral density in postmenopausal women with psoriatic arthritis. Reumatologia..

[133] Bartosinska J, Purkot J, Kowal M, Michalak-Stoma A, Krasowska D, Chodorowska G (2018). The expression of selected molecular markers of immune tolerance in psoriatic patients. Adv Clin Exp Med..

[134] Eiris N, Gonzalez-Lara L, Santos-Juanes J, Queiro R, Coto E, Coto-Segura P (2014). Genetic variation at IL12B, IL23R and IL23A is associated with psoriasis severity, psoriatic arthritis and type 2 diabetes mellitus. J Dermatol Sci..

[135] Yang YW, Kang JH, Lin HC (2012). Increased risk of psoriasis following obstructive sleep apnea: a longitudinal population-based study. Sleep Med..

[136] Pollock RA, Zaman L, Chandran V, Gladman DD (2019). Epigenome-wide analysis of sperm cells identifies IL22 as a possible germ line risk locus for psoriatic arthritis. PLoS One..

[137] Voiculescu VM, Solomon I, Popa A, Draghici CC, Dobre M, Giurcaneanu C (2018). Gene polymorphisms of TNF-238G/A, TNF-308G/A, IL10-1082G/A, TNFAIP3, and MC4R and comorbidity occurrence in a Romanian population with psoriasis. J Med Life..

[138] Yan D, Ahn R, Leslie S, Liao W (2018). Clinical and genetic risk factors associated with psoriatic arthritis among patients with psoriasis. Dermatol Ther (Heidelb)..

[139] Zhao Q, Sun Y, Fu X, Wang Z, Yu G, Yue Z (2019). Identification of a single nucleotide polymorphism in NFKBIA with different effects on psoriatic arthritis and cutaneous psoriasis in China. Acta Derm Venereol..

[140] Ritchlin CT, Colbert RA, Gladman DD (2017). Psoriatic arthritis. N Engl J Med..

[141] Aggarwal A, Agarwal S, Misra R (2007). Chemokine and chemokine receptor analysis reveals elevated interferon-inducible protein-10 (IP)-10/CXCL10 levels and increased number of CCR5+ and CXCR3+ CD4 T cells in synovial fluid of patients with enthesitis-related arthritis (ERA). Clin Exp Immunol..

[142] Taylor W, Gladman D, Helliwell P, Marchesoni A, Mease P, Mielants H (2006). Classification criteria for psoriatic arthritis: development of new criteria from a large international study. Arthritis Rheum..

[143] Puig L. Cardiometabolic comorbidities in psoriasis and psoriatic arthritis. Int J Mol Sci. 2017;19(1):58. 10.3390/ijms19010058. 10.3390/ijms19010058 PMC5796008 29295598

[144] Boehncke WH, Schon MP (2015). Psoriasis. Lancet..

[145] Chen L, Tsai TF (2018). HLA-Cw6 and psoriasis. Br J Dermatol..

[146] Bowness P (2015). Hla-B27. Annu Rev Immunol..

[147] Suzuki E, Mellins ED, Gershwin ME, Nestle FO, Adamopoulos IE (2014). The IL-23/IL-17 axis in psoriatic arthritis. Autoimmun Rev..

[148] Aggeletopoulou I, Assimakopoulos SF, Konstantakis C, Triantos C (2018). Interleukin 12/interleukin 23 pathway: Biological basis and therapeutic effect in patients with Crohn’s disease. World J Gastroenterol..

