# Supplementary material for: Clinical, laboratory, and genetic markers for the development or presence of psoriatic arthritis in psoriasis patients: a systematic review
Source: Arthritis Res Ther. 2021 Jun 14;23:168. doi: 10.1186/s13075-021-02545-4 (PMC8201808; doi:10.1186/s13075-021-02545-4)
Supplement: Supplementary file 1 — Additional file 1: Supplementary table 1. Search strategy. [file 13075_2021_2545_MOESM1_ESM.docx]

**Supplementary table 1: Search strategy**

| **PubMed search strategy** | |
| --- | --- |
| **Psoriasis** | Psoriasis [MESH]  Psorias* [tiab]  Psoriat* AND patients [tiab] |
| AND | |
| **Risk factors (general)** | Predict*[tiab]  Risk factors [MESH]  Risk AND factor* [tiab]  Risk* [tiab]  Etiology [tiab]  Aetiology [tiab]  Aetiology [tiab] |
| OR | |
| **Risk Factors (detailed: phenotypic, laboratory and genetic)** | Biological Markers [Mesh]  Biomarker* [tiab]  Biologic* AND marker* [tiab]  Marker* [tiab]  Phenotype [MESH]  Phenotyp* [tiab]  Genetic marker [MESH]  Genetic* AND Marker [tiab] |
| AND | |
| **Psoriatic arthritis** | Psoriatic arthritis [MESH]  arthri* AND psoria* [tiab]  arthrop* AND psoria* [tiab]  enthes* AND psoria* [tiab]  Spondylarthritis [Mesh major topic]  spondyloarthr* AND psoria* [tiab] |
| AND | |
| **Limits** | Dutch[lang] OR English[lang] OR German[lang] |

| **Embase search strategy** | |
| --- | --- |
| **Psoriasis** | exp psoriasis/  psorias$.ti,ab.  (psoriat$ adj patients).ti,ab. |
| AND | |
| **Risk factors (general)** | Predict$.ti,ab.  exp risk factors/  (risk adj factor$) ti,ab.  Risk$. ti,ab.  *etiology/  etiology.ti,ab.  aetiology.ti,ab.  Determinant$.ti,ab. |
| OR | |
| **Risk Factors (detailed: phenotypic, laboratory and genetic)** | exp biological marker/  biomarker$.ti,ab.  (biologic$ adj1 marker$)  Marker$.ti,ab.  exp phenotype/  phenotyp$.ti,ab.  exp genetic marker/  (genetic$ adj1 marker).ti,ab. |
| AND | |
| **Psoriatic arthritis** | exp psoriatic arthritis/  (arthr$ adj1 psoria$).ti,ab.  (arthrop$ adj1 psoria$).ti,ab.  (enthes$ adj1 psoria$).ti,ab.  Spondylarthritis/  (spondyloarthr$ adj1 psoria$).ti,ab |
| AND | |
| **Limits** | 2: limit 1 to (conference abstract or conference paper or conference proceeding or "conference review")  1 not 2  limit .. to (dutch or english or german) |

| **Medline search strategy** | |
| --- | --- |
| **Psoriasis** | exp psoriasis/  psorias$.ti,ab.  (psoriat$ adj patients).ti,ab. |
| AND | |
| **Risk factors (general)** | Predict$.ti,ab.  exp risk factors/  (risk adj factor*).ti,ab.  Risk$.ti,ab.  etiology.ti,ab.  aetiology.ti,ab.  determinant*.ti,ab. |
| OR | |
| **Risk Factors (detailed: phenotypic, laboratory and genetic)** | exp biological marker/  biomarker$.ti,ab.  (biologic$ adj1 marker$).ti,ab.  Marker$.ti,ab.  exp phenotype/  phenotyp$.ti,ab.  exp genetic marker/  (genetic$ adj1 marker).ti,ab. |
| AND | |
| **Psoriatic arthritis** | exp psoriatic arthritis/  (arthri$ adj1 psoria$).ti,ab.  (arthrop$ adj1 psoria$).ti,ab.  (enthes$ adj1 psoria$).ti,ab.  Spondylarthritis/  (spondyloarthr$ adj1 psoria$).ti,ab. |
| AND | |
| **Limits** | limit … to (dutch or english or german) |

| **Web of science search strategy** | |
| --- | --- |
| **Psoriasis** | Psorias*  Psoriat* NEAR/1 patients |
| AND | |
| **Risk factors (general)** | Predict*  Risk NEAR/1 factor*  Risk*  Etiology  Aetiology  Determinant* |
| OR | |
| **Risk Factors (detailed: phenotypic, laboratory and genetic)** | Biologic* NEAR/1 marker*  Biomarker*  Phenotyp*  Genetic* NEAR/1 marker |
| AND | |
| **Psoriatic arthritis** | arthri* NEAR/1 Psoria*  arthrop* NEAR/1 psoria*  enthes* NEAR/1 psoria*  spondylarthritis  spondyloarthr* NEAR/1 psoria |
| AND | |
| **Limits** | Refined by: [excluding] DOCUMENT TYPES: ( MEETING ABSTRACT ) AND LANGUAGES: ( ENGLISH OR GERMAN ) |

| **Cochrane search strategy** | |
| --- | --- |
| **Psoriasis** | MeSH descriptor: [Psoriasis] explode all trees  psorias*:ti,ab,kw  psoriat* near/1 patients:ti,ab,kw |
| AND | |
| **Risk factors (general)** | predict*:ti,ab,kw  MeSH descriptor: [Risk Factors] explode all trees  risk factor*:ti,ab,kw  risk*:ti,ab,kw  etiology:ti,ab,kw  aetiology:ti,ab,kw  determinant:ti,ab,kw |
| OR | |
| **Risk Factors (detailed: phenotypic, laboratory and genetic)** | MeSH descriptor: [Biomarkers] explode all trees  biomarker*:ti,ab,kw  biologic* marker*:ti,ab,kw  marker:ti,ab,kw  MeSH descriptor: [Phenotype] explode all trees  phenotyp*:ti,ab,kw  MeSH descriptor: [Genetic Markers] explode all trees  genetic* marker:ti,ab,kw |
| AND | |
| **Psoriatic arthritis** | MeSH descriptor: [Arthritis, Psoriatic] explode all trees  arthri* near/1 psoria*:ti,ab,kw  arthrop* near/1 psoria*:ti,ab,kw  enthes* near/1 psori*:ti,ab,kw  MeSH descriptor: [Spondylarthritis] this term only  spondyloarthr* near/1 psoria*:ti,ab,kw |
