# Supplementary material for: Clinical, laboratory, and genetic markers for the development or presence of psoriatic arthritis in psoriasis patients: a systematic review
Source: Arthritis Res Ther. 2021 Jun 14;23:168. doi: 10.1186/s13075-021-02545-4 (PMC8201808; doi:10.1186/s13075-021-02545-4)
Supplement: Supplementary file 2 — Additional file 2: Supplementary table 2. Characteristics of included studies (n = 119). [file 13075_2021_2545_MOESM2_ESM.docx]

**Supplementary table 2: Characteristics of included studies (n=119)**

| **Study, design** | **Patient characteristics Psoriasis** | **Patient characteristics Psoriatic Arthritis** | **Predictors (n)** | **Main results** |
| --- | --- | --- | --- | --- |
| Abdelaal, 2018^93^  Egypt  Cross-sectional | Diagnosis: by clinical and histopathological criteria  N = 10  Age (median): 38 years  Sex: 40% male  Exclusion PsA: no | Diagnosis: by CASPAR criteria  N = 10  Age (median): 39 years  Sex: 30% male | Cytologic phenotype (3) | A higher mRNA-expression of CXCL12 in keratinocytes, dermal cells and blood vessels was associated with PsA. |
| Abdel Fattah, 2009^37^  Egypt  Cross-sectional | Diagnosis: basis not reported  N = 40  Age (mean): 43 years  Sex: 65% male  Exclusion PsA: no | Diagnosis: by Moll & Wright criteria  N = 40  Age (mean): 43 years  Sex: 45% male | ACPA (1) | Anti-CCP levels were not associated with PsA. |
| Abji, 2016^27^  Canada  Cohort  Follow-up: 1-5 years | Diagnosis: by dermatologist  N = 45  Age (mean): 46 years  Sex: 44% male  Exclusion PsA: yes | Diagnosis: by rheumatologist and CASPAR  N = 46  Age (mean): 47 years  Sex: 54% male | Cytokines (1)  Inflammation marker (1)  Disease activity (4)  Patient characteristics (4) | Baseline patient characteristics and disease activity were not associated with an increased risk of PsA.  CXCL10 was associated with PsA. |
| Abji, 2017^80^  Canada  Cross-sectional | Diagnosis: by dermatologist N = 20  Age (mean): 44 years Sex: 50% male Exclusion PsA: yes | Diagnosis: by rheumatologist, CASPAR criteria  N = 20  Age (mean): 48 years  Sex: 45% male | mRNA expression, peripheral blood (25) | A lower mRNA-expression of 15 genes was associated of PsA.^*^ |
| Abji, 2020^60^  Canada  Cohort  Follow-up: 1-10 years | Diagnosis: by dermatologist  N = 583  Age (mean): 50 years  Sex: 46% male  PsA exclusion: yes | Diagnosis: by rheumatologist, CASPAR criteria  N = 61  Age (mean): 50 years  Sex: 52% male | Cytokines (1) | A larger decline in CXCL10 over time was associated with PsA. |
| Alenius, 2005^39^  Sweden  Cross-sectional | Diagnosis: basis not reported N = 146 Age: not reported Sex: 53% male Exclusion PsA: uncertain | Diagnosis: basis not reported  N = 160  Age: not reported  Sex: 45% male | ACPA (1) | A higher anti-CCP level was associated with an increased risk of PsA. |
| Alenius, 2009^63^  Sweden  Cross-sectional | Diagnosis: basis not reported N = 85 Age (mean): 51 years Sex: 51% male^**^ Exclusion PsA: yes | Diagnosis: by Moll and Wright criteria  N = 134  Age (mean): 48 years  Sex: 51% male^**^ | Cytokines (1)  Other serum markers (1) | A higher IL-6 level was associated with an increased risk of PsA. |
| Amin, 2015^57^  Egypt  Cross-sectional | Diagnosis: basis not reported  N = 40  Age (mean): 58 years  Sex: 60% male  Exclusion PsA: yes | Diagnosis: by CASPAR criteria  N = 20  Age (mean): 46 years  Sex: 40% male | Bone metabolism (1) | A higher serum RANKL level was associated with PsA. |
| De Andrea, 2019^83^  Italy  Cross-sectional | Diagnosis: basis not reported  N = 44  Age: not reported  Sex: not reported  Exclusion PsA: no | Diagnosis: by CASPAR criteria  N = 158  Age (median): 50 years  Sex: 50% male | Serum markers (1) | A lower level of IFI16 was associated with PsA. |
| Aterido, 2019^102^  Spain  Cross-sectional | Diagnosis: by dermatologist  N= 614  Age (mean): 50 years  Sex: 60% male  Exclusion PsA: yes, not by rheumatologist | Diagnosis: by rheumatologist, CASPAR criteria  N = 2265  Age (mean): 53 years  Sex: 56% male | HLA (1) | The presence of Leu at amino acid position 95 of HLA-C was positively associated with PsA. |
| Attia, 2011^55^  Egypt  Cross-sectional | Diagnosis: by characteristic lesions  N = 34  Age (mean): 37 years  Sex: 65% male  Exclusion PsA: no | Diagnosis: by CASPAR criteria  N = 16  Age (mean): 35 years  Sex: 63% male | Bone metabolism (1) | Serum OPG was not associated with PsA. |
| Ausavarungnirun, 2017^66^  Thailand  Cross-sectional | Diagnosis: by dermatologist  N = 55  Age (mean): 52 years  Sex: 42% male  Exclusion PsA: no | Diagnosis: by rheumatologist, CASPAR criteria  N = 55 years  Age (mean): 53  Sex: 42% male | Bone metabolism (1)  Inflammation markers (2) | A higher (level of) ESR and hs-CRP were associated with PsA. |
| Barbarroja, 2019^87^  Spain  Cross-sectional | Diagnosis: by dermatologist  N = 1001  Age (mean): 48 years  Sex: 51% male  Exclusion PsA: yes | Diagnosis: by rheumatologist  N = 100  Age (mean): 50 years  Sex: 49% male | Uric acid (1) | Hyperuricemia was not associated with PsA. |
| Bartosinska, 2015^50^  Poland  Cross-sectional | Diagnosis: by dermatologist  N = 39  Age (mean): 46 years  Sex: 39% male  Exclusion PsA: yes | Diagnosis: by CASPAR criteria  N = 22  Age (mean): 48 years  Sex: 22% male | Bone metabolism (4)  Cytokines (1) | A lower OPG/sRANKL ratio was associated with PsA. |
| Bartosinka, 2018^133^  Poland  Cross-sectional | Diagnosis: by dermatologist  N = 51  Age (mean): 47 years ^**^  Sex: not reported  Exclusion PsA: no | Diagnosis: basis not reported  N = 21  Age (mean): 47 years ^**^  Sex: not reported | mRNA expression, peripheral blood (3) | mRNA expression of PDCD1, NRP1 and HLA-G was not associated with PsA. |
| Batalla, 2015^118^  Spain  Cross-sectional | Diagnosis: by 2 independent dermatologists  N = 410  Age (mean): 47 years  Sex: 54% male^**^  Exclusion PsA: yes | Diagnosis: by Moll & Wright and CASPAR criteria  N = 170  Age (mean): 47 years  Sex: 54% male^**^ | Non-HLA (6) | The presence of IL17E rs79877597 CC genotype was positively associated with PsA. |
| Benham, 2013^59^  UK  Cross-sectional | Diagnosis: by dermatologist  N = 12  Age (mean): 51 years  Sex: 50% male  Exclusion PsA: no | Diagnosis: by  CASPAR criteria  N = 11  Age (mean): 52 years  Sex: 46% male | Cell culture (1)  Cytologic phenotype (2) | A higher IL-22 secretion by PBMC’s was associated with PsA. |
| Borman, 2008^47^  Turkey  Cross-sectional | Diagnosis: by histopathology  N = 29  Age (mean): 39 years  Sex: 52% male  Exclusion PsA: yes | Diagnosis: by inflammatory arthritis  N = 18  Age (mean): 41 years  Sex: 44% male | Bone metabolism (4)  Inflammation markers (2) | A higher (level of) of ESR and CRP were associated with PsA. |
| Bose, 2014^58^  Italy  Cross-sectional | Diagnosis: basis not reported  N = 21  Age (mean): 54 years  Sex: 90% male  Exclusion PsA: no | Diagnosis by: CASPAR criteria  N = 30  Age (mean): 54 years  Sex: 67% male | Cell culture (7)  Cytologic phenotype (1) | A higher IL-2 secretion by anti-CD3-stimulated T-cells and a higher percentage of CD3+CD71+ cells were associated with PsA. |
| Bostoen, 2014^124^  Belgium  Cross-sectional | Diagnosis: by dermatologist  N = 49  Age (mean): 49 years  Sex: 59% male  Exclusion PsA: no | Diagnosis: by rheumatologist, CASPAR criteria  N = 55  Age (mean): 50 years  Sex: 69% male | Inflammation marker (1) | CRP was not associated with PsA. |
| Bowes, 2011^117^  UK, Ireland  Cross-sectional | Diagnosis: by dermatologist  N = 743  Age: not reported  Sex: not reported  Exclusion PsA: yes | Diagnosis: by rheumatologist based on psoriasis and peripheral arthritis  N = 937  Age: not reported  Sex: 42% male | Non-HLA (2) | The presence of IL-13 rs1800925 and rs20541 major allele were positively associated with PsA. |
| Bowes, 2015^121^  Multiple countries  (WTCCC2-cohort)  Cross-sectional | Diagnosis: basis not reported  N = 1784  Age: not reported  Sex: not reported Exclusion PsA: yes | Diagnosis: basis not reported  N = 1962  Age: not reported  Sex: not reported | Non-HLA (1) | The presence of PTPN22 rs2476601 was positively associated with PsA. |
| Bowes, 2017^103^  Multiple countries  (WTCCC2-cohort)  Cross-sectional | Diagnosis: not reported basis  N = 2808  Age: not reported  Sex: not reported  Exclusion PsA: yes | Diagnosis: by rheumatologist based on psoriasis and peripheral arthritis  N = 1945  Age: not reported  Sex: not reported | HLA (10) | The presence of Asp or Ser at amino acid position 97 of HLA-B was positively associated with PsA. HLA-C*0602 was negatively associated with PsA. |
| Cabaleiro, 2013^109^  Spain  Cross-sectional | Diagnosis: basis not reported  N = 109  Age: not reported  Sex: not reported  Exclusion of PsA: yes | Diagnosis: basis not reported  N = 33  Age: not reported  Sex: not reported | HLA (1)  Non-HLA (8) | The presence of TNF-857 CC phenotype was positively associated with PsA. |
| Calzavara, 1998^125^  Italy  Cross-sectional | Diagnosis: basis not reported  N = 38  Age (mean): 50 years  Sex: 63% male  Exclusion PsA: no | Diagnosis: by Moll and Wright, Ruzicka criteria  N = 76  Age (mean): 49 years  Sex: 46% male | ACPA (1) | Presence of APF was not associated with PsA. |
| Candia, 2006^38^  USA, Columbia  Cross-sectional | Diagnosis: by  dermatologist  N = 106  Age (mean): 43 years  Sex: 48% male  Exclusion PsA: no | Diagnosis: by Moll and Wright criteria  N = 72  Age (mean): 48 years  Sex: 43% male | ACPA (1) | A higher level of anti-CCP was associated with PsA. |
| Canpolat, 2010^68^  Turkey  Cross-sectional | Diagnosis: basis not reported  N = 58  Age (mean): 41 years ^**^  Sex: 53% male  Exclusion PsA: yes | Diagnosis: by Moll and Wright criteria  N = 48  Age (mean): 41 years ^**^  Sex: 58% male | Cytologic phenotype (2) | A higher MPV was associated with PsA. |
| Chandran, 2010^49^  Canada  Cross-sectional | Diagnosis: by  dermatologist  N = 26  Age (mean): 45 years  Sex: 46% male  Exclusion PsA: yes | Diagnosis: by CASPAR criteria  N = 26  Age (mean): 47 years  Sex: 46% male | Bone metabolism (6)  Cytokines (2)  Inflammation markers (1) | Higher level of hs-CRP, OPG, MMP-3 and the CPII:C2C ratio were associated with PsA. |
| Coto-Segura, 2019^107^  Spain  Cross-sectional | Diagnosis: by dermatologist  N = 309  Age: not reported  Sex: 59% male  Exclusion PsA: no | Diagnosis: by rheumatologist, CASPAR criteria  N = 187  Age: not reported  Sex: 48% male | HLA (1)  Non-HLA (3) | The presence of HLA-Cw6 was negatively associated with PsA. The presence of NFKB1A rs7152376 was positively associated with PsA. |
| Cretu, 2015^92^  Canada  Cross-sectional | Diagnosis: basis not reported  N = 10  Age: not reported  Sex: 60% male  Exclusion PsA: yes | Diagnosis: by CASPAR criteria  N = 10  Age: not reported  Sex: 60% male | Skin ( 36)  Serum markers (2) | 12/36 proteins were higher expressed in PsA lesional skin.^***^ A higher level of serum ITGB5 was associated with PsA. |
| Cretu, 2017^54^  Canada  Cross-sectional | Diagnosis: basis not reported  N = 100  Age (mean): 50 years  Sex: 55% male  Exclusion PsA: yes | Diagnosis: by CASPAR criteria  N = 100  Age (mean): 51 years  Sex: 49% male | Bone metabolism (1)  Inflammation markers (1)  Serum markers (4) | Higher levels of ITGB5, M2BP and CRP were associated with PsA. |
| Dalbeth, 2010^53^  Australia  Cross-sectional | Diagnosis: by dermatologist  N = 10  Age (median): 50 years  Sex: 60% male  Exclusion PsA: no | Diagnosis: by rheumatologist, CASPAR criteria  N = 38  Age (median): 44 years  Sex: 58% male | Bone metabolism (3)  Cytokines (1)  Inflammation marker (1  Serum markers (1) | Higher levels of CRP, DKK-1 and M-CSF were associated with PsA. |
| Dalmady, 2013^41^  Hungary  Cross-sectional | Diagnosis: basis not reported  N = 42  Age (mean): 46 years  Sex: 74% male  Exclusion PsA: yes | Diagnosis: by CASPAR criteria  N = 46  Age (mean): 45 years  Sex: 52% male | ACPA (1) | A higher level of anti-MCV titers was associated with PsA. |
| Diani, 2019^51^  Italy  Cross-sectional | Diagnosis: by dermatologist  N = 28  Age (mean): 45 years  Sex: 69% male  Exclusion PsA: no | Diagnosis: by rheumatologist, CASPAR criteria  N = 15  Age (mean): 51 years  Sex: 78% male | Bone metabolism (21) | None of the markers of bone metabolism were associated with PsA. |
| Diani, 2019^67^  Italy  Cross-sectional | Diagnosis: by dermatologist  N = 50  Age (median): 48 years  Sex: 66% male  Exclusion PsA: no | Diagnosis: basis not reported  N = 50  Age (median): 48 years  Sex: 78% male | Cytologic phenotype (27) | A higher percentage of 7^$^ and a lower percentage of 5^¥^ cell subsets were associated with PsA. |
| Eder, 2011^20^  Canada  Case-control  Follow-up: 10 years | Diagnosis: by dermatologist  N = 159  Age (mean): 48 years  Sex: 54% male  Exclusion PsA: yes | Diagnosis: by CASPAR  N = 159  Age (mean): 45 years  Sex: 56% male | Comorbidities (4)  Disease activity (2)  Fertility (5)  Intoxication (4)  Medication (6)  Patient characteristics (3)  Physical stress (13)  Psychological distress (6) | Lifting heavy loads and infections that required antibiotics were associated with an increased risk of PsA.  Smoking was associated with a decreased risk of PsA. |
| Eder, 2011^116^  Canada  Cross-sectional | Diagnosis: by dermatologist  N = 342  Age: not reported  Sex: 57% male  Exclusion PsA: yes | Diagnosis: by rheumatologist, CASPAR criteria  N = 555  Age: not reported  Sex: 59% male | Non-HLA (2) | The presence of IL-13 rs848 and rs1800925 major alleles were positively associated with PsA. |
| Eder, 2012^28^  Canada  Case-control  Follow-up: not reported | Diagnosis: by dermatologist  N = 404  Age (mean): 46 years  Sex: 56% male  Exclusion PsA: yes | Diagnosis: by psoriasis and arthritis or CASPAR  N = 728  Age (mean): 37 years  Sex: 59% male | Disease activity (9)  Intoxication (4)  Patient characteristics (1) | Smoking was associated with a decreased risk of PsA. |
| Eder, 2012^99^  Canada  Cross-sectional | Diagnosis: by dermatologist  N = 335  Age (mean): 46 years  Sex: 56% male  Exclusion PsA: yes | Diagnosis: by rheumatologist, CASPAR criteria  N = 712  Age (mean): 42 years  Sex: 58% male | HLA (18) | The presence of HLA-B*27, HLA-C*01, HLA-C*02 genotypes and HLA-B*18-C*07, HLA-B*27-C*01, HLA-B*27-C*02, HLA-B*38-C*12, HLA-B*08-C*07, and HLA-B*57-C*06 haplotypes are positively associated with PsA. HLA-C*06 and HLA-DRB1*07 were negatively associated with PsA. |
| Eder, 2012^100^  Canada  Cross-sectional | Diagnosis: by dermatologist  N = 30  Age: not reported  Sex: not reported  Exclusion PsA: yes | Diagnosis: by rheumatologist, CASPAR criteria  N = 178  Age: not reported  Sex: not reported | HLA (14) | The presence of HLA-B*27, HLA-B*38, HLA-B*39 , HLA-C*12 genotypes and HLA-B*38-C*12, HLA-B*39-C*12 and HLA-B*37-C*02 haplotypes were positively associated with PsA. |
| Eder, 2013^126^  Canada  Cross-sectional | Diagnosis: by dermatologist  N = 114  Age (mean): 52 years  Sex: 58% male  Exclusion PsA: yes | Diagnosis: by rheumatologist, CASPAR criteria  N = 125  Age (mean): 54 years  Sex: 52% male | Inflammation marker (1)  Lipid metabolism (5)  Uric acid (1) | A lower serum uric acid and a higher hsCRP were associated with PsA. |
| Eder, 2013^71^  Canada  Cross-sectional | Diagnosis: by dermatologist  N = 155  Age (median): 50 years  Sex: 54% male  Exclusion PsA: yes | Diagnosis: by rheumatologist, CASPAR criteria  N = 203  Age (median): 51 years  Sex: 61% male | Inflammation marker (1)  Lipid metabolism (6)  Serum markers (1) | Higher levels of hsCRP, adiponectin and leptin were associated with PsA. |
| Eder, 2016^18^  USA  Cohort  Follow-up: 8 years | Diagnosis: by dermatologist  N = 464  Age (mean): 47 years  Sex: 56% male  Exclusion PsA: yes | Diagnosis: by rheumatologist, CASPAR  N = 51  Age (mean): 47 years  Sex: 55% male | Comorbidities (5)  Disease activity (5)  Fertility (1)  Intoxication (4)  Medication (3)  Patient characteristics (6)  Psychological distress (1) | More severe psoriasis, low level of education and the use of systemic retinoid medications were associated with an increased risk of PsA. |
| Eder, 2017^22^  USA  Cohort  Follow-up: 8 years | Diagnosis: by dermatologist  N = 410  Age (mean): 47 years  Sex: 56% male  Exclusion PsA: yes | Diagnosis: by rheumatologist  N = 57  Age (mean): 49 years  Sex: 54% male | Disease activity (17)  Patient characteristics (3)  Psychological distress (2) | Arthralgia in women, heel pain, fatigue and stiffness were associated with an increased risk of PsA.  An increase in pain, stiffness, fatigue and functional disability were associated with an increased risk of PsA. |
| Egeberg, 2018^23^  Denmark  Cohort  Follow-up: 18 years | Diagnosis: dermatologist  N = 8742  Age (mean): 52 years  Sex: 51% male  Exclusion PsA: no | Diagnosis: rheumatologist  N = 1269  Age (mean): 52 years  Sex: 51% male | Disease activity (4) | A longer duration of cutaneous symptoms was associated with an increased risk of PsA. |
| Eiris, 2014^134^  Spain  Cross-sectional | Diagnosis: by dermatologist  N = 314  Age (mean): 46 years^**^  Sex: 55% male ^**^  Exclusion PsA: yes | Diagnosis: by rheumatologist, CASPAR criteria  N = 91  Age (mean): 46 years^**^  Sex: 55% male^**^ | Non-HLA (4) | The presence of IL23R rs2201841 AA genotype and IL23R rs11209026 GG genotype was positively associated with PsA. |
| Elkayam, 2004^101^  Israel  Cross-sectional | Diagnosis: basis not reported  N = 32  Age: not reported  Sex: not reported  Exclusion PsA: no | Diagnosis: by rheumatologist  N = 50  Age: 58 years  Sex: 60% male | HLA (30) | The presence of HLA-A*03, -B*13 and -B*38 was negatively associated with PsA. |
| Engin, 2020^127^  Turkey  Cross-sectional | Diagnosis: by dermatologist  N = 89  Age (mean): 41 years  Sex: 66% male  Exclusion PsA: yes | Diagnosis: basis not reported  N = 14  Age (mean): 43 years  Sex: 57% male | Serum markers (1) | Serum TWEAK levels are not associated with PsA. |
| Esawy, 2019^44^  Egypt  Cross-sectional | Diagnosis: by rheumatologist, characteristic lesions  N = 40  Age (mean): 74 years  Sex: 50% male  Exclusion PsA: no | Diagnosis: by rheumatologist, CASPAR and Moll & Wright criteria  N = 76  Age (mean): 45 years  Sex: 55% male | ACPA (1)  Inflammation markers (1)  Serum markers (1) | A lower level of gelsolin was associated with PsA.  Higher levels of hsCRP and BSE are associated with PsA. |
| Farrag, 2017^131^  Egypt  Cross-sectional | Diagnosis: by characteristic lesions  N = 21  Age (mean): 43 years  Sex: 58% male  Exclusion PsA: yes | Diagnosis: by CASPAR criteria  N = 24  Age (mean): 47 years  Sex: 52% male | Cytokines (1) | A higher level of serum IL-34 was associated with PsA. |
| Frasca, 2018^82^  Italy  Cross-sectional | Diagnosis: basis not reported  N = 24  Age (mean): 51 years  Sex: 46% male  Exclusion PsA: no | Diagnosis: by rheumatologist, CASPAR criteria  N = 32  Age (mean): 54 years  Sex: 59% male | Cytokines (1)  Serum markers (4) | A lower level of plasma C9 is associated with PsA. |
| Gisondi, 2011^45^  Italy  Cross-sectional | Diagnosis: by rheumatologist  N = 86  Age (mean): 52 years ^**^  Sex: 63% male^**^  Exclusion PsA: no | Diagnosis: basis not reported  N = 59  Age (mean): 52 years ^**^  Sex: 63% male^**^ | Bone metabolism (1) | 25(OH) Vitamin D is not associated with PsA. |
| Green, 2020^29^  UK  Cohort  Follow-up: 17 years | Diagnosis: by code^#^  N = 88780  Age (mean): 49 years  Sex: 48%  Exclusion PsA: no | Diagnosis: by code^#^  N = 1409  Age (mean): 45 years  Sex: 53% | Intoxication (5)  Patient characteristics (3) | Higher BMI and moderate drinking were associated with an increased risk of PsA. |
| Hein, 1991^48^  Germany  Cross-sectional | Diagnosis: basis not reported  N = 24  Age (mean): 34 years  Sex: 50% male  PsA exclusion: no | Diagnosis: basis not reported  N = 24  Age (mean): 40 years  Sex: 40% male | Bone metabolism (3) | A lower urine Hp excretion was associated with PsA. |
| Ho, 2008^108^  United Kingdom  Cross-sectional | Diagnosis: by dermatologist  N = 611  Age: not reported  Sex: 54% male  Exclusion PsA: subset | Diagnosis: by rheumatologist  N = 480  Age: not reported  Sex: 57% male | HLA (1) | The presence of HLA-C*06 was not associated with PsA. |
| Hohler, 2002^123^  Belgium, Germany  Cross-sectional | Diagnosis: basis not reported  N = 65  Age (mean): 44 years  Sex: 66 % male  PsA exclusion: no | Diagnosis: by rheumatologist, seronegative inflammatory arthritis  Age (mean): 48 years  Sex: 53% male | HLA (1) Non-HLA (4) | The presence of HLA-B*27 and haplotype TNFa6c1d3 were positively associated with PsA. |
| Hong, 2018^90^  USA  Cross-sectional | Diagnosis: by code^#^  N = 16  Age (mean): 47 years ^**^  Sex: 58% male^**^  Exclusion PsA: no | Diagnosis: basis not reported  N= 16  Age (mean): 47 years ^**^  Sex: 58% male^**^ | Serum markers (1) | A higher level of VEGFR-3 was associated with PsA. |
| Hur, 2020^75^  South Korea  Cross-sectional | Diagnosis: by clinical and histopathological criteria  N = 281  Age (mean): 40 years ^**^  Sex: 59% male^**^  Exclusion PsA: no | Diagnosis: CASPAR criteria  N = 19  Age (mean): 40 years^**^  Sex: 59% male^**^ | Inflammation markers (2) | ESR and CRP were not associated with PsA. |
| Husakova, 2015^85^  Country not reported  Cross-sectional | Diagnosis: basis not reported  N = 70  Age (mean): 46 years  Sex: 54% male  Exclusion PsA: yes | Diagnosis: by seronegative arthritis  N = 40  Age (mean): 49 years  Sex: 50% male | Serum markers (1) | A higher level of serum PRL was associated with PsA. |
| Husni, 2018^72^  USA  Cross-sectional | Diagnosis: by dermatologist  N = 145  Age (mean): 46 years  Sex: 50% male  Exclusion PsA: no | Diagnosis: by rheumatologist, CASPAR criteria  N = 198  Age (mean): 50 years  Sex: 51% male | Inflammation marker (1)  Lipid metabolism (4)  Serum markers (1) | A higher level of CRP and triglycerides, and a higher arylesterase activity were associated with PsA. |
| Isik, 2016^122^  Turkey  Cross-sectional | Diagnosis: by dermatologist  N = 71  Age: 41 years  Sex: 42% male  Exclusion PsA: no | Diagnosis: by CASPAR criteria  N = 58  Age: 49 years  Sex: 40% male | Non-HLA (2) | The presence of TNFa-238A and -308A was not associated with PsA. |
| Jadon, 2017^52^  UK, USA  Cross-sectional | Diagnosis: by dermatologist  N = 200  Age (median): 54 years  Sex: 51% male  Exclusion PsA: nee | Diagnosis: by  CASPAR criteria  N = 200  Age (median): 58 years  Sex: 52% male | Bone metabolism (3)  Cytokines (1) | A lower level of M-CSF was associated with PsA. A higher level of MMP-3 was associated with PsA. |
| Jensen, 2013^91^  Denmark  Cross-sectional | Diagnosis: by dermatologist  N = 48  Age (mean): 50 years  Sex: 54% male  Exclusion PsA: no | Diagnosis: by rheumatologist, CASPAR criteria  N = 42  Age (mean): 52 years  Sex: 43% male | Serum markers (1) | A higher level of YKL-40 was associated with PsA. |
| Johnson, 2019^64^  USA  Cross-sectional | Diagnosis: by dermatologist  N = 180  Age (median): 51 years  Sex: 50% male  Exclusion PsA: no | Diagnosis: basis not reported  N = 143  Age (median): 51 years  Sex: 56% male | Cytokines (3)  Inflammation marker (1)  Lipid metabolism (4) | A higher level of TNFα, and a lower level of adiponectin, were associated with PsA. |
| Julia, 2012^110^  Spain  Cross-sectional | Diagnosis: by dermatologist  N = 1050  Age: not reported  Sex: 59% male  Exclusion PsA: no | Diagnosis: by rheumatologist, CASPAR criteria  N = 955  Age: not reported  Sex: 54% male | HLA (1)  Non-HLA (3) | The presence of HLA-C rs10484554 was positively associated with PsA. |
| Julia, 2015^112^  Spain  Cross-sectional | Diagnosis: by dermatologist  N = 822  Age: not reported  Sex: not reported  Exclusion PsA: no | Diagnosis: by rheumatologist, CASPAR criteria  N = 1131  Age: 54 years  Sex: 51% male | Non-HLA (1) | A deletion at ADAMTS-MAGI1 was positively associated with PsA. |
| Kilic, 2017^69^  Turkey  Cross-sectional | Diagnosis: basis not reported  N = 41  Age (mean): 38 years  Sex: 54%  Exclusion PsA: no | Diagnosis: basis  not reported  N = 116  Age (mean): 48 years  Sex: 41% male | Cytologic phenotype (1) | A higher MPV was associated with PsA. |
| Kim, 2016^70^  South Korea  Cross-sectional | Diagnosis: by dermatologist  N = 111  Age (mean): 38 years  Sex: 56% male  Exclusion PsA: no | Diagnosis: by CASPAR criteria  N = 25  Age (mean): 42 years  Sex: 52% male | Cytologic phenotype (8)  Inflammation markers (2) | A higher NLR, PLR and ESR were associated with PsA. |
| Krajewska, 2019^74^  Poland  Cross-sectional | Diagnosis: by dermatologist  N = 41  Age (mean): 48 years  Sex: 54% male  Exclusion PsA: no | Diagnosis: by rheumatologist, CASPAR criteria  N = 31  Age (mean): 50 years  Sex: 48% male | Inflammation marker (2) | A higher (level of) CRP and BSE were associated with PsA. |
| Lewinson, 2017^36^  UK  Cohort  Follow-up: 0-25 years | Diagnosis: by code^#^  N = 73447  Age (mean): not reported  Sex: not reported  Exclusion PsA: no | Diagnosis: by code^#^  N = 1466  Age (mean): not reported  Sex: not reported | Psychological distress (1) | Major depressive disorder was associated with an increased risk of PsA. |
| El-Leithy, 2020^94^  Egypt  Cross-sectional | Diagnosis: by dermatologist  N = 20  Age (mean): 45 years  Sex: 60% male  Exclusion PsA: no | Diagnosis: by rheumatologist, CASPAR criteria  N = 20  Age (mean): 50 years  Sex: 60% male | Skin (2) | Higher expression of IL23R in dermis and epidermis were associated with PsA. |
| Love, 2012^33^  UK  Cohort  Follow-up:  0-15 years | Diagnosis: by code^#^  N = 74419  Age (mean): 52 years ^**^  Sex: 43% male^**^  Exclusion PsA: no | Diagnosis: by code^#^  N = 976  Age (mean): 52 years ^**^  Sex: 43% male^**^ | Patient characteristics (3) | A higher BMI was associated with an increased risk of PsA. |
| Li, 2012^32^  USA  Cohort  Follow-up: 0-14 years | Diagnosis: self-report  N = 581  Age (mean): not reported  Sex: 0% male  Exclusion PsA: no | Diagnosis: self-report  N = 157  Age (mean): not reported  Sex: 0% male | Intoxication (7) | Smoking intensity and duration were associated with an increased risk of PsA. |
| Li, 2012^34^  USA  Cohort  Follow-up: 0-14 years | Diagnosis: self-report  N = 556  Age (mean): not reported  Sex: 0% male  Exclusion PsA: no | Diagnosis: self-report  N = 146  Age (mean): not reported  Sex: 0% male | Patient characteristics (16) | A higher BMI, weight change since early adulthood, and a higher waist and hip circumference and waist-hip-ratio were associated with an increased risk of PsA. |
| Li, 2016^56^  China  Cross-sectional | Diagnosis: basis not reported  N = 20  Age (median): 52 years  Sex: 70% male  Exclusion PsA: not reported | Diagnosis: basis not reported  N = 40  Age (median): 41 years  Sex: 65% male | Bone metabolism (3)  Cytokines (8)  Cytologic phenotype (1)  Inflammation marker (2) | A higher (level of) CRP, BSE, IL-12/23 p40, IL-33, IL-34, IL-35, IL-38, TNFα, RANKL and OCP were associated with PsA. A lower (level of) IL-36a and OPG/RANKL-ratio were associated with PsA. |
| Liao, 2008^105^  Taiwan  Cross-sectional | Diagnosis: by dermatologist  N = 80  Age: not reported  Sex: 61% male  Exclusion PsA: no | Diagnosis: by rheumatologist, Moll & Wright criteria  N = 91  Age: not reported  Sex: 55% male | HLA (5) | The presence of HLA-B*27 or HLA–C*12 was associated positively with PsA. |
| Lin, 2014^73^  USA  Cross-sectional | Diagnosis: by dermatologist  N = 145  Age (mean): 46 years  Sex: 51% male  Exclusion PsA: yes | Diagnosis: by rheumatologist, CASPAR criteria  N = 198  Age (mean): 50 years  Sex: 50% male | Inflammation marker (1) | A higher level of CRP was associated with PsA. |
| Lin, 2019^79^  Taiwan  Cross-sectional | Diagnosis: by dermatologist  N= 34  Age (mean): 43 years  Sex: 71% male  Exclusion PsA: yes | Diagnosis: by rheumatologist, CASPAR criteria  N = 17  Age (mean): 48 years  Sex: 73% male | miRNA (1) | A higher expression of miR-146a-5p in monocytes was associated with PsA. |
| Loft, 2018^115^  Denmark  Cross-sectional | Diagnosis: by dermatologist  N = 151  Age (mean): 45 years  Sex: 66% male  Exclusion PsA: no | Diagnosis: by rheumatologist  N = 549  Age (mean): 46 years  Sex: 46% male | Non-HLA (52) | The presence of TNF rs361525 was positively associated with PsA. |
| Maejima, 2014^84^  Japan  Cross-sectional | Diagnosis: basis not reported  N = 31  Age (mean): 56 years  Sex: 84% male  Exclusion PsA: no | Diagnosis: by CASPAR criteria  N = 12  Age (mean): 45 years  Sex: 67% male | Serum markers (2) | A higher level of STIP1 and K17 levels were associated with PsA. |
| Maejima, 2017^89^  Japan  Cross-sectional | Diagnosis: basis  not reported  N = 23  Age (mean): 52 years  Sex: 70% male  Exclusion PsA: no | Diagnosis: by CASPAR criteria  N = 11  Age (mean): 48 years  Sex: 73% male | Inflammation markers (2) | ESR and CRP were not associated with PsA. |
| Mavropoulos, 2017^129^  Greece  Cross-sectional | Diagnosis: by dermatologist  N = 50  Age (mean): 53 years  Sex: 43% male  Exclusion PsA: no | Diagnosis: by rheumatologist, CASPAR criteria  N = 60  Age (mean): 51 years  Sex: 66% male | Cytologic phenotype (5) | Amount of regulatory B- cells was not associated with PsA. |
| Muto, 1996^96^  Japan  Cross-sectional | Diagnosis: basis not reported  N = 88  Age: not reported  Sex: 71% male  Exclusion PsA: no | Diagnosis: by Moll and Wright criteria  N = 31  Age: not reported  Sex: 57% male | Miscellaneous (2) | A higher level of IgG antibody against the C region was associated with PsA. |
| Mysliwiec, 2017^46^  Poland  Cross-sectional | Diagnosis: basis not reported  N = 72  Age (median): 53 years^**^  Sex: 67% male^**^  Exclusion PsA: no | Diagnosis: basis not reported  N = 13  Age (median): 53 years^**^  Sex: 67% male^**^ | Lipid metabolism (2) | A higher level of CER was associated with PsA. |
| Mysliwiec, 2019^130^  Poland  Cross-sectional | Diagnosis: basis not reported  N = 40  Age (mean): 49 years  Sex: 78% male^**^  Exclusion PsA: no | Diagnosis: basis not reported  N = 14  Age (mean): 55 years  Sex: 78% male^**^ | Bone metabolism (1)  Cytologic phenotype (1)  Inflammation marker (1)  Lipid metabolism (25) | A higher SFA/UFA ratio is associated with PsA. |
| Nair, 2009^111^  Multiple  Cross-sectional | Diagnosis: basis not reported  N = 3523  Age: not reported  Sex: not reported  Exclusion PsA: not reported | Diagnosis: basis not reported  N = 1755  Age: not reported  Sex: not reported | HLA (1)  Non-HLA (9) | The presence of HLA-C rs12191877, IL12B rs2082412 and IL23R rs2201841 were positively associated with PsA |
| Nguyen, 2017^31^  UK  Cohort  Follow-up: 0-20 years | Diagnosis: by code^#^  N = 218156  Age (mean): 45 years ^**^  Sex: 48% male^**^  Exclusion PsA: no | Diagnosis: by code^#^  N = 7057  Age (mean): 45 years ^**^  Sex: 48% male^**^ | Intoxication (2) | Current smoking was associated with a decreased risk of PsA. |
| Okada, 2014^106^  Multiple  Cross-sectional | Diagnosis: by dermatologist  N = 3098  Age: not reported  Sex: not reported  Exclusion PsA: no | Diagnosis: by rheumatologist, CASPAR criteria  N = 3038  Age: not reported  Sex: not reported | HLA (1) | The presence of Glu at amino acid position 45 of HLA-B*27 was positively associated with PsA. |
| Orgaz-Molina, 2013^42^  Spain  Cross-sectional | Diagnosis: by dermatologist  N = 70  Age (mean): 46 years  Sex: 54% male  Exclusion PsA: no | Diagnosis: by rheumatologist  N=79  Age (mean): 45 years  Sex: 54% male | Bone metabolism (1)  Lipid metabolism (6) | A lower level of total cholesterol was associated with PsA. |
| Ortolan, 2019^77^  Italy  Cross-sectional | Diagnosis: by dermatologist  N = 33  Age (mean): 55 years  Sex: 64% male  Exclusion PsA: no | Diagnosis: by rheumatologist, CASPAR criteria  N = 43  Age (mean): 60 years  Sex: 74% male | Lipid metabolism (7)  Serum marker (1) | A lower level of insulin and uric acid were associated with PsA. |
| Pasquali, 2020^78^  Sweden  Cross-sectional | Diagnosis: by dermatologist  N = 29  Age (mean): 40 years  Sex: 59% male  Exclusion PsA: yes | Diagnosis: by rheumatologist, CASPAR criteria  N = 28  Age (mean): 49 years  Sex: 43% male | miRNA (20) | A lower level of 10 extravesicular miRNA^§^ and a higher level of 9 extravesicular miRNA^¶^ were associated with PsA. |
| Pattison, 2007^21^  USA  Case-control  Follow-up: 10 years | Diagnosis: by not reported  N = 163  Age (mean): 46 years  Sex: 44% male  Exclusion PsA: yes | Diagnosis: by rheumatologist, CASPAR  N = 98  Age (mean): 54 years  Sex: 54% male | Comorbidities (4)  Fertility (6)  Intoxication (2)  Medication (6)  Physical stress (5)  Psychological distress (6) | A traumatic event, oral ulcerations, infectious diarrhea and vaccinations were associated with an increased risk of PsA. |
| Pietrzak, 2019^76^  Poland  Cross-sectional | Diagnosis: by dermatologist  N = 62  Age (mean): 41 years  Sex: not reported  Exclusion PsA: no | Diagnosis: by CASPAR criteria  N = 31  Age (mean): 41 years  Sex: not reported | Lipid metabolism (14) | Higher levels of apoB and oxLDL, and a higher ratio apoA:apoB, TC:HDL and LDL: HDL were associated with PsA.  A lower level of HDL-C was associated with PsA. |
| Pietrzak, 2020^62^  Poland  Cross-sectional | Diagnosis: by dermatologist  N = 62  Age (mean): 41 years  Sex: not reported  Exclusion PsA: no | Diagnosis: by CASPAR criteria  N = 31  Age (mean): 41 years  Sex: not reported | Cytokines (1)  Inflammation marker (1)  Lipid metabolism (8) | Higher levels of cholesterol, LDL, triglycerides and hsIL6, and a higher ratio LDL:HDL, were associated with PsA. |
| Pirowska, 2018^65^  Poland  Cross-sectional | Diagnosis: by dermatologist  N = 26  Age: not reported  Sex: 60% male^**^  Exclusion PsA: no | Diagnosis: by CASPAR criteria  N = 34  Age: not reported  Sex: 60% male^**^ | Cytokines (2) | A higher level of IL-23 was associated with PsA. |
| Pollock, 2015^81^  Canada  Cross-sectional | Diagnosis: basis not reported  N = 48  Age (mean): 46 years  Sex: 52% male  Exclusion PsA: no | Diagnosis: basis not reported  N = 48  Age (mean): 46 years  Sex: 52% male | mRNA expression, whole blood (18) | A lower expression of NOTCH2NL and SETD2 was associated with PsA.  A higher expression of HAT1 and P2RY5 was associated with PsA. |
| Pollock, 2011^98^  Canada  Cross-sectional | Diagnosis: by dermatologist  N = 243  Age: not reported  Sex: 59% male  Exclusion PsA: yes | Diagnosis: by rheumatologist, CASPAR criteria  N = 249  Age: not reported  Sex: 61% male | HLA (4)  Non-HLA (4) | The presence of MICA*00801 was positively associated with. |
| Pollock, 2019^136^  Canada  Cross-sectional | Diagnosis: by dermatologist  N =23  Age (mean): 51 years  Sex: 100% male  Exclusion PsA: yes | Diagnosis: by rheumatologist, CASPAR criteria  N = 13  Age (mean): 52 years  Sex: 100% male | Non-HLA (10) | Hypermethylation of LOC391322, ERICH1-AS1, PPP2R2D and PTPRN2 were negatively associated with PsA.  Hypermethylation of ELF5, SORCS2, EGFL8, NTF3, IL22 and PIP5K1C were positively associated with PsA. |
| Sag, 2018^43^  Turkey  Cross-sectional | Diagnosis: by dermatologist  N = 48  Age (mean): 43 years  Sex: 64% male  Exclusion PsA: no | Diagnosis: by rheumatologist, CASPAR criteria  N = 43  Age (mean): 46 years  Sex: 67% male | Bone metabolism (8)  Inflammation marker (2)  Serum markers (2) | A higher level of CRP was associated with PsA. |
| Shibata, 2009^40^  Japan  Cross-sectional | Diagnosis: by histopathology  N = 15  Age (mean): 50 years  Sex: 73% male  Exclusion PsA: no | Diagnosis: by Bennet criteria  N = 16  Age (mean): 52 years  Sex: 69% male | ACPA (1) | Presence of anti-CCP was not associated with PsA. |
| Simon, 2020^26^  Germany  Cohort  Follow-up: 7 years | Diagnosis: by dermatologist  N = 90  Age (mean): 45 years  Sex: 66% male  Exclusion PsA: yes | Diagnosis: by rheumatologist, CASPAR  N = 24  Age (mean): 47 years  Sex: 54% male | Disease activity (7) | Structural entheseal lesions and low cortical vBMD were associated with an increased risk of PsA. |
| Soltani-Arabshahi, 2010^24^  USA  Cohort  Follow-up: 6 years | Diagnosis: by dermatologist  N = 693  Age (mean): not reported  Sex: 38% male  Exclusion PsA: yes | Diagnosis: by patient report  N = 250  Age (mean): not reported  Sex: not reported | Disease activity (2)  Patient characteristics (1) | Younger age at psoriasis onset, higher worst ever BSA and higher BMI at age 18 years were associated with an increased risk of PsA. |
| Soto-Sanchez, 2010^113^  Spain  Cross-sectional | Diagnosis: basis not reported  N = 301  Age: not reported  Sex: not reported  Exclusion PsA: yes | Diagnosis: by rheumatologist  N = 81  Age: not reported  Sex: not reported | Non-HLA (3) | The presence of CCR2-64 rs1799864 was positively associated with PsA. |
| Spadaro, 1996^61^  Italy  Cross-sectional | Diagnosis: basis not reported  N = 15  Age: not reported  Sex: not reported  Exclusion PsA: no | Diagnosis: by rheumatoid factor negative polyarthritis  N = 47  Age (mean): 52 years  Sex: 64% male | Cytokines (2) | A higher level of IL-6 and sIL2R was associated with PsA. |
| Stuart, 2015^120^  Canada, Estonia, Germany, Sweden, USA  Cross-sectional | Diagnosis: by dermatologist  N = 3110  Age: not reported  Sex: not reported  Exclusion PsA: subset | Diagnosis: basis not reported  N = 1631  Age: not reported  Sex: not reported | HLA (1)  Non-HLA (1) | The presence of rs1050414 (near HLA-C and –B) and rs48991505 (near LOC100505817) were positively associated with PsA. |
| Thorarensen, 2017^35^  UK  Cohort  Follow-up: 19 years | Diagnosis: by code^#^  N = 70646  Age (mean): 48 years ^**^  Sex: 50% male^**^  Exclusion PsA: yes | Diagnosis: by code^#^  N = 1010  Age (mean): 48 years ^**^  Sex: 50% male^**^ | Physical stress (5) | Bone and joint trauma were associated with an increased risk of PsA. |
| Thumboo, 2002^19^  USA  Case-control  Follow-up: 2 years | Diagnosis: by dermatologist  N = 58  Age (mean): not reported  Sex: 49% male  Exclusion PsA: yes | Diagnosis: inflammatory arthritis in Pso patients  N = 40  Age (mean): not reported  Sex: 48% male | Comorbidities (4)  Disease activity (6)  Fertility (3)  Intoxication (1)  Medication (7)  Patient characteristics (1)  Physical stress (2)  Psychological distress (1) | Corticosteroid use was associated with an increased risk of PsA.  Pregnancy was associated with a decreased risk of pregnancy. |
| Tsuruta, 2017^86^  Japan  Cross-sectional | Diagnosis: by  histopathology  N = 276  Age (mean): 57 years  Sex: 72% male  Exclusion PsA: no | Diagnosis: by CASPAR criteria  N = 55  Age (mean): 57 years  Sex: 71% male | Uric acid (1) | The presence of hyperuricemia was associated with PsA. |
| Voiculescu, 2018^137^  Hungary  Cross-sectional | Diagnosis: by dermatologist  N = 69  Age (mean): 50 years^**^  Sex: 56% male^**^  Exclusion PsA: no | Diagnosis: basis not reported  N = 13  Age (mean): 50 years^**^  Sex: 56% male^**^ | Non-HLA (5) | The presence of MC4R 17782313 was positively associated with PsA. |
| Williams, 2005^119^  USA  Cross-sectional | Diagnosis: by dermatologist  N = 145  Age: not reported  Sex: not reported  Exclusion PsA: no | Diagnosis: basis not reported  N = 75  Age: not reported  Sex: not reported | Non-HLA (1) | The presence of KIR2DS1 was positively associated with PsA. |
| Wilson, 2009^25^  USA  Cohort  Follow-up: 30 years | Diagnosis: self-report  N = 1508  Age (mean): 43 years^**^  Sex: 50% male^**^  Exclusion PsA: yes | Diagnosis: self-report  N = 57  Age (mean): 43 years^**^  Sex: 50% male^**^ | Disease activity (14)  Patient characteristics (1) | Scalp lesions, nail dystrophy, involvement of intergluteal/perianal regions and a higher number of affected sites were associated with an increased risk of PsA. |
| Winchester, 2012^97^  Ireland  Cross-sectional | Diagnosis: by dermatologist  N = 214  Age: not reported  Sex: not reported  Exclusion PsA: yes | Diagnosis: by rheumatologist based on psoriasis, negative RF and arthritis  N = 359  Age: not reported  Sex: not reported | HLA (27) | The presence of HLA-C*06 was negatively associated with PsA. |
| Wu, 2015^30^  USA  Cohort  Follow-up: 16 years | Diagnosis: by code^#^  N = 573  Age (mean): not reported  Sex: 0% male  Exclusion PsA: yes | Diagnosis: by code^#^  N = 141  Age (mean): not reported  Sex: 0% male | Intoxication (5) | Regular beer consumption and heavier alcohol use were associated with an increased risk of PsA. |
| Yan, 2018^138^  USA  Cross-sectional | Diagnosis: by dermatologist  N = 479  Age (mean): 45 years  Sex: 58% male  Exclusion PsA: yes, not by rheumatologist | Diagnosis: by rheumatologist or dermatologist  N = 175  Age (mean): 48 years  Sex: 51% male | HLA (2) | The presence of HLA-C*06:02 was negatively associated with PsA. |
| Yang, 2012^114^  China  Cross-sectional | Diagnosis: by dermatologist  N = 379  Age: 39 years  Sex: 55% male  Exclusion PsA: yes | Diagnosis: by rheumatologist, CASPAR criteria  N = 595  Age: 43 years  Sex: 60% male | Non-HLA (20) | The presence of IL12B rs2082412 was negatively associated with PsA. |
| Yilmaz, 2017^88^  Turkey  Cross-sectional | Diagnosis: basis not reported  N = 47  Age (mean): 45 years ^**^  Sex: 44% male^**^  Exclusion PsA: no | Diagnosis: by CASPAR criteria  N = 23  Age (mean): 45 years ^**^  Sex: 44% male^**^ | Uric acid (1) | Serum uric level was not associated with PsA. |
| Yuan, 2019^95^  USA  Cross-sectional | Diagnosis: by clinical and histopathological criteria  N = 73  Age (mean): 47 years  Sex: 58% male  Exclusion PsA: no | Diagnosis: basis not reported  N = 22  Age: not reported  Sex: not reported | Serum marker (2) | A higher level of anti-ADAMTS-L5 and anti-LL-37 antibodies were associated with PsA. |
| Zhao, 2019^139^  China  Cross-sectional | Diagnosis: by dermatologist  N = 376  Age (mean): 41 years  Sex: 47% male  Exclusion PsA: yes | Diagnosis: by rheumatologist, CASPAR criteria  N = 379  Age (mean): 43 years  Sex: 60% male | Non-HLA (12) | The presence of IL12B rs4921485 was negatively associated with PsA. The presence of IL23R rs4655683 and NFKBIA rs12883343 were positively associated with PsA. |

^*^ CCL1, CCL20, CCL7, CX3CL1, CXCL2, IL-17C, IL-17F, IL-3, IL-6, IL-8, ISG20, MMP-3, STAT3, STAT6, SYK

^**^ Data only known for entire cohort, not reported for Pso and PsA separately.

^***^ C16ORF62, CPN2, FHL1, GPS1, ITGB5, LZIC, PAFAH1B2, PPP2R4, POSTN, SNCA, SRP14, SRPX

^$^ CD8^+^T_EMRA_CCR6^+^CXCR3^-^CD69^+^, CD8+T_EMRA_CCR6^-^CXCR3^+^CD69^-^, CD8^+^T_EM_CD69^+^, CD8^+^CD45RA^+^CXCR3^-^CCR6^-^CD69^+^, CD4^+^CD45RA^-^IL17^+^, CD8^+^CD45RA^-^IL17^+^, CD8^+^T_EM_IL17A^+^

^¥^ CD4^+^CD45RA^-^IFNγ^+^, CD8^+^T_EMRA_CXCR3^+^CCR4^-^, CD4^+^CD45RA^-^CXCR3^+^CCR4^-^, CD4^+^T_EM_CXCR3^+^CCR4^-^, CD4^+^CD45RA^-^CXCR3^+^CCR6^-^

^#^ Diagnosis by code includes record linkage, international classification of diseases (ICD)-9 codes and (THIN) read codes.

^§^ hsa-miR-92a-3p, hsa-miR-139-3p, hsa-let-7b-5p, hsa-miR-92b-3p, hsa-let-7b-3p, hsa-miR-486-5p, hsa-miR-1180-3p, hsa-miR-3158-3p, hsa-miR- 4732-3p, hsa-miR-203a

^¶^ hsa-miR-23a-3p, hsa-miR-379-5p, hsa-miR-98-5p, has-let-7e-5p, hsa-miR-29a-3p, hsa-miR-27b-3p, hsa-miR-26a-5p, hsa-miR-146a-5p

ADAMTS = a desintegrin and metalloproteinase with thrombospondin motifs; ACPA = anti citrullinated protein antibodies; anti-CCP = anti-cyclic citrullinated peptide; APF = antiperinuclear factor; Apo = apolipoprotein; Asp = aspartic acid; BMI = body mass index; BSA = body surface area; C16ORF61 = endosomal protein sorting factor like (VSP35L); C2C = collagen fragment neoepitopes Col2-3/4 (long mono); CASPAR = classification criteria for psoriatic arthritis; CCL = C-C chemokine ligand; CD = cluster of differentiation; CER = ceramide; CCR = chemokine receptor; CPII = C-propeptide of type II collagen; CPN2 = carboxypeptidase N subunit 2; CRP = C-reactive protein; CTNNBIP = catenin beta interacting protein; CX3CL = C-X3-C motif ligand; CXCL = C-X-C motif ligand; CXCR = C-X-C motif chemokine receptor; DKK = dikkop; ESR = erythrocyte sedimentation rate; FHL1 = four and a half LIM domains; Glu = glutamic acid; GPS = G protein pathway suppressor; HDL = high-density lipoprotein; HLA = human leukocyte antigen; Hp = hydroxyproline; hs = high sensitivity; IFI = interferon-inducible protein; IFN = interferon; IgG = immunoglobulin G; IL = interleukin; IL23R = interleukin 23 receptor; ISG = interferon stimulated gene; ITGB = integrin beta; K17 = keratin 17; KIR = killer-cell immunoglobulin-like receptor; LDL = low-density lipoprotein; LZIC = leucine zipper and CTNNBIP1 domain containing; M2BP = Mac-2-binding protein; MAGI = membrane-associated guanylate kinase; M-CSF = macrophage colony stimulating factor; MCV = mutated citrullinated vimentin; MICA = MHC class I polypeptide-related sequence; MMP = matrix metalloproteinase; MPV = mean platelet volume; mRNA = messenger RNA; NLR = neutrophile:lymphocyte ratio; NRP = neuropilin; OCP = osteoclast precursors; OPG = osteoprotegerin; oxLDL = oxidated LDL; PAFAH1B2 = platelet activating factor acetylhydrolase 1b catalytic subunit 2; PBMC = peripheral blood mononuclear Cells; PDCD = programmed cell death 1; PLR = platelet:lymphocyte ratio; PPP2R4 = protein phosphatase 2 phosphatase activator (PTPA); POSTN = periostin; PRL = prolactin; PsA = psoriatic arthritis; Pso = psoriasis; PTPN22 = protein tyrosine phosphatase non-receptor type 22; RANKL = receptor activator of nuclear factor kappa-B ligand; RF = rheumatoid factor; RNA = ribonucleic acid; Ser = serine; SFA = saturated fatty acids; SNCA = synuclein alpha; sIL-2R = soluble IL-2 receptor; sRANKL = soluble RANKL; SRP = signal recognition particle; SRPX = sushi repeat containing protein X-linked; STAT = signal transducer and activator of transcription; STIP = stress-inducible phosphoprotein; SYK = spleen associated tyrosine kinase; TC = total cholesterol; T_EM_ = effector memory T cell; T_EMRA_ = T_EM_ re-expressing CD45RA; TNF = tumor necrosis factor; TWEAK = TNF-like weak inducer of apoptosis; UFA = unsaturated fatty acids; UK = United Kingdom; USA = United States of America; vBMD = volumetric bone mineral density; VEGFR = vascular endothelial growth factor receptor; WTCCC = Welcome Trust Case Control Consortium.
