# Supplementary material for: Clinical, laboratory, and genetic markers for the development or presence of psoriatic arthritis in psoriasis patients: a systematic review
Source: Arthritis Res Ther. 2021 Jun 14;23:168. doi: 10.1186/s13075-021-02545-4 (PMC8201808; doi:10.1186/s13075-021-02545-4)
Supplement: Supplementary file 3 — Additional file 3: Supplementary table 3. Statistical significance and effect sizes of clinical markers. [file 13075_2021_2545_MOESM3_ESM.docx]

**Supplementary table 3: Statistical significance and effect sizes of clinical markers**

| **Category** | **Marker** | **Study (ref)** | **Significance** | **Effect Size** |
| --- | --- | --- | --- | --- |
| **Comorbidities** | Infection requiring | (20) | P = 0.046 | OR = 1.72 (1.01-2.95) |
|  | antibiotics | (18) | P = 0.72 | HR = 1.15 (0.53-2.49) |
|  | Diarrhoea | (20) | P = 0.19 | OR = 1.7 (0.76-3.79) |
|  |  | (21) | P = Not reported | OR = 0.23 (0.05-1.06) |
|  |  | (18) | P = 0.08 | HR = 3.18 (0.88-11.4) |
|  | Diabetes | (18) | P = 0.32 | HR = 0.53 (0.15-1.84) |
|  |  | (19) | P = 0.403 | OR = 0.40 (0.05-3.42) |
|  | Uveitis | (18) | P = 0.0002 | RR = 31.5 (5.06 – 195.8) |
| **Disease characteristics** | Age at Pso onset | (24) | P = < 0.001 | OR = 0.98 (0.96-0.98) |
|  |  | (25) | P = 0.25 | RR = 0.91 (0.77-1.07) |
|  | Fatigue (MFSS > 5) | (22) | P = 0.007 | OR = 2.36 (1.27-4.39) |
|  | Worsening fatigue | (22) | P = 0.001 | OR= 1.27 (1.09-1.57) |
|  | Heel pain | (22) | P = 0.02 | OR = 4.18 (1.26-13.8) |
|  | Stiffness (VAS>2) | (22) | P = 0.045 | OR = 2.03 (1.02-4.06) |
|  | Arthralgia | (22) | P = 0.02 | Not reported |
|  | Arthralgia men | (22) | P = Not reported | RR = 0.52 (0.12-2.22) |
|  | Arthralgia women | (22) | P = Not reported | RR = 2.59 (1.15-5.88) |
|  | Worsening stiffness | (22) | P = 0.03 | RR = 1.21 (1.02-1.42) |
|  | Psoriatic nail lesion | (18) | P = 0.31 | HR = 1.36 (0.76-2.45) |
|  |  | (19) | P = 0.752 | OR = 1.16 (0.46-2.92) |
|  |  | (27) | P = 0.68 | OR = 1.2 (0.5-3.2) |
|  |  | (25) | P = Not reported | RR = 2.24 (1.26-3.98) |
|  | Nail pitting | (18) | P = 0.0007 | HR = 2.21 (1.24-3.92) |
|  | Worsening pain | (22) | P = < 0.001 | RR = 1.34 (1.14-1.57) |
|  | Worsening function | (22) | P = 0.04 | RR = 0.96 (0.92-0.99) |
|  | Severe Pso | (20) | P = 0.89 | OR = 0.89 (0.49-1.61) |
|  |  | (28) | P = < 0.0001 | OR = 2.07 (1.47-2.97) |
|  | Duration of Pso | (27) | P = 0.99 | OR = 1 (1.0-1.0) |
|  | Duration of Pso, 6-10 years | (28) | P = 0.09 | OR = 0.70 (0.47-1.06) |
|  | Duration of Pso, 11-15 years | (28) | P = 0.003 | OR = 0.49 (0.31-0.79) |
|  | Duration of Pso, 16-20 years | (28) | P = 0.002 | OR = 0.46 (0.28-0.74) |
|  | Duration of Pso, 21 years and more | (28) | P = <0.0001 | OR = 0.37 (0.26-0.53) |
|  | PASI | (22) | P = 0.03 | OR = 1.05 (1.01-1.09) |
|  |  | (27) | P = 0.57 | OR = 1.0 (0.9-1.1) |
|  | PASI 10-20 vs <10 | (18) | P = 0.73 | HR = 1.16 (0.50-2.64) |
|  | PASI >20 vs <20 | (18) | P = 0.0006 | HR = 5.39 (1.64-17.7) |
|  | Scalp lesions | (27) | P = 0.98 | OR = 1.0 (0.4-3.0) |
|  |  | (25) | P = Not reported | RR = 3.75 (2.09-6.71) |
|  | Intergluteal lesions | (25) | P = Not reported | RR = 1.95 (1.07-3.56) |
|  | Number of Pso sites 2  Number of Pso sites = > 3  Structural entheseal lesion  Cortical vBMD entheseal | (25) | P = Not reported | RR = 0.77 (0.37-1.64) |
|  |  | (25) | P = Not reported | RR = 2.24 (1.23-4.08) |
|  |  | (26) | P = 0.008 | HR = 5.10 (1.53-16.99) |
|  |  | (26) | P = Not reported | HR = 0.64 (0.42-0.98) |
|  | Early (<20 years) vs late onset pso (dermatologist) | (26) | P = <0.001 | OR = 0.52 (0.39-0.69) |
|  | Early (<30 years) vs late onset pso (dermatologist) | (26) | P = 0.051 | OR = 0.84 (0.71-1.0) |
|  | Early (<20 years) vs late onset pso (cutaneous symptoms) | (26) | P = 0.777 | OR = 1.03 (0.84-1.27) |
|  | Early (<30 years) vs late onset pso (cutaneous symptoms) | (26) | P = 0.018 | OR = 1.22 (1.03-1.45) |
| **Fertility** | Menopause | (20) | P = 0.50 | OR = 1.8 (0.31-11.49) |
|  |  | (18) | P = 0.75 | OR = 1.19 (0.40-3.53) |
|  |  | (19) | P = 0.86 | OR = 0.89 (0.26-3.10) |
|  | Pregnancy | (20) | P = 0.73 | OR = 1.2 (0.47-2.09) |
|  |  | (19) | P = 0.04 | OR = 0.19 (0.04-0.95) |
|  |  | (21) | P = Not reported | OR = 1.06 (0.44-2.55) |
|  | Oral contraceptives | (20) | P = 0.23 | OR = 1.7 (0.71-4.23) |
|  |  | (19) | P = 0.15 | OR = 2.9 (0.68-12.28) |
|  |  | (21) | P = Not reported | OR = 1.4 (0.55-3.5) |
|  | Hormone replacement therapy | (20) | P = 0.83 | OR = 1.1 (0.5-2.39) |
|  |  | (21) | P = Not reported | OR = 1.38 (0.53-3.6) |
|  | Fertility treatment | (20) | P =0.83 | OR = 0.9 (0.26-2.98) |
|  |  | (21) | P = Not reported | OR = 0.17 (0.04-0.79) |
| **Intoxication** | Alcohol social (= 1 or more per week vs none) | (20) | P = 0.73 | OR = 0.9 (0.56-1.50) |
|  |  | (20) | P = 0.92 | HR = 1.02 (0.40-2.59) |
|  |  | (28) | P = 0.67 | OR = 0.94 (0.68-1.28) |
|  | Alcohol daily (= 1 or more per day vs none) | (20) | P = 0.96 | OR 1.0 (0.42-2.51) |
|  |  | (20) | P = 0.97 | HR = 1.02 (0.40-2.59) |
|  |  | (28) | P = 0.05 | OR = 1.65 (0.99-2.67) |
|  | Weekly alcohol use | (19) | P = 0.57 | OR = 0.77 (0.32-1.89) |
|  | Alcohol >35 units/week | (21) | P = Not reported | OR = 0.57 (0.27-1.20) |
|  | Alcohol: none vs 0-15 g/day | (30) | P = Not reported | RR = 0.75 (0.50-1.12) |
|  | Alcohol: none vs 15-30 g/day | (30) | P = Not reported | RR = 1.09 (0.48-2.47) |
|  | Alcohol: none vs >30 g/day | (30) | P = Not reported | RR = 2.09 (0.90-4.84) |
|  | Alcohol: 0-15 vs 15-30 g/day | (30) | P = Not reported | RR = 1.45 (0.67-3.16) |
|  | Alcohol: 0-15 vs >30 g/day | (30) | P = Not reported | RR = 2.79 (1.24-6.26) |
|  | Moderate drinker (=0.1-3 units/day) vs none | (29) | P = 0.0033 | OR= 1.57 (1.16-2.11) |
|  | Heavy drinker (=>3 units/day) vs none | (29) | P = 0.82 | OR = 0.94 (0.56-1.58) |
|  | Current smoking | (31) | P = Not reported | HR = 0.91 (0.84-0.99) |
|  |  | (27) | P = Not reported | OR = 1.62 (1.00-2.63) |
|  |  | (28) | P = 0.002 | OR = 0.57 (0.41-0.81) |
|  |  | (18) | P = 0.38 | HR = 1.36 (0.68-2.73) |
|  |  | (20) | P = 0.038 | OR = 0.54 (0.31-0.96) |
|  |  | (29) | P = 0.54 | OR = 0.94 (0.76-1.16) |
|  | Ex-smokers | (31) | P = Not reported | HR = 1.07 (0.97-1.18) |
|  |  | (27) | P = Not reported | OR = 1.39 (0.89-2.16) |
|  |  | (28) | P = 0.21 | OR = 0.81 (0.56-1.12) |
|  |  | (18) | P = 0.87 | OR = 1.05 (0.56-1.99) |
|  |  | (20) | P = 0.015 | OR = 0.52 (0.31-0.88) |
|  |  | (29) | P = 0.073 | OR = 0.83 (0.69-1.02) |
|  | Current smokers 1-14 cig/day | (27) | P = Not reported | OR = 1.22 (0.58-2.56) |
|  | Current smokers =/> 15 cig/day | (27) | P = Not reported | OR = 1.93 (1.09-3.4) |
|  | Smoking duration < 25 years | (27) | P = Not reported | OR = 1.35 (0.9-2.04) |
|  | Smoking duration =/>25 years | (27) | P = Not reported | OR = 1.9 (1.09-3.33) |
|  | Pack-years <20 | (27) | P = Not reported | OR = 1.22 (0.79-1.89) |
|  | Pack-years =/>20 | (27) | P = Not reported | OR = 2.02 (1.24-3.29) |
|  | Smoking at or before arthritis | (21) | P = Not reported | OR = 0.68 (0.39-1.17) |
| **Medication** | Retinoid use (ever) | (18) | P = 0.02 | HR = 3.42 (1.24-9.44) |
|  | Methotrexate use | (18) | P = Not reported | Not reported |
|  |  | (19) | P = Not reported | Not reported |
|  | Corticosteroids use | (19) | P = 0.015 | OR = 4.33 (1.34-14.02) |
|  | Influenza vaccination | (20) | P = 0.87 | OR = 1.0 (0.58-1.57) |
|  |  | (21) | P = Not reported | OR = 0.40 (0.14-1.14) |
|  | Rubella vaccination | (20) | P = 0.81 | OR = 0.8 (0.22-3.32) |
|  |  | (21) | P = Not reported | OR = 12.4 (1.20-122.14) |
|  | Tetanus vaccination | (20) | P = 0.87 | OR = 1.1 (0.29-4.24) |
|  |  | (21) | P = Not reported | OR = 1.91 (1.0-3.7) |
| **Patient characteristics** | Age | (20) | P = 0.29 | OR = 0.99 (0.97-1.01) |
|  |  | (22) | P = 0.61 | RR = 0.99 (0.96-1.02) |
|  |  | (25) | P = 0.15 | RR = 0.76 (0.54-1.08) |
|  |  | (27) | P = 0.38 | OR = 1.0 (0.9-1.0) |
|  | BMI | (22) | P = 0.11 | RR = 1.05 (0.99-1.13) |
|  | BMI >30 | (27) | P = 0.39 | OR = 1.5 (0.6-4.2) |
|  | BMI 25-30 vs <25 | (33) | P = <0.001 | RR = 1.09 (0.93-1.28) |
|  |  | (34) | P = Not reported | OR = 1.81 (1.23-2.93) |
|  |  | (29) | P = <0.001 | OR = 1.76 (1.41-2.19) |
|  | BMI 30-35 vs <25 | (33) | P = <0.001 | RR = 1.22(1.02-1.47) |
|  |  | (34) | P = Not reported | OR = 1.90 (1.13-3.18) |
|  |  | (29) | P = <0.001 | OR = 2.04 (1.60-2.60) |
|  | BMI >35 vs <25 | (33) | P = <0.001 | RR = 1.48 (1.2-1.81) |
|  |  | (34) | P = Not reported | OR = 2.98 (1.86-4.78) |
|  |  | (29) | P = <0.001 | OR = 2.42 (1.85-3.16) |
|  | Overweight vs normal | (18) | P = 0.95 | HR = 1.02 (0.50-2.10) |
|  | Obese vs normal | (18) | P = 0.1 | HR = 1.76 (0.89-3.47) |
|  | BMI at age 18, <21 | (34) | P = Not reported | OR = 1.28 (0.79-2.06) |
|  | BMI at age 18, 23.0-24.9 vs <21 | (34) | P = Not reported | OR = 1.73 (0.96-3.13) |
|  | BMI at age 18, 25.0-29.9 vs <21 | (34) | P = Not reported | OR = 1.69 (0.88-3.26) |
|  | BMI at age 18, = or > 30 vs <21 | (34) | P = Not reported | OR = 1.53 (0.71-3.29) |
|  | BMI at 18 years | (24) | P = < 0.01 | OR = 1.06 (1.02-1.10) |
|  | Waist circumference  (28.0-31.9 inch vs < 28.0 inch) | (34) | P = Not reported | OR = 1.46 (0.54-3.99) |
|  | Waist circumference  (> 32.0 vs < 28.0 inch) | (34) | P = Not reported | OR = 3.02 (1.21-7.56) |
|  | Hip circumference  (38.0-40.9 inch vs < 38.0 inch) | (34) | P = Not reported | OR = 1.24 (0.51-3.0) |
|  | Hip circumference  (>41.0 inch vs < 38.0 inch) | (34) | P = Not reported | OR = 2.59 (1.18-5.69) |
|  | Waist-hip ratio  (0.744-0.800 vs < 0.744) | (34) | P = Not reported | OR = 1.41 (0.63-3.15) |
|  | Waist-hip ratio  (>0.800 vs <07.44) | (34) | P = Not reported | OR = 2.48 (1.20-5.15) |
|  | Weight change from 18 years  (increase 20-49.9 lb vs < 20 lb) | (34) | P = Not reported | OR = 1.34 (0.82-2.17) |
|  | Weight change from 18 years  (increase 50-99.9 lb vs < 20 lb) | (34) | P = Not reported | OR = 2.42 (1.49-3.91) |
|  | Weight change from 18 years  (increase 100 lb vs < 20 lb) | (34) | P = Not reported | OR = 3.84 (1.93-7.63) |
|  | University level of education | (20) | P = 0.56 | OR = 1.18 (0.66-2.13) |
|  | High school graduate vs | (18) | P = 0.049 | HR = 0.30 (0.09-0.99) |
|  | University vs high school incomplete | (18) | P = 0.005 | HR = 0.22 (0.08-0.62) |
|  | Patient reported family history of PsA | (18) | P = 0.29 | HR = 1.96 (0.57-6.71) |
|  |  | (27) | P = 0.85 | OR = 1.2 (0.1-10.5) |
|  | Female sex | (18) | P = 0.86 | OR = 0.86 (0.53-1.42) |
|  |  | (28) | P = 0.85 | OR = 1.03 (0.78-1.36) |
|  | Male sex | (22) | P = 0.41 | RR = 1.46 (0.59-3.63) |
|  |  | (27) | P = 0.75 | OR = 1.2 (0.5-2.9) |
| **Physical stress** | Fracture | (20) | P = 0.69 | OR = 1.2 (0.54-2.51) |
|  |  | (19) | P = 0.41 | OR = 1.50 (0.58-3.91) |
|  |  | (21) | P = Not reported | OR = 1.0 (0.34-2.96) |
|  |  | (35) | P = Not reported | RR = 1.46 (1.04-2.04) |
|  | Lifting heavy loads (>100 pounds/hour) | (20) | P = 0.0008 | OR = 2.92 (1.56-5.46) |
|  | Any trauma | (20) | P = 0.054 | OR = 1.97 (0.99-3.96) |
|  |  | (21) | P = Not reported | OR = 1.10 (0.65-1.86) |
|  |  | (35) | P = Not reported | RR = 1.32 (1.13-1.54) |
|  | Trauma leading to medical care | (21) | P = Not reported | OR = 2.53 (1.1-6.0) |
|  | Joint trauma | (35) | P = Not reported | RR = 1.50 (1.19-1.90) |
| **Psychological distress** | Death of family member | (20) | P = 0.82 | OR = 1.1 (0.63-1.79) |
|  |  | (21) | P = Not reported | OR = 1.1 (0.6-2.0) |
|  | Move to a new home | (20) | P = 0.68 | OR = 1.1 (0.67-1.82) |
|  |  | (21) | P = Not reported | OR = 2.29 (1.21-4.4) |
|  | Changed job | (20) | P = 0.44 | OR = 1.2 (0.73-2.07) |
|  |  | (21) | P = Not reported | OR = 1.72 (0.85-3.5) |
|  | Becoming unemployed | (21) | P = Not reported | OR = 1.92 (0.6-6.0) |
|  | Becoming employed | (20) | P = 0.85 | OR = 1.0 (0.54-1.66) |
|  | Treated for anxiety/depression | (20) | P = 0.41 | OR = 0.8 (0.39-1.45) |
|  |  | (21) | P = Not reported | OR = 0.67 (0.27-1.7) |
|  | Depression | 121 | P = 0.021 | HR = 1.37 (1.05-1.80) |
|  |  | (18) | P = 0.85 | HR = 0.92 (0.35-2.34) |
|  | Psychological distress | (22) | P = 0.11 | RR = 1.17 (0.89-3.35) |
|  |  | (19) | P = 0.87 | OR = 0.93 (0.36-2.36) |

BMI = Body mass index; IB = international pound; Cig = cigarettes; PASI = Psoriasis Area and Severity Index; Pso = psoriasis; PsA = psoriatic arthritis; vBMD = volumetric bone mineral density.
