# Supplementary material for: Clinical, laboratory, and genetic markers for the development or presence of psoriatic arthritis in psoriasis patients: a systematic review
Source: Arthritis Res Ther. 2021 Jun 14;23:168. doi: 10.1186/s13075-021-02545-4 (PMC8201808; doi:10.1186/s13075-021-02545-4)
Supplement: Supplementary file 4 — Additional file 4: Supplementary table 4. Statistical significance and effect sizes of laboratory markers. [file 13075_2021_2545_MOESM4_ESM.docx]

**Supplementary table 4: Statistical significance and effect sizes of laboratory markers**

| **Category** | **Marker** | **Study** | **Significance** | **Effect Size** |
| --- | --- | --- | --- | --- |
| **ACPA** | Anti-CCP | (39) | P = 0.006 | Not reported |
|  |  | (37) | P = 0.012 | Not reported |
|  |  | (38) | P = < 0.001 | Not reported |
|  |  | (40) | Not significant | Not reported |
| **Bone metabolism** | 25(OH) Vitamin D | (42) | P = 0.083 | Not reported |
|  |  | (46) | Not significant | Not reported |
|  |  | (43) | P = 0.685 | Not reported |
|  |  | (45) | P = 0.4 | Not reported |
|  | 25(OH) Vitamin D < 20 mg/L | (43) | P = 0.090 | Not reported |
|  | 25(OH) Vitamin D 20-30 mg/L | (43) | P = 0.795 | Not reported |
|  | 25(OH) Vitamin D > 30 mg/L | (43) | P = 0.876 | Not reported |
|  | Alkalic Phospate | (47) | P = 0.231 | Not reported |
|  |  | (47) | Not significant | Not reported |
|  |  | (43) | P = 0.234 | Not reported |
|  | Calcium | (47) | P = 0.47 | Not reported |
|  |  | (47) | Not significant | Not reported |
|  |  | (43) | P = 0.207 | Not reported |
|  | COMP | (50) | P = 0.145 | Not reported |
|  |  | (49) | P = 0.35 | OR = 1.000 |
|  | CPII:C2C | (49) | P = 0.01 | OR = 12.031 |
|  | CTX | (47) | P = 0.169 | Not reported |
|  | CTx-I | (51) | Not significant | Not reported |
|  | CTx-II | (51) | Not significant | Not reported |
|  | DKK-1 | (52) | P = 0.07 | OR = 1.14 |
|  |  | (53) | P = < 0.001 | Not reported |
|  |  | (51) | Not significant | Not reported |
|  | MMP3 | (52) | P = 0.0004 | OR = 1.02 |
|  |  | (49) | P = 0.04 | OR = 1.323 |
|  |  | (54) | P = 1.00^E^-03 | OR = 1.59 |
|  |  | (51) | Not significant | Not reported |
|  | OPG | (52) | P = 0.17 | OR = 2.51 |
|  |  | (50) | P = 0.986 | Not reported |
|  |  | (49) | P = 0.04 | OR = 1.323 |
|  |  | (55) | P = 0.77 | Not reported |
|  |  | (53) | Not significant | Not reported |
|  |  | (51) | Not significant | Not reported |
|  | OPG/RANKL ratio | (50) | P = 0.049 | OR = 0.92 |
|  |  | (56) | P = < 0.001 | Not reported |
|  | Osteoclast precursor | (56) | P = < 0.001 | Not reported |
|  | Phosphate | (47) | P = 0.456 | Not reported |
|  |  | (43) | P = 0.541 | Not reported |
|  | RANKL | (57) | P = < 0.001 | Not reported |
|  |  | (50) | P = 0.221 | Not reported |
|  |  | (49) | P = 0.77 | OR = 0.999 |
|  |  | (53) | Not significant | Not reported |
|  |  | (51) | Not significant | Not reported |
|  |  | (56) | P = < 0.001 | Not reported |
|  | Urine Hp | (47) | P = < 0.05 | Not reported |
| **Cell culture** | PBMC’s: IL-17 secretion | (59) | P = < 0.05 | Not reported |
|  | T-cells: IFNy secretion | (58) | P = 0.367 | Not reported |
|  | T-cells: IL-2 secretion | (58) | P = 0.023 | Not reported |
|  | T-cells: IL-4 secretion | (58) | P = 0.27 | Not reported |
|  | T-cells: IL-5 secretion | (58) | P = 0.695 | Not reported |
|  | T-cells: IL-10 secretion | (58) | P = 0.285 | Not reported |
|  | T-cells: IL-17 secretion | (58) | P = 0.16 | Not reported |
|  | T-cells: TNFa secretion | (58) | P = 0.64 | Not reported |
| **Cytokines** | CXCL10 | (27) | P = 0.004 | OR = 1.3 |
|  | CXCL10 decline over time | (60) | P = < 0.001 | Not reported |
|  | IL-6 | (63) | P = 0.002 | Not reported |
|  |  | (61) | P = 0.05 | Not reported |
|  | IL-6 (high) | (64) | Not significant | OR = 1.28 |
|  | IL-6 (hs) | (62) | P = <0.01 | Not reported |
|  | IL-12p40 | (49) | P = 0.12 | OR = 1.014 |
|  |  | (56) | P = < 0.05 | Not reported |
|  | IL-23 | (65) | P = 0.0038 | Not reported |
|  | IL-33 | (56) | P = < 0.05 | Not reported |
|  | IL-34 | (66) | P = 0.001 | Not reported |
|  |  | (56) | P = < 0.001 | Not reported |
|  | IL-35 | (56) | P = < 0.01 | Not reported |
|  | IL-36a | (56) | P = < 0.001 | Not reported |
|  | IL-38 | (56) | P = < 0.001 | Not reported |
|  | M-CSF | (52) | P = 0.01 | OR = 0.44 |
|  |  | (53) | P = < 0.01 | Not reported |
|  | TNF-a | (64) | P = < 0.001 | Not reported |
|  |  | (56) | P = < 0.001 | Not reported |
|  | TNF-a (high) | (64) | P = > 0.05 | OR = 2.25 |
| **Cytologic phenotype** | CD3+CD71+ count | (58) | P = 0.034 | Not reported |
|  | CD4+CD45RA-CXCR3+CCR4- | (67) | P = 0.001 | Not reported |
|  | CD4+CD45RA-CXCR3+CCR6- | (67) | P = 0.025 | Not reported |
|  | CD4+CD45RA-IFNy+ | (67) | P = 0.015 | Not reported |
|  | CD4+CD45RA-IL17+ | (67) | P = 0.034 | Not reported |
|  | CD4+T_EM_CXCR3+CCR4- | (67) | P = 0.037 | Not reported |
|  | CD4+T_EM_IL17A+ | (67) | P = 0.029 | Not reported |
|  | CD8+CD45RA-CCR6+CXCR3-CD69+ | (67) | P = 0.026 | Not reported |
|  | CD8+CD45RA-IL17+ | (67) | P = 0.005 | Not reported |
|  | CD8+T_CM_CD69+ | (67) | P = 0.035 | Not reported |
|  | CD8+T_EM_IL17A+ | (67) | P = 0.034 | Not reported |
|  | CD8+T_EMRA_CCR6+CXCR3-CD69- | (67) | P = 0.0001 | Not reported |
|  | CD8+T_EMRA_CXCR3+CCR4- | (67) | P = 0.018 | Not reported |
|  | CD8+T_EMRA_CXCR3+CCR6-CD69+ | (67) | P = 0.01 | Not reported |
|  | Mean platelet volume | 5257 | P = 0.072 | Not reported |
|  |  | (68) | P = < 0.001 | Not reported |
|  | Monocyte count | (70) | P = 0.0172 | Not reported |
|  | Neutrophil count | (70) | P = < 0.0001 | Not reported |
|  | Neutrophil:lymphocyte ratio | (70) | P = 0.0002 | Not reported |
|  | Platelet count | (70) | P = 0.001 | Not reported |
|  |  | (68) | P = 0.09 | Not reported |
|  | Platelet:lymphocyte ratio | (70) | P = 0.0227 | OR = 1.012 |
|  | White blood count | (70) | P = < 0.0001 | Not reported |
|  |  | (46) | Not significant | Not reported |
| **mRNA expression whole blood** | CX3CL1 | (80) | P = 0.046 | Not reported |
|  | CXCL2 | (80) | P = 0.002 | Not reported |
|  | CXCL5 | (80) | P = 0.042 | Not reported |
|  | CXCL10 | (81) | P = 0.23 | Not reported |
|  | HAT1 | (81) | P = 0.02 | Not reported |
|  | IL3 | (80) | P = 0.021 | Not reported |
|  | IL6 | (80) | P = 0.044 | Not reported |
|  | IL8 | (80) | P = 0.001 | Not reported |
|  | IL17C | (80) | P = 0.009 | Not reported |
|  | IL17F | (80) | P = 0.014 | Not reported |
|  | ISG20 | (80) | P = 0.008 | Not reported |
|  | MMP3 | (80) | P = 0.001 | Not reported |
|  | NFKB1 | (80) | P = 0.581 | Not reported |
|  | NOTCH2NL | (81) | P = < 0.001 | Not reported |
|  | SETD2 | (81) | P = 0.03 | Not reported |
|  | STAT3 | (80) | P = 0.022 | Not reported |
|  | STAT6 | (80) | P = 0.035 | Not reported |
|  | SYK | (80) | P = 0.004 | Not reported |
|  | TBX21 | (80) | P = 0.004 | Not reported |
| **Inflammation marker** | CRP | (27) | P = 0.147 | Not reported |
|  |  | (70) | P = < 0.0001 | Not reported |
|  |  | (54) | P = 2.55 x 10^E^-07 | OR = 1.96 |
|  |  | (47) | P = < 0.05 | Not reported |
|  |  | (73) | P = 0.001 | Not reported |
|  |  | (53) | P = < 0.05 | Not reported |
|  |  | (74) | P = < 0.001 | Not reported |
|  |  | (46) | Not significant | Not reported |
|  |  | (75) | P = 0.487 | OR = 0.398 |
|  |  | (72) | P = 0 .001 | Not reported |
|  |  | (43) | P = < 0.001 | Not reported |
|  |  | (1f36) | P = <0.05 | Not reported |
|  |  | (58) | Not significant | Not reported |
|  |  | (64) | Not significant | OR = 1.24 |
|  | CRP (high) | (49) | P = 0.03 | OR = 2.402 |
|  | hs-CRP | (66) | P = 0.01 | Not reported |
|  |  | (71) | P = < 0.001 | Not reported |
|  |  | (44) | P = 0.008 | Not reported |
|  |  | (70) | P = < 0.0001 | OR = 1.036 |
|  | ESR | (66) | P = < 0.001 | Not reported |
|  |  | (47) | P = < 0.05 | Not reported |
|  |  | (74) | P = 0.017 | Not reported |
|  |  | (44) | P = < 0.0001 | Not reported |
|  |  | (75) | P = 0.600 | OR = 0.984 |
|  |  | (43) | P = 0.066 | Not reported |
|  |  | (62) | P = 0.57 | Not reported |
|  |  | (56) | P = <0.05 | Not reported |
|  |  | (71) | P = 0.005 | Not reported |
| **Lipid metabolism** | Adiponectin | (64) | P = 0.12 | Not reported |
|  |  | (64) | P = < 0.05 | OR = 0.61 |
|  | Adiponectin (high) | (76) | P = < 0.05 | Not reported |
|  | ApoA:ApoB | (76) | P = < 0.05 | Not reported |
|  | ApoB | (46) | P = 0.003 | Not reported |
|  | CER | (42) | P = 0.068 | Not reported |
|  | Glucose | (71) | P = 0.12 | Not reported |
|  |  | (46) | Not significant | Not reported |
|  |  | (76) | P = 0.0519 | Not reported |
|  |  | (62) | P = 0.08 | Not reported |
|  |  | (77) | P = 0.49 | Not reported |
|  | Glucose (fasting) | (42) | P = 0.196 | Not reported |
|  | HDL | (71) | P = 0.69 | Not reported |
|  |  | (77) | P = 0.1 | Not reported |
|  |  | (72) | P = 0.627 | Not reported |
|  |  | (62) | P = 0.25 | Not reported |
|  |  | (77) | P = 0.02 | Not reported |
|  | Insulin | (42) | P = 0.087 | Not reported |
|  | LDL | (71) | P = 0.52 | Not reported |
|  |  | (46) | P = 0.36 | Not reported |
|  |  | (72) | P = 0.192 | Not reported |
|  |  | (76) | P = 0.0798 | Not reported |
|  |  | (62) | P = < 0.05 | Not reported |
|  |  | (76) | P = < 0.01 | Not reported |
|  | LDL:HDL | (62) | P = < 0.05 | Not reported |
|  |  | (71) | P = 0.04 | Not reported |
|  | Leptin | (64) | Not significant | OR = 1.21 |
|  | Leptin (high) | (42) | P = 0.042 | Not reported |
|  | Total cholesterol | (71) | P = 0.45 | Not reported |
|  |  | (77) | P = 0.13 | Not reported |
|  |  | (76) | P = 0.0637 | Not reported |
|  |  | (62) | P = < 0.05 | Not reported |
|  |  | (42) | P = 0.606 | Not reported |
|  | Total cholesterol/HDL | (76) | P = < 0.05 | Not reported |
|  |  | (42) | P = 0.189 | Not reported |
|  | Triglycerides | (71) | P = 0.55 | Not reported |
|  |  | (46) | Not significant | Not reported |
|  |  | (77) | P = 0.32 | Not reported |
|  |  | (72) | P = 0.037 | Not reported |
|  |  | (76) | P = 0.4156 | Not reported |
|  |  | (62) | P = <0.05 | Not reported |
|  |  | (76) | P = 0.1268 | Not reported |
|  | VLDL | (62) | P = 0.16 | Not reported |
|  |  | (78) | P = 0.015 | Not reported |
| **miRNA expression** | hsa-let-7b-3p (extracellular vesicle) | (78) | P = 0.018 | Not reported |
|  | hsa-let-7b-5p (extracellular vesicle) | (78) | P = 0.024 | Not reported |
|  | hsa-let-7e-5p (extracellular vesicle) | (78) | P = 0.032 | Not reported |
|  | hsa-miR-26a-5p (extracellular vesicle) | (78) | P = 0.045 | Not reported |
|  | hsa-miR-27a-3p (extracellular vesicle) | (78) | P = 0.032 | Not reported |
|  | hsa-miR-27b-3p (extracellular vesicle) | (78) | P = 0.045 | Not reported |
|  | hsa-miR-29a-3p (extracellular vesicle) | (78) | P = < 0.05 | Not reported |
|  | hsa-miR-30e-5p (extracellular vesicle) | (78) | P = 0.04 | Not reported |
|  | hsa-miR-92a-3p (extracellular vesicle) | (78) | P = 0.02 | Not reported |
|  | hsa-miR-92b-3p (extracellular vesicle) | (78) | P = 0.033 | Not reported |
|  | hsa-miR-98-5p (extracellular vesicle) | (78) | P = 0.022 | Not reported |
|  | hsa-miR-139-3p (extracellular vesicle) | (78) | P = 0.007 | Not reported |
|  | hsa-miR-146a-5p (extracellular vesicle) | (79) | P = < 0.05 | Not reported |
|  | miR-146a-5p in CD14+ monocytes | (78) | P = 0.01 | Not reported |
|  | hsa-miR-203a (extracellular vesicle) | (78) | P = 0.042 | Not reported |
|  | hsa-miR-486-5p (extracellular vesicle) | (78) | P = 0.017 | Not reported |
|  | hsa-miR-1180-3p (extracellular vesicle) | (78) | P = 0.039 | Not reported |
|  | hsa-miR-2379-5p (extracellular vesicle) | (78) | P = 0.022 | Not reported |
|  | hsa-miR-3158-3p (extracellular vesicle) | (78) | P = 0.018 | Not reported |
|  | hsa-miR-4732-3p (extracellular vesicle) | (95) | P = < 0.001 | Not reported |
| **Serum** | Anti-ADAMTS-L5 IgG | (95) | P = < 0.001 | Not reported |
|  | Anti-LL37 IgG | (82) | Not significant | Not reported |
|  | Anti-LL37 citrinullated | (82) | P = 0.02 | Not reported |
|  | Anti-LL37 carbamylated | (82) | P = 0.02 | Not reported |
|  | C9 | (54) | P = 3.63 E-01 | OR = 1.08 |
|  | CD5L | (53) | P = > 0.05 | Not reported |
|  | Creatinin | (43) | P 0.145 | Not reported |
|  |  | (44) | P = < 0.0001 | Not reported |
|  | Gelsolin | (83) | P = 0.0006 | Not reported |
|  | IFI16 | (61) | P = 0.05 | Not reported |
|  | IL2R | (54) | P = 3.05 E-06 | OR = 3.82 |
|  | ITGB5 | (84) | P = 0.0264 | Not reported |
|  | K17 | (54) | P = 3.07 E-04 | OR = 32.32 |
|  | M2BP | (85) | P = 0.0003 | Not reported |
|  | PRL | (84) | P = 0.050 | Not reported |
|  | STIP1 | (86) | P = 0.01 | OR = 4.28 |
|  | Uric acid | (88) | Not significant | Not reported |
|  |  | (77) | P = 0.001 | Not reported |
|  |  | (87) | P = 0.302 | Not reported |
|  | Hyperuricemia | (89) | P = 0.0098 | Not reported |
|  | VCP | (90) | P = 0.026 | Not reported |
|  | VEGFR-3 | (91) | P = < 0.0001 | Not reported |
|  | YKL-40 | (92) | P = < 0.001 | Not reported |
| **Skin** | C16ORF61, laesional 1 | (92) | P = 0.667 | Not reported |
|  | C16ORF61, laesional 2 | (92) | P = 0.007 | Not reported |
|  | C16ORF61, non-laesional | (92) | P = < 0.001 | Not reported |
|  | CP2N, laesional 1 | (92) | P = 0.032 | Not reported |
|  | CP2N, laesional 2 | (92) | P = 0.03 | Not reported |
|  | CP2N, non-laesional | (93) | P = 0.000 | Not reported |
|  | CXCL12 in blood vessels | (93) | P = 0.000 | Not reported |
|  | CXCL12 in dermal cells | (93) | P = 0.000 | Not reported |
|  | CXCL12 in keratinocytes | (92) | P = <0.001 | Not reported |
|  | FHL1, laesional 1 | (92) | P = 0.016 | Not reported |
|  | FHL1, laesional 2 | (92) | P = 0.021 | Not reported |
|  | FHL1, non-laesional | (92) | P = 0.014 | Not reported |
|  | GPS1, laesional 1 | (92) | P = 0.008 | Not reported |
|  | GPS1, laesional 2 | (92) | P = 0.385 | Not reported |
|  | GPS1, non-laesional | (94) | P = 0.001 | Not reported |
|  | IL23R, epidermal | (94) | P = 0.018 | Not reported |
|  | IL23R, dermal | (92) | P = 0.006 | Not reported |
|  | ITGB5, laesional 1 | (92) | P = 0.032 | Not reported |
|  | ITGB5, laesional 2 | (92) | P = 0.017 | Not reported |
|  | ITGB5, non-laesional | (92) | P = > 0.05 | Not reported |
|  | POSTN, laesional 1 | (92) | P = 0.001 | Not reported |
|  | POSTN, laesional 2 | (92) | P = 0.013 | Not reported |
|  | POSTN, non-laesional | (92) | P = 0.043 | Not reported |
|  | PPP2R4, laesional 1 | (92) | P = 0.008 | Not reported |
|  | PPP2R4, laesional 2 | (92) | P = 0.678 | Not reported |
|  | PPP2R4, non-laesional | (92) | P = < 0.001 | Not reported |
|  | SNCA, laesional 1 | (92) | P = < 0.001 | Not reported |
|  | SNCA, laesional 2 | (92) | P = 0.089 | Not reported |
|  | SNCA, non-laesional | (92) | P = 0.019 | Not reported |
|  | SRP14, laesional 1 | (92) | P = 0.016 | Not reported |
|  | SRP14, laesional 2 | (92) | P = 0.57 | Not reported |
|  | SRP14, non-laesional | (92) | P = 0.043 | Not reported |
|  | SRPX, laesional 1 | (92) | P = 0.08 | Not reported |
|  | SRPX, laesional 2 | (92) | P = 0.014 | Not reported |
|  | SRPX, non-laesional | (72) | P = 0.003 | Not reported |
| **Miscellaneous** | Arylesterase activity | (70) | P = 0.0011 | OR = 0.685 |
|  | Hemoglobin | (96) | P = < 0.001 | Not reported |
|  | IgG response to C region  of rM12 protein |  |  |  |

ACPA = anti citrullinated protein antibodies; ADAMTS = a disentegrin and metalloproteinase with thrombospondin motifs; anti-CCP = anti-cyclic citrullinated protein; Apo = apolipoprotein; C16ORF61 = endosomal protein sorting factor like (VSP35L); C2C = collagen fragment neoepitopes Col2-3/4 (long mono); C9 = complement factor 9; CCR = C-C chemokine receptor; CD = cluster of differentiation; CD5L = CD5 ligand; CER = ceramide; CM = central memory; COMP = cartilage oligometric matrix protein; CPII = C-propeptide of type II collagen; CP2N = carboxypeptidase N subunit 2; CRP = C-reactive protein; CTX = collagen type I C-telopeptide; CXCL = C-X-C motif ligand; CXCR = C-X-C motif receptor; DKK = dickkopf; EM = effector memory; ESR = erythrocyte sedimentation rate; FHL1 = four and a half LIM domains; GPS = G protein pathway suppressor; HAT = human airway trypsin-like protein; HDL = high density lipoprotein; hs = high sensitivity; IFI = interferon-inducible protein; IFN = interferon; Ig = immunoglobulin; IL = interleukin; IL2R = IL2 receptor; IL23R = interleukin 23 receptor; ISG = interferon stimulated gene; ITGB = integrin beta; K17 = keratin 17; L = liter; LDL = low density lipoprotein; M2BP = Mac-2-binding protein; M-CSF = macrophage colony stimulating factor; mg = milligram; miRNA = micro RNA; MMP = matrix metalloproteinase; mRNA = messenger RNA; NFKB = nuclear factor kappa-B; OPG = osteoprotegerin; OR = odds ratio; PBMC = peripheral blood mononuclear cells; POSTN = periostin; PPP2R4 = protein phosphatase 2 phosphatase activator (PTPA); RANKL = receptor activator of NFKB ligand; RNA = ribonucleic acid; SETD = SET domain protein; SNCA = synuclein alpha; SRP = signal recognition particle; SRPX = sushi repeat containing protein X-linked; STAT = signal transducer and activator of transcription; STIP = stress-inducible phosphoprotein; SYK = spleen associated tyrosine kinase; TBX = T-box; TNF = tumor necrosis factor; VCP = valosin containing protein; VEGFR = vascular endothelial growth factor receptor; VLDL = very low density lipoprotein
