# Supplementary material for: Clinical, laboratory, and genetic markers for the development or presence of psoriatic arthritis in psoriasis patients: a systematic review
Source: Arthritis Res Ther. 2021 Jun 14;23:168. doi: 10.1186/s13075-021-02545-4 (PMC8201808; doi:10.1186/s13075-021-02545-4)
Supplement: Supplementary file 5 — Additional file 5: Supplementary table 5. Statistical significance and effect sizes of genetic markers. [file 13075_2021_2545_MOESM5_ESM.docx]

**Supplementary table 5: Statistical significance and effect sizes of genetic markers**

| **Category** | **Marker** | **Study** | **Significance** | **Effect Size** |
| --- | --- | --- | --- | --- |
| **HLA** | Haplotype B*08:01-C*07 | (97) | P = 0.0020 | OR = 1.81 |
|  | Haplotype B*08-C*07-MICA*00801 | (98) | P = 0.021 | OR = 1.730 |
|  | Haplotype B*18-C*07 | (99) | P = 0.004 | OR = 10.1 |
|  | Haplotype B*27-C*01 | (97) | P = 0.0020 | OR = 4.61 |
|  |  | (99) | P = < 0.0001 | OR = 41.1 |
|  | Haplotype B*27-C*02 | (97) | P = 0.0333 | OR = 2.59 |
|  |  | (99) | P = < 0.0001 | OR = 19.9 |
|  |  | (100) | P = 0.04 | Not reported |
|  | Haplotype B*27-C*02-MICA*00701/026 | (98) | P = 0.000 | OR = 12.923 |
|  | Haplotype B*35-C*04-MICA*0201/020 | (98) | P = 0.047 | OR = 0.490 |
|  | Haplotype B*37-C*06 | (97) | P = 0.0424 | OR = 0.54 |
|  | Haplotype B*38-C*12 | (97) | P = 0.3865 | OR = 1.66 |
|  |  | (99) | P = 0.01 | OR = 2.9 |
|  |  | (100) | P = 0.02 | Not reported |
|  | Haplotype B*39:01-C*12 | (97) | P = 0.0190 | OR = 3.93 |
|  |  | (100) | P = 0.005 | Not reported |
|  | Haplotype B*57-C*06 | (97) | P = 0.0004 | OR = 0.49 |
|  |  | (99) | P = 0.03 | OR = 0.5 |
|  | Haplotype B*57-C*06-MICA*017 | (99) | P = 0.020 | OR = 0.577 |
|  | HLA-A3 Ashkenazi | (101) | P = < 0.05 | Not reported |
|  | HLA-A3 Sephardic | (101) | Not significant | Not reported |
|  | HLA-B*08 | (103) | P = > 0.05 | Not reported |
|  |  | (99) | P = 0.009 | OR = 1.61 |
|  |  | (100) | P = 0.12 | Not reported |
|  |  | (102) | P = 1.76x10^E^-3^*^ | Not reported |
|  | HLA-B*08:01 | (97) | P = 0.0019 | OR = 1.81 |
|  |  | (102) | P = 1.76x10^E^-3^*^ | Not reported |
|  | HLA-B*13 Ashkenazi | (101) | P = < 0.05 | Not reported |
|  | HLA-B*13 Sephardic | (101) | Not significant | Not reported |
|  | HLA-B*13 | (104) | Not significant | Not reported |
|  |  | (102) | P = 1.72x10^E^-3^*^ | Not reported |
|  | HLA-B*18 | (100) | P = 0.52 | Not reported |
|  | HLA-B*18:01:01 | (97) | P = 0.0037 | OR = 6.59 |
|  | HLA-B*27 | (105) | P = 0.0007 | Not reported |
|  |  | (99) | P = < 0.0001 | OR = 5.17 |
|  |  | (100) | P = 0.002 | Not reported |
|  |  | (104) | P = < 0.001 | OR = 4.2 |
|  |  | (102) | P=7.96x10^E^-7^*^ | Unclear |
|  | HLA-B*27:05 | (103) | P = 3.53 x 10^E^-7 | OR = 2.34 |
|  | HLA-B*27:05:02 | (97) | P = 0.0001 | OR = 3.77 |
|  | HLA-B*37 | (102) | P = 1.05x10^E^-2^*^ | Not reported |
|  | HLA*B37:01 | (102) | P = 1.05x10^E^-2^*^ | Not reported |
|  | HLA-B*37:01:01 | (97) | P = 0.0424 | OR = 0.54 |
|  | HLA-B*38 | (99) | P = 0.026 | OR = 1.65 |
|  |  | (100) | P = 0.04 | Not reported |
|  |  | (104) | Not significant | Not reported |
|  | HLA-B*38 Ashkenazi | (101) | Not significant | Not reported |
|  | HLA-B*38 Sephardic | (101) | P = < 0.05 | Not reported |
|  | HLA-B*38:01:01 | (97) | P = 0.3865 | OR = 1.66 |
|  | HLA-B*39 | (100) | P = 0.03 | Not reported |
|  | HLA-B*39:01:01:01 | (97) | P = 0.0288 | OR = 2.86 |
|  | HLA-B*39:06:01 | (97) | P = 1 | OR = 1.20 |
|  | HLA-B*44:02:01:01 | (97) | P = 0.0198 | OR = 0.60 |
|  | HLA-B*57 | (99) | P = 0.001 | OR = 0.58 |
|  |  | (100) | P = 0.47 | Not reported |
|  |  | (104) | Not significant | Not reported |
|  |  | (102) | P = 2.64x10^E^-2^*^ | Not reported |
|  | HLA-B57*01 | (102) | P = 1.98 x 10^E^-2^*^ | Not reported |
|  | HLA-B*57:01:01 | (97) | P = 0.0002 | OR = 0.48 |
|  | HLA-B*70 Ashkenazi | (101) | P = < 0.05 | Not reported |
|  | HLA-B*70 Sephardic | (101) | Not significant | Not reported |
|  | HLA-B amino acid position 45 Glu | (102) | P = 1.46 x 10^E^-4^*^ | Not reported |
|  | HLA-B amino acid position 45 Glu/Gly | (102) | P = 2.02 x 10^E^-4^*^ | Not reported |
|  | HLA-B amino acid position 45 Glu/Lys | (102) | P = 7.89x10^E^-3^*^ | Not reported |
|  | HLA-B amino acid position 45 Glu/Thr | (102) | P = 2.24 x 10^E^-3^*^ | Not reported |
|  | HLA-B amino acid position 45  Glu vs Thr/Lys/Met | (106) | P = 2.9 x 10^E^-12 | OR = 1.46 |
|  |  | (103) | P = 0.16 | Not reported |
|  | HLA-B amino acid position 45 Gly/Met | (102) | P = 3.57x 10^E^-3^*^ | Not reported |
|  | HLA-B amino acid position 45 Lys/Met | (102) | P = 2.62x10^E^-3^*^ | Not reported |
|  | HLA-B amino acid position 45 Lys/Thr | (102) | P = 1.74x10^E^-2^*^ | Not reported |
|  | HLA-B amino acid position 95 Leu | (102) | P = 3.50 x 10^E^-8 | OR = 1.595 |
|  | HLA-B amino acid position 95 Trp | (102) | P = 3.18 x 10^E^-3^*^ | Not reported |
|  | HLA-B amino acid position 97 Arg/Asn/Ser | (102) | P = 1.47 x 10^E^-6^*^ | Not reported |
|  | HLA-B amino acid position 97 Arg/Thr | (102) | P = 1.20 x 10^E^-2^*^ | Not reported |
|  | HLA-B amino acid position 97 Asn | (102) | P = 4.73 x 10E-6^*^ | Not reported |
|  | HLA-B amino acid position 97 Asn/Ser | (102) | P =1.31 x 10^E^-6^*^ | Not reported |
|  | HLA-B amino acid position 97 Asn/Ser/Thr | (102) | P = 1.62 x 10^E^-3^*^ | Not reported |
|  | HLA-B amino acid position 97 Asn/Ser/Trp | (102) | P = 9.47 x 10^E^5^*^ | Not reported |
|  | HLA-B amino acid position 97 Asn/Trp | (102) | P = 3.92 x 10^E^-2^*^ | Not reported |
|  | HLA-B amino acid position 97 Asn/Ser/Val | (102) | P = 2.12 x 10^E^-3^*^ | Not reported |
|  | HLA-B amino acid position 97 Asp vs Arg | (103) | P = 5.76 x 10^E^-8 | OR = 2.46 |
|  | HLA-B amino acid position 97 Ser | (102) | P = 1.37 x 10^E^-2^*^ | Not reported |
|  | HLA-B amino acid position 97 Ser/Trp | (102) | P = 4.82 x 10^E^-2^*^ | Not reported |
|  | HLA-B amino acid position 97 Ser vs Arg | (103) | P = 3.58 x 10^E^-5 | OR = 1.45 |
|  | HLA-B amino acid position 97 | (102) | P = 2.74 x 10^E^-2^*^ | Not reported |
|  | HLA-B amino acid position 97 Thr/Trp | (102) | P = 1.33 x 10^E^-2 | Not reported |
|  | HLA-B amino acid position 97 Thr/Val | (102) | P = 2.49 x 10^E^-3^*^ | Not reported |
|  | HLA-B amino acid position 97 Thr vs Arg | (103) | P = 0.716 | OR = 0.959 |
|  | HLA-B amino acid position 97 Try vs Arg | (103) | P = 0.283 | OR = 0.795 |
|  | HLA-B amino acid position 97 Trp/Val | (102) | P = 2.35 x 10^E^-2^*^ | Not reported |
|  | HLA-B amino acid position 97 Val | (102) | P = 3.89 x 10^E^-2^*^ | Not reported |
|  | HLA-B amino acid position 97 Val vs Arg | (103) | P = 0.913 | OR = 0.988 |
|  | HLA-C*01 | (99) | P = 0.001 | OR = 2.54 |
|  |  | (100) | P = 0.21 | Not reported |
|  |  | (102) | P = 3.43 x 10^E^-3^*^ | Not reported |
|  | HLA-C*01:02 | (102) | P = 3.43 x10^E^-3^*^ | Not reported |
|  | HLA-C*01:02:01 | (97) | P = 0.0828 | OR = 1.78 |
|  | HLA-C*02 | (99) | P = 0.0008 | OR = 2.42 |
|  |  | (100) | P = 0.27 | Not reported |
|  |  | (102) | P = 2.40 x 10^E^-2^*^ | Not reported |
|  | HLA-C*02:02 | (102) | P = 2.40 x 10^E^-2^*^ | Not reported |
|  | HLA-C*02:02:02 | (97) | P = 0.0316 | OR = 2.35 |
|  | HLA-C*06 | (99) | P = 0.0002 | OR = 0.58 |
|  |  | (105) | P = 0.014 | OR = 0.41 |
|  |  | (100) | P = 0.69 | Not reported |
|  |  | (108) | P = 0.02 | OR = 0.72 |
|  |  | (104) | P = < 0.001 | OR = 0.5 |
|  |  | (107) | P = < 0.001 | Not reported |
|  |  | (102) | P = 6.96 x 10^E^-11 | OR = 0.5275 |
|  | HLA-C*06 Ashkenazi | (101) | P = < 0.05 | Not reported |
|  | HLA-C*06 Sephardic | (101) | Not significant | Not reported |
|  | HLA-C*06:02 | (103) | P = 9.57 x 10^E^-66 | OR = 0.37 |
|  |  | (109) | p = 0.491 | Not reported |
|  |  | (102) | P = 6.96 x10^E^-11 | OR = 0.5275 |
|  | HLA-C*06:02:01:01 | (97) | P = 9.94 x 10^E^-12 | OR = 0.30 |
|  | HLA-C*07 | (99) | P = 0.027 | OR = 1.35 |
|  |  | (100) | P = 0.32 | Not reported |
|  |  | (102) | P = 2.21 x 10^E^-4^*^ | Not reported |
|  | HLA-C*07:01 | (102) | P = 8.27 x 10^E^-3^*^ | Not reported |
|  | HLA-C*07:01:01:01 | (97) | P = 0.0023 | OR = 1.76 |
|  | HLA-C*07:02 | (102) | P = 3.05 x 10^E^-2^*^ | Not reported |
|  | HLA-C*08 | (105) | P = 0.021 | OR = 0.35 |
|  | HLA-C*12 | (99) | P = 0.13 | OR = 1.29 |
|  |  | (100) | P = 0.005 | Not reported |
|  | HLA-C*12:03:01:01 | (97) | P = 0.0668 | OR = 1.83 |
|  | HLA-C amino acid position 305 Ala | (102) | P = 4.47 x 10^E^-8 | OR = 1.582 |
|  | HLA-C amino acid position 305 Thr | (102) | P = 2.21 x 10^E^-4^*^ | Not reported |
|  | HLA-C rs10484554 | (110) | P = 1.69 x 10^E^-6 | Not reported |
|  | HLA-C rs12191877 | (111) | P = 0.006 | Not reported |
|  | HLA-DQB1*02:01 | (102) | P= 3.25 x 10^E^-3^*^ | Not reported |
|  | HLA-DQB1*02:01 Ashkenazi | (101) | P = < 0.05 | Not reported |
|  | HLA-DQB1*02:01 Sephardic | (101) | Not significant | Not reported |
|  | HLA-DRB1*03 | (102) | P = 4.03 x 10^E^3^*^ | Not reported |
|  | HLA-DRB1*03:01 | (102) | P = 3.06 x 10^E^3^*^ | Not reported |
|  | HLA-DRB1*03:01 Ashkenazi | (101) | Not significant | Not reported |
|  | HLA-DRB1*03:01 Sephardic | (101) | Not significant | Not reported |
|  | HLA-DRB1*04:02 Ashkenazi | (101) | P = < 0.05 | Not reported |
|  | HLA-DRB1*04:02 Sephardic | (101) | Not significant | Not reported |
|  | HLA-DRB1*04:05 Ashkenazi | (101) | P = < 0.05 | Not reported |
|  | HLA-DRB1*04:05 Sephardic | (101) | P = < 0.05 | Not reported |
|  | HLA-DRB1*04:06 Ashkenazi | (101) | P = < 0.05 | Not reported |
|  | HLA-DRB1*04:06 Sephardic | (101) | P = < 0.05 | Not reported |
|  | HLA-DRB1*07 | (105) | P = < 0.001 | OR = 0.12 |
|  | HLA-DRB1*14:01 Ashenazi | (101) | P = < 0.05 | Not reported |
|  | HLA-DRB1*14:01 Sephardic | (101) | Not significant | Not reported |
|  | rs1050414 (near HLA-C and HLA-B) | (120) | P = 7.4 x 10^E^-11 | OR = 1.53 |
| **Non-HLA** | *ADAMTS9-MAG1* deletion | (112) | P = 0.0088 | Not reported |
|  | *CCR2* rs1799864 | (113) | P = 0.0007 | Not reported |
|  | *IL1RN* rs397211 | (111) | P = 0.79 | Not reported |
|  |  | (114) | P = 0.74 | Not reported |
|  | *IL12B* rs2082412 | (114) | P = 0.04 | Not reported |
|  |  | (111) | P = 0.01 | Not reported |
|  | *IL12B* rs3212227 | (109) | P = 0.549 | Not reported |
|  |  | (115) | P = 0.55 | OR = 1.13 |
|  | *IL12B* rs6887695 | (109) | P = 0.522 | Not reported |
|  |  | (115) | P = 0.33 | OR = 1.20 |
|  | *IL13* rs1800925 | (116) | P = 0.045 | OR = 1.28 |
|  |  | (117) | P = 0.015 | Not reported |
|  | *IL13* rs20541 | (114) | P = 0.48 | Not reported |
|  |  | (117) | P = 0.004 | Not reported |
|  |  | (111) | P = 0.11 | Not reported |
|  | *IL13* rs848 | (116) | P = 0.047 | RR = 1.30 |
|  | *IL17E* rs79877597 | (118) | P = 0.032 | OR = 1.50 |
|  | *IL23A* rs2066807 | (114) | P = 0.96 | Not reported |
|  |  | (111) | P = 0.23 | Not reported |
|  | *IL23R* rs11209026 | (109) | P = 0.459 | Not reported |
|  |  | (115) | P = 0.11 | OR = 1.96 |
|  | *IL23R* rs2201841 | (114) | P = 0.08 | Not reported |
|  |  | (111) | P = 0.02 | Not reported |
|  | *IL23R* rs 7530511 | (109) | P = 0.994 | Not reported |
|  | KIR2DS1 pos / C2 neg | (119) | P = 0.0046 | Not reported |
|  | MICA*00701/026 (presence) | (98) | P = < 0.001 | OR = 4.402 |
|  | MICA*00801 (presence) | (98) | P = 0.110 | OR = 1.339 |
|  | MICA*00801 (homozygosity) | (98) | P = 0.009 | OR = 2.260 |
|  | MICA*016 (presence) | (98) | P = 0.034 | OR = 0.418 |
|  | *NFKBIA* rs696 | (115) | P = 0.1 | OR = 1.36 |
|  | *NFKBIA* rs7152376 | (107) | P = < 0.001 | Not reported |
|  | *NFKBIA* rs8016957 | (114) | P = 0.06 | Not reported |
|  | *PTPN22* rs2476601 | (121) | P = 4.4 x 10^E^-4 | Not reported |
|  |  | (115) | P = 0.41 | OR = 1.21 |
|  | rs4891505 (near LOC100505817) | (120) | p = 6.7 x 10^E^-9 | OR = 1.63 |
|  | TNFa-238 | (122) | P = 0.99 | OR = 1.002 |
|  |  | (109) | P = 0.577 | Not reported |
|  | TNFa-308 | (122) | P = 0.93 | OR = 1.04 |
|  |  | (109) | P = 0.673 | Not reported |
|  | TNFa-857 | (109) | p = 0.038 | Not reported |
|  | TNFa-1031 | (109) | P = 0.657 | Not reported |
|  | TNFacd haplotype a2c2d4 | (123) | Not significant | Not reported |
|  | TNFacd haplotype a6c1d3 | (123) | P = 0.008 | RR = 5.3 |
|  | TNFacd haplotype a10c1d3 | (123) | Not significant | Not reported |
|  | TNFacd haplotype a11c1d3 | (123) | Not significant | Not reported |
|  | *TNFAIP3* rs610604 | (114) | P = 0.58 | Not reported |
|  |  | (111) | P = 0.67 | Not reported |
|  | *TNIP1* rs17728338 | (114) | P = 0.07 | Not reported |
|  |  | (111) | P = 0.07 | Not reported |
|  | *TSC1* rs1076160 | (114) | P = 0.42 | Not reported |
|  |  | (111) | P = 0.52 | Not reported |
|  | ZNF816A | (114) | P = 0.01 | Not reported |

^*^ no correction for multiple testing, not genome-wide significant

ADAMTS = a disentegrin and metalloproteinase with thrombospondin motifs; Ala = alanine; Arg = Arganine; Asn = Asparagine; CCR = C-C motif receptor; DNA = deoxyribonuclease acid; Glu = glutamic acid; Gly = glycine; HLA = human leukocyte antigen; IL = interleukin; IL1RN = IL-1 receptor antagonist; IL23R = IL-23 receptor; KIR = killer-cell immunoglobuline-like receptor; Leu = leucine; Lys = lysine; MAGI = membrane-associated guanylate kinase; Met = methionine; MHC = major histocompatibility complex; MICA = MHC class I polypeptide-related sequence A; NFKB = nuclear factor kappa B; NFKBIA = NFKB inhibitor alpha; OR = odds ratio; ; PTPN22 = protein tyrosine phosphatase non-receptor type 22; Ser = Serine; Thr = threonine; TNF = tumor necrosis factor; TNFAIP = TNF alpha-induced protein; TNIP = TNFAIP3 interacting protein; Trp = tryptophan; TSC1 = tuberous sclerosis 1; Val = valine; ZNF = zinc finger protein
