# Supplementary material for: Clinical, laboratory, and genetic markers for the development or presence of psoriatic arthritis in psoriasis patients: a systematic review
Source: Arthritis Res Ther. 2021 Jun 14;23:168. doi: 10.1186/s13075-021-02545-4 (PMC8201808; doi:10.1186/s13075-021-02545-4)
Supplement: Supplementary file 6 — Additional file 6: Supplementary table 6. Quality assessment of cohort studies. [file 13075_2021_2545_MOESM6_ESM.docx]

**Supplementary table 6: Quality assessment of cohort studies**

| **Article** | **Selection** | | | | **Comparibility** | | **Outcome** | | | **Conclusion** |
| --- | --- | --- | --- | --- | --- | --- | --- | --- | --- | --- |
|  | **Representativeness of exposed cohort** | **Selection of non-exposed cohort** | **Ascertainment of exposure** | **Outcome not present at start** | **Controls  for age** | **Controls for additional factor** | **Assessment of outcome** | **Sufficient follow-up** | **Adequacy of  follow-up** | **Quality** |
| Abji, 2016^27^ | A | A | A | A | yes | yes | A | A | C | Fair |
| Abji, 2020^60^ | A | A | A | A | no | yes | A | A | C | Fair |
| Eder, 2016^18^ | A | A | B | A | no | yes | A | A | B | Good |
| Eder, 2017^22^ | A | A | B | A | yes | yes | A | A | B | Good |
| Egeberg, 2018^23^ | A | A | A | B | no | yes | B | A | A | Good |
| Green, 2020^29^ | A | A | A | B | yes | yes | B | A | A | Good |
| Lewinson, 2017^36^ | A | A | A | B | yes | yes | B | A | C | Fair |
| Li, 2012^32^ | C | A | C | B | yes | yes | C | A | C | Poor |
| Li, 2012^34^ | C | A | C | B | yes | yes | C | A | B | Poor |
| Love, 2012^33^ | A | A | A | B | yes | yes | B | A | A | Good |
| Nguyen, 2018^31^ | C | A | A | B | yes | yes | B | A | A | Fair |
| Simon, 2020^26^ | A | A | A | A | yes | yes | A | A | A | Good |
| Soltani, 2010^24^ | B | B | A | B | no | yes | D | A | A | Fair |
| Thorarensen, 2017^35^ | A | A | A | B | no | no | B | A | D | Poor |
| Wilson, 2009^25^ | A | B | A | B | yes | yes | B | A | A | Fair |
| Wu, 2015^30^ | C | A | C | B | yes | no | C | A | B | Poor |

*Risk of bias was assessed using the Newcastle-Ottawa scale and for further explanation of the exact answer options, we refer to the original paper^16^ (ref 16). A study was considered of “good” quality when it had a minimum of 3 stars in the selection domain, 1 star in the comparability domain and 2 stars in the outcome/exposure domain. “Fair” quality was given when a study had a minimum of 2 stars in the selection, 1 star in the compatibility and 2 stars in the outcome/exposure domain^17^ .*
