# Supplementary material for: Clinical, laboratory, and genetic markers for the development or presence of psoriatic arthritis in psoriasis patients: a systematic review
Source: Arthritis Res Ther. 2021 Jun 14;23:168. doi: 10.1186/s13075-021-02545-4 (PMC8201808; doi:10.1186/s13075-021-02545-4)
Supplement: Supplementary file 7 — Additional file 7: Supplementary table 7. Quality assessment of case control studies. [file 13075_2021_2545_MOESM7_ESM.docx]

**Supplementary table 7: Quality assessment of case control studies**

| **Article** | **Selection** | | | | **Comparibility** | | **Exposure** | | | **Conclusion** |
| --- | --- | --- | --- | --- | --- | --- | --- | --- | --- | --- |
|  | **Adequate case definition** | **Representativeness of cases** | **Selection of controls** | **Definition of controls** | **Controls for age** | **Controls for additional factor** | **Ascertainment of exposure** | **Method of ascertainment** | **Non response rate** | **Quality** |
| Abdelaal, 2018^93^ | C | B | C | B | yes | yes | C | A | A | Poor |
| Abdel Fattah, 2009^37^ | A | A | A | B | no | no | A | A | A | Poor |
| Abji, 2017^80^ | A | B | A | A | yes | yes | A | A | A | Good |
| Alenius, 2005^39^ | C | A | A | B | no | no | A | A | A | Poor |
| Alenius, 2009^63^ | A | B | A | A | no | no | A | A | A | Poor |
| Amin, 2015^57^ | A | A | A | B | no | no | A | A | A | Good |
| De Andrea, 2019^83^ | A | A | C | B | yes | yes | A | A | A | Fair |
| Aterido, 2019^102^ | A | A | C | B | no | no | A | B | C | Poor |
| Attia, 2011^55^ | A | A | A | A | no | no | A | A | A | Poor |
| Ausavarungnirun, 2017^66^ | A | B | A | B | yes | yes | A | A | A | Fair |
| Barbarroja, 2019^87^ | A | A | A | A | yes | yes | A | A | A | Good |
| Bartosinka, 2015^50^ | C | B | A | B | no | no | A | A | C | Poor |
| Bartosinka, 2018^133^ | C | B | A | B | yes | yes | A | A | A | Poor |
| Batalla, 2015^118^ | A | B | A | A | no | no | A | A | A | Poor |
| Benham, 2013^59^ | C | B | C | B | no | no | C | A | A | Poor |
| Borman, 2008^47^ | B | A | A | B | no | no | A | A | A | Poor |
| Bose, 2014^58^ | A | B | A | B | no | no | C | A | C | Poor |
| Bostoen, 2014^124^ | A | A | A | B | no | no | A | A | A | Good |
| Bowes, 2011^117^ | A | B | C | B | no | no | A | A | C | Poor |
| Bowes, 2015^121^ | B | B | C | B | no | yes | A | B | A | Fair |
| Bowes, 2017^103^ | A | A | C | B | no | yes | A | B | B | Poor |
| Cabaleiro, 2013^109^ | C | B | A | B | no | yes | A | A | A | Poor |
| Calzavara, 1998^125^ | A | B | C | B | no | no | B | A | A | Poor |
| Candia, 2006^38^ | A | B | C | A | no | no | A | A | A | Poor |
| Canpolat, 2010^68^ | C | B | A | B | no | no | A | A | A | Poor |
| Chandran, 2010^49^ | A | B | C | A | yes | yes | A | A | A | Fair |
| Coto-Segura, 2019^107^ | A | B | A | B | no | yes | A | A | A | Fair |
| Cretu, 2015^92^ | C | B | C | A | yes | yes | A | A | A | Poor |
| Cretu, 2017^54^ | A | B | C | A | yes | yes | A | A | A | Fair |
| Dalbeth, 2010^53^ | A | B | B | B | no | no | A | A | A | Poor |
| Dalmady, 2013^41^ | A | B | C | A | no | no | A | A | A | Poor |
| Diani, 2019^67^ | C | B | A | B | no | no | C | A | A | Poor |
| Diani, 2019^51^ | A | B | A | B | no | no | A | A | A | Poor |
| Eder, 2011^20^ | A | B | A | A | yes | yes | D | A | A | Good |
| Eder, 2011^116^ | A | B | B | A | no | yes | A | A | C | Fair |
| Eder, 2012^28^ | A | B | A | A | yes | yes | C | A | B | Poor |
| Eder, 2012^100^ | A | A | A | A | no | no | A | A | A | Poor |
| Eder, 2012^99^ | A | A | C | A | no | no | A | A | A | Poor |
| Eder, 2012^126^ | A | A | A | A | no | no | A | A | A | Poor |
| Eder, 2013^71^ | A | A | A | A | yes | yes | A | A | A | Good |
| Eiris, 2014^134^ | A | B | A | B | no | no | A | A | A | Poor |
| Elkayam, 2004^101^ | A | A | C | B | no | no | A | A | A | Poor |
| Engin, 2020^127^ | C | B | A | B | no | no | A | A | A | Poor |
| Esawy, 2019^44^ | A | B | A | B | no | no | A | A | A | Poor |
| Farrag, 2017^131^ | A | A | A | A | no | no | A | A | A | Poor |
| Frasca, 2018^82^ | A | B | A | B | no | no | A | A | C | Poor |
| Gisondi, 2011^45^ | C | A | A | B | no | no | A | A | A | Poor |
| Hein, 1991^48^ | C | B | A | B | no | no | A | A | A | Poor |
| Ho, 2008^108^ | A | A | C | B | no | no | A | A | C | Poor |
| Hohler, 2002^123^ | A | B | C | B | no | no | A | A | C | Poor |
| Hong, 2018^90^ | C | B | C | B | no | no | A | A | C | Poor |
| Hur, 2020^75^ | C | B | A | B | no | no | A | A | A | Poor |
| Husakova, 2015^85^ | A | B | C | A | no | no | A | A | A | Poor |
| Husni, 2018^72^ | A | A | A | B | no | no | A | A | C | Poor |
| Isik, 2016^122^ | C | B | A | B | no | no | A | A | A | Poor |
| Jadon, 2017^52^ | A | A | C | B | yes | yes | A | A | B | Fair |
| Jensen, 2013^91^ | A | B | C | B | no | no | A | A | A | Poor |
| Johnson, 2019^64^ | B | B | A | B | yes | yes | A | A | A | Poor |
| Julia, 2012^110^ | A | B | C | B | no | yes | A | A | A | Poor |
| Julia, 2015^112^ | A | A | C | B | no | no | A | A | C | Poor |
| Kilic, 2017^69^ | B | B | A | B | no | no | D | A | A | Poor |
| Kim, 2016^70^ | B | B | A | B | no | no | A | A | A | Poor |
| Krajewska, 2019^74^ | A | B | C | B | no | no | A | A | A | Poor |
| El-Leithy, 2020^94^ | A | B | A | B | no | no | C | A | A | Poor |
| Li, 2017^56^ | A | B | C | B | no | no | A | A | A | Poor |
| Liao, 2008^105^ | A | A | A | A | no | no | A | A | A | Poor |
| Lin, 2014^73^ | A | B | A | A | no | no | A | A | A | Poor |
| Lin, 2019^79^ | A | B | C | A | no | no | A | A | A | Poor |
| Loft, 2018^115^ | A | A | C | B | yes | yes | A | A | A | Fair |
| Maejima, 2014^84^ | C | B | C | B | no | no | C | A | A | Poor |
| Maejima, 2017^89^ | B | B | C | B | no | no | C | A | A | Poor |
| Mavropoulos, 2017^129^ | A | A | A | B | no | no | C | A | A | Poor |
| Muto, 1996^96^ | B | B | C | B | no | no | A | A | A | Poor |
| Mysliwiec, 2017^46^ | B | B | A | B | no | no | A | A | A | Poor |
| Mysliwiec, 2019^130^ | C | B | A | B | no | no | A | A | A | Poor |
| Nair, 2009^111^ | C | A | A | B | no | no | A | A | A | Poor |
| Okada, 2014^106^ | A | A | C | B | no | no | A | A | C | Poor |
| Orgaz-Molina, 2013^42^ | A | B | A | B | yes | yes | A | A | A | Poor |
| Ortolan, 2019^77^ | A | A | A | B | no | no | A | A | A | Poor |
| Pasquali, 2020^78^ | A | B | A | A | yes | yes | A | A | A | Good |
| Pattison, 2008^21^ | A | B | C | B | yes | yes | D | A | C | Poor |
| Pietrzak, 2018^76^ | C | B | A | B | no | no | A | A | A | Poor |
| Pietrzak, 2020^62^ | C | B | A | B | no | no | A | A | A | Poor |
| Pirowska, 2018^65^ | C | B | A | B | no | no | A | A | A | Poor |
| Pollock, 2011^98^ | A | B | B | A | no | yes | A | A | C | Fair |
| Pollock, 2013^104^ | A | B | A | A | no | no | A | A | A | Poor |
| Pollock, 2015^81^ | C | B | C | B | no | yes | A | A | A | Poor |
| Pollock, 2019^136^ | A | B | A | A | no | no | A | A | A | Poor |
| Sag, 2018^43^ | A | A | A | B | yes | yes | A | A | A | Good |
| Shibata, 2009^40^ | B | B | A | B | yes | yes | A | A | A | Poor |
| Soto-Sanchez, 2010^113f^ | A | B | A | A | yes | yes | A | A | A | Good |
| Spadaro, 1996^61^ | A | A | C | B | no | yes | A | A | A | Fair |
| Stuart, 2015^120^ | C | A | C | B | no | no | A | A | C | Poor |
| Thumboo, 2018^19^ | B | A | A | B | yes | yes | D | A | A | Good |
| Tsuruta, 2017^86^ | B | A | A | B | yes | yes | D | A | A | Fair |
| Voiculescu, 2018^137^ | C | B | A | B | no | no | A | A | A | Poor |
| Williams, 2005^119^ | C | B | C | B | no | no | A | A | A | Poor |
| Winchester, 2012^97^ | A | B | C | A | no | no | A | A | A | Poor |
| Yan, 2018^138^ | B | B | A | B | yes | yes | A | A | A | Fair |
| Yang, 2012^114^ | A | B | A | A | no | no | A | A | A | Poor |
| Yilmaz, 2017^88^ | C | B | A | B | no | no | A | A | A | Poor |
| Yuan, 2019^95^ | C | B | C | B | no | no | A | A | C | Poor |
| Zhao, 2019^139^ | A | B | A | A | no | yes | A | A | A | Good |

*Risk of bias was assessed using the Newcastle-Ottawa scale and for further explanation of the exact answer options, we refer to the original paper^16^. A study was considered of “good” quality when it had a minimum of 3 stars in the selection domain, 1 star in the comparability domain and 2 stars in the outcome/exposure domain. “Fair” quality was given when a study had a minimum of 2 stars in the selection, 1 star in the compatibility and 2 stars in the outcome/exposure domain^17^ .*
